# Broad-scale shift in dominance of arbuscular mycorrhizal trees along the Japanese archipelago associated with tree diseases and climate warming

**DOI:** 10.1038/s41467-026-77711-w

**Published:** 2026-09-11

**Authors:** Holger Schaefer, Naoyuki Yamashita, Shoji Hashimoto, Yoshiyuki Inagaki, Ayumi Kawanishi, Satoru Chatani, Hikari Shimadera, Hitomi Furusawa, Akihiro Imaya

**Affiliations:** 1 Department of Forest Soils, Forestry and Forest Products Research Institute (FFPRI), Tsukuba, Japan; 2 Shikoku Research Center, Forestry and Forest Products Research Institute (FFPRI), Kochi, Japan; 3 Graduate School of Agricultural and Life Sciences, The University of Tokyo, Tokyo, Japan; 4 National Institute for Environmental Studies (NIES), Tsukuba, Japan; 5 Graduate School of Engineering, The University of Osaka, Suita, Japan

**Keywords:** Forest ecology, Biogeochemistry, Climate-change ecology, Microbial ecology

## Abstract

Distribution shifts of mycorrhizal tree symbioses are predicted to alter the nutrient cycling in temperate forests worldwide. Yet, our understanding of the drivers and specific impacts of such shifts remains limited. Here, we assign mycorrhizal types to trees at the species and genus level in >13,000 forest plots to assess the dominance of arbuscular mycorrhizal trees based on basal area proportions along the Japanese archipelago in temperate East Asia. We show a significant increase in arbuscular mycorrhizal tree dominance within 15 years associated with climate warming and the spread of tree diseases, contributing to a more important role of arbuscular mycorrhizal symbiosis in forest ecosystems than previously predicted. Arbuscular mycorrhizal tree dominance is negatively related to the ratio of soil carbon and nitrogen stocks, suggesting that tree symbiosis shifts will affect soil nitrogen availability and retention. In light of the global surge of biotic forest disturbance, our results stress the importance of forest pest and disease control to decelerate tree symbiosis shifts and their impact on ecosystem functioning.

## Abstract (web summary)

Mycorrhizal associations with trees help regulate nutrient cycling in forests. This study found that the dominance of arbuscular mycorrhizal trees across Japan is associated with climate warming and tree diseases and may influence forest nitrogen cycling.

## Introduction

Mycorrhizal symbioses, in which root-associated fungi provide plants with soil nutrients in exchange for photosynthates^1^, are ubiquitous across terrestrial ecosystems^2^. By altering how plants cycle nutrients^3^, compete for resources^4^, and tolerate environmental stress^5^, mycorrhizal symbioses impact plant community development and ecosystem functions such as carbon (C) sequestration on a global scale^6–8^. Most forest trees are symbiotic with either ectomycorrhizal (EM) or arbuscular mycorrhizal (AM) fungi^9^, which differ in how they provide soil nutrients to plants. EM fungi tend to directly acquire soil organic nitrogen (N) via extracellular enzymes, which slows down saprotrophic soil organic matter (SOM) mineralization^3,10–12^. In contrast, AM fungi primarily acquire inorganic forms of N and phosphorus and, hence, benefit from the rapid mineralization of SOM^3,12,13^. The distribution of mycorrhizal tree symbioses shows a distinct latitudinal gradient, i.e., from AM tree-dominated forests in decomposition-enhancing, warm and wet climates to EM tree-dominated forests in decomposition-inhibiting, cold and dry climates^9^, highlighting the close link between mycorrhizal nutrient acquisition and tree distributions.

Owing to climate warming, a shift from EM to AM trees is predicted to occur in temperate and boreal forests worldwide^9^. In North America, anthropogenic N pollution of forest soils was shown to promote this shift^14,15^. A spread of AM trees was suggested to accelerate forest nutrient cycling in North America, potentially altering forest C sequestration, N retention, and productivity^15^. Yet, our understanding of tree symbiosis distribution dynamics worldwide remains limited. In a recently published global tree symbiosis map, >75% of the tree inventory data were collected from North America and Europe^9^. Temporal changes of tree symbiosis distributions have, to our knowledge, so far only been assessed in North America^14,15^. This bias in tree symbiosis studies limits our ability to generalize observed dynamics and relationships^16^. Indeed, relationships between mycorrhizal distributions, climate, and soil nutrient status were shown to differ by ecoregion and biome^15,17^. Hence, it is crucial to assess and verify the dynamics of tree symbiosis distributions in less-studied environments to understand and predict changes in forest nutrient cycling and their climate feedbacks.

In East Asia, AM and EM tree distributions and their temporal changes along larger latitudinal gradients remain poorly understood. At the same time, significant predictors of the AM tree dominance shift in North America such as fire disturbance and N deposition^15^ are likely less important in larger parts of East Asia, as the proportion of burned tree-covered areas is generally smaller^18^ and precipitation-rich areas show reduced accumulation of deposited N^19^. In contrast, tree diseases and pests such as the pine wilt disease (PWD) have been identified as potent forest disturbance agents that affect tree species distributions in Japan, China, and South Korea^20–23^ and may, thus, influence tree symbiosis distributions. In this study, we analyze time-series data from over 13,000 natural and planted forest plots between 24 and 45°N in Japan’s National Forest Inventory (NFI; 1999–2019), to reveal the spatial and temporal change in AM tree dominance in precipitation-rich forests of temperate East Asia and its relationships to climate, soil nutrient status, and forest disturbance. In line with previous studies^9,15,24^, AM tree dominance is calculated from the basal-area proportion of AM tree species in each forest plot after assigning mycorrhizal types at the species or genus level. For tree species that are assigned two mycorrhizal types (e.g., AM-EM), half of their basal area is included in AM tree dominance estimates.

We hypothesize that the spatial distribution of AM tree dominance in temperate forests is mostly explained by variation in mean annual temperature (MAT; hypothesis H1), as mean annual precipitation (MAP) is generally high (1000‒3000 mm)^25,26^ and, thus, less important for mycorrhizal nutrient acquisition and stress alleviation. We then test whether AM tree dominance is related to the soil nutrient status, hypothesizing a negative relationship to the ratio of top soil C and N stocks (H2), in line with accelerated N mineralization under AM trees^3,13^. Finally, we estimate the AM tree dominance change between the first (1999–2004; T1) and the most recent NFI campaign (2015‒2019; T4), hypothesizing a significant increase in AM tree dominance (H3), promoted by an increase in MAT, N deposition, and the exposure to PWD and oak wilt disease (OWD), two diseases that have caused mass mortalities of EM trees in Japan^20,27,28^ (H4). When testing these hypotheses, we account for variation by ecoregion^15^ and effects by other potential predictors.

Here, we show that AM tree dominance has increased significantly between T1 and T4 in both natural (+2.5%) and planted forests (+1.3%). AM tree dominance increases are associated with the exposure to PWD and OWD, stressing the importance of forest pest and disease control to decelerate tree symbiosis shifts. A negative relationship between AM tree dominance and the ratio of soil C and N stocks in NFI campaign T2 suggests that tree symbiosis shifts along the Japanese archipelago will affect soil N availability and retention.

## Results

### AM tree dominance across the study area

**Fig. 1:**
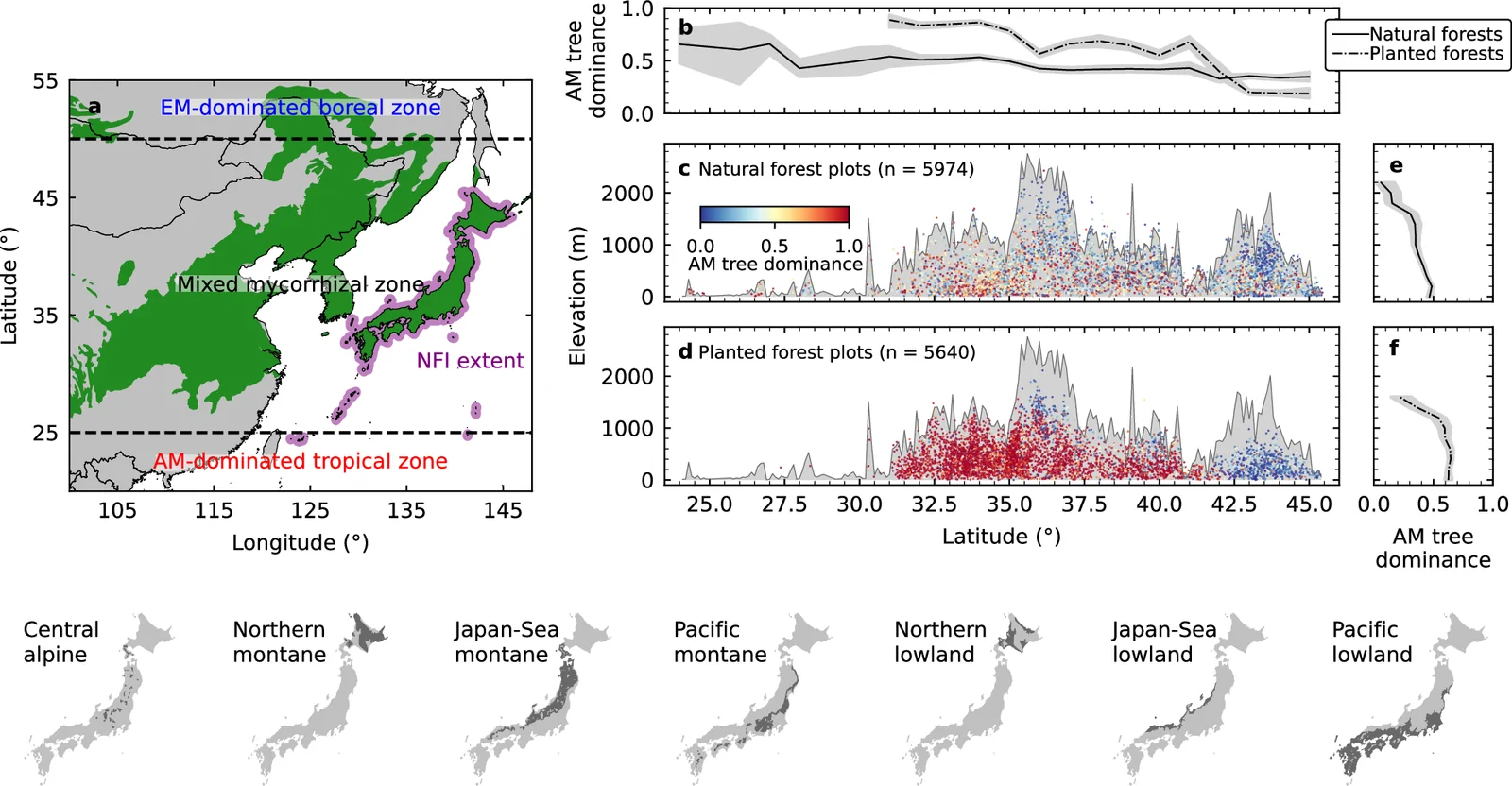
Arbuscular mycorrhizal (AM) tree dominance distribution in campaign T4 (2014–2019) of the Japanese National Forest Inventory (NFI). **a** The extent of the NFI in the context of East Asian temperate forest biomes^66,69^ (green) and the mycorrhizal zones proposed by Steidinger et al.^9^. Country borders were drawn based on Runfola et al.^95^, forest biomes based on data from RESOLVE^68^. **b** Mean AM tree dominance and 95% confidence intervals for each degree of latitude (−0.5 to +0.49°). **c**,** d** AM tree dominance in natural and planted forest plots by latitude and elevation. **e**,** f** Mean AM tree dominance and 95% confidence interval for every 200 m elevation (−100 to +99 m). Temperate ecoregions of Japan after Olson et al.^66,67,69^ are indicated in dark gray at the bottom of the figure. Ecoregions were drawn based on data from RESOLVE^68^. Source data are provided as a Source data file.

The distribution of approximately 700,000 living stems of over 500 tree species across 21◦ of latitude was analyzed (NFI campaign T4; 2014–2019; Fig. 1a–f). AM tree dominance in natural forest plots gradually decreased towards higher latitudes, with mean values of 66% (*n* = 12 plots, 95% confidence interval (CI) = 48–80%) and 35% (*n* = 149 plots, 95% CI = 31–39%) at 24 and 45°N, respectively (Fig. 1b). Along the elevation gradient, AM tree dominance decreased from 47% (*n* = 729 plots, 95% CI = 45–49%) to 6% (*n* = 6 plots, 95% CI = 2–17%) between 0 and 2200 m (Fig. 1e). In planted forest plots, the use of economic conifer species of the AM type (mostly conifers *Chamaecyparis obtusa* and *Cryptomeria japonica*) and EM type (mostly conifer of the genera *Abies*, *Larix*, *Picea*, and *Pinus*) varied distinctly by latitude and elevation, resulting in abrupt shifts of 20% at 35–36°N and 40% at 41–43°N and overall stronger gradients compared to natural forest plots (Fig. 1b, f). Since AM and EM trees comprised ≥95% of the basal area in 97% of forest plots, distributions of the AM and EM tree dominance were largely opposite (Supplementary Figs. 1 and 2). Other mycorrhizal types comprised only a small proportion of basal area across the analyzed forest plots (Supplementary Figs. 3 and 4).

### AM tree dominance in relation to climate and soil

**Fig. 2:**
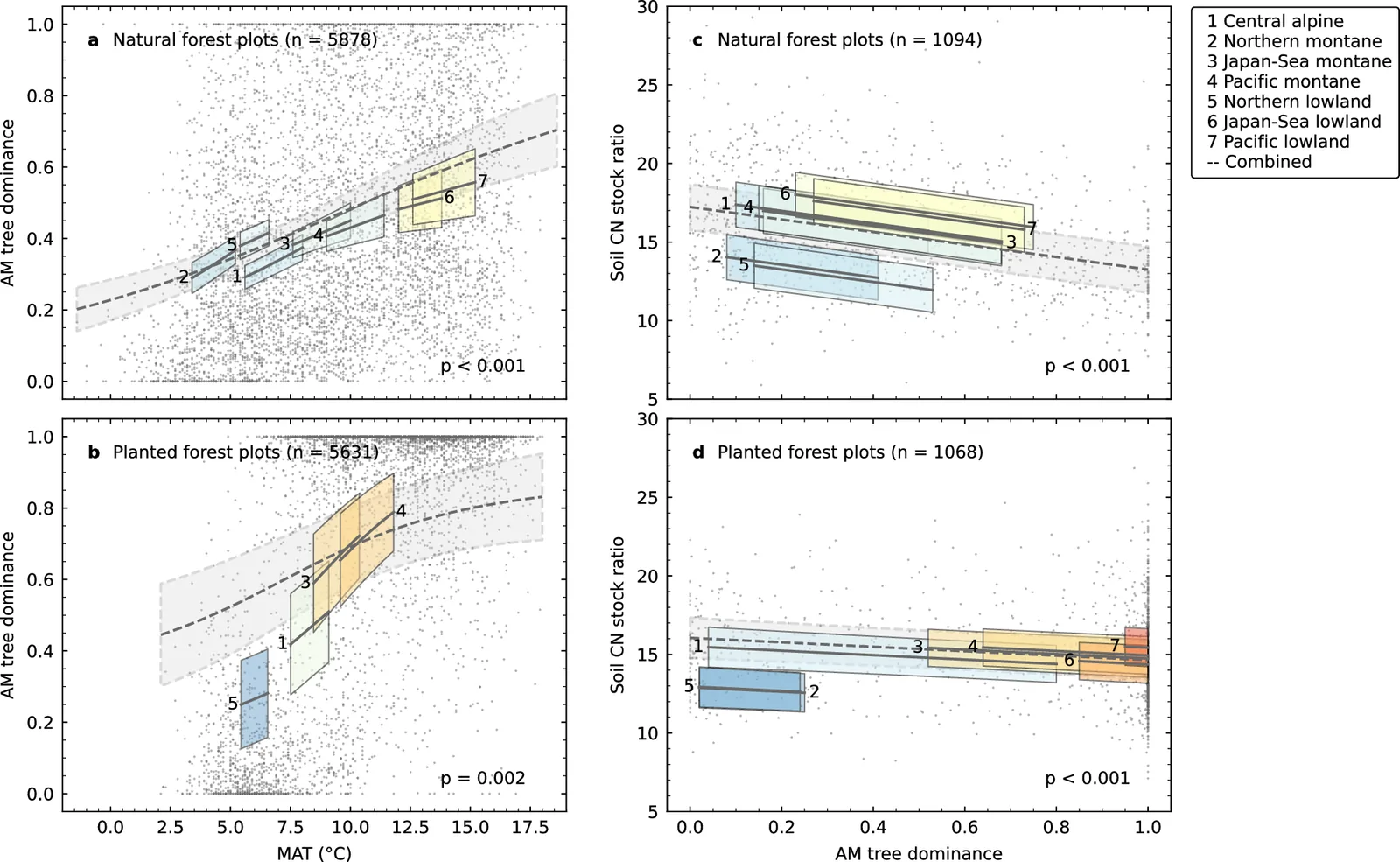
Relationships of arbuscular mycorrhizal (AM) tree dominance to mean annual temperature (MAT) and the ratio of soil C and N stocks. **a**,** b** Marginal effect predictions of MAT on the AM tree dominance in natural and planted forest plots after accounting for effects of basal area, mean annual precipitation (MAP), and temperature and precipitation seasonality in generalized linear models (GLMs) 1–2. **c**,** d** Marginal effect predictions of AM tree dominance on the ratio of soil C and N stocks in natural and planted forest plots after accounting for effects of basal area, species richness, tephra deposition, slope inclination, MAT, and MAP in GLMs 3–4. Marginal effect predictions were computed from GLMs with standardized predictors. Exact *p* values for predictor effects are 2.04 × 10^−8^ (**a**), 8.99 × 10^−30^ (**c**), and 1.63 × 10^−4^ (**d**; Wald Z-tests, two-sided; Supplementary Data 1). Variation in the effect of the main predictor by ecoregion was assessed by comparing models with and without random slopes (χ^2^-test, one-sided; Supplementary Table 1). GLMs 1–2 have random slopes and intercepts by ecoregion, GLMs 3–4 random intercepts by ecoregion. Ecoregion-specific MAT effect predictions are only shown for ecoregions with significant average marginal effects (Wald Z-tests, two-sided; Supplementary Table 2). Lines are drawn between the 25th and 75th percentile of the predictor data in each ecoregion. Shades represent 95% confidence intervals. Shade colors correspond to the mean AM tree dominance in the analyzed plots of each ecoregion (color legend in Fig. 1c). Marginal effect predictions for other predictors are shown in Supplementary Figs. 9–12. Source data are provided as a Source data file.

The relationship between AM tree dominance in NFI campaign T4 and MAT was assessed using generalized linear models (GLMs) with standardized predictors (GLMs 1 and 2; Supplementary Fig. 5; Supplementary Data 1). Consistent with hypothesis H1, MAT had a significant positive effect on AM tree dominance in both natural (z = 5.61, *i* = 2.04 × 10^−8^, effect size = 0.48, 95% CI = 0.31–0.65) and planted forest plots (z = 3.14, *p* = 0.002, effect size = 0.53, 95% CI = 0.20–0.87; Wald Z-tests, two-sided) that exceeded other significant effects in size (Supplementary Fig. 7a, b). However, the difference in effect size was smaller in planted forest plots (Supplementary Fig. 7a, b). The MAT effect varied significantly by ecoregion (natural: χ^2^(2) = 17.22, *p* = 1.82 × 10^−4^; planted: χ^2^(2) = 185.12, *p* = 6.34 × 10^−41^; χ^2^-tests for random slopes, one-sided; Supplementary Table 1). In natural forest plots, AM tree dominance increased by 3.1–4.4% per 1 °C MAT in northern ecoregions but only by 1.7–1.9% per 1 °C MAT in the Japan-Sea and Pacific lowlands (average marginal effects; Fig. 2a, Supplementary Table 2). In planted forest plots, the effect of MAT was non-significant in ecoregions with extremely high or low AM tree dominance (Fig. 2a, Supplementary Table 2).

The hypothesized relationship between AM tree dominance and the ratio of top soil C and N stocks (H2) was assessed in NFI campaign T2 (2004–2009), when a soil inventory (0–30 cm soil depth) was conducted in every fifth NFI plot (GLMs 3–8; Supplementary Fig. 6; Supplementary Data 1). In both natural and planted forest plots, AM tree dominance had a significant negative effect on the ratio of soil C to N stocks (Fig. 2c, d; Supplementary Fig. 8a, b). Variation in the AM tree dominance effect by ecoregion in natural forest plots was not significant (χ^2^(2) = 0.33, *p* = 0.849; χ^2^-test for random slopes, one-sided; Supplementary Table 1). In planted forest plots, the model with different AM tree dominance effects by ecoregion did not converge, suggesting overparameterization (Supplementary Table 1). Although absolute soil C and N stocks were most affected by slope inclination and MAT, AM tree dominance had a significant negative effect on the soil C stock in natural forest plots (z = −1.99, *p* = 0.047, effect size = −0.179, 95% CI = −0.355– −0.003) and significant positive effects on the soil N stock in natural (z = 4.02, *p* = 5.94 × 10^−5^, effect size = 0.023, 95% CI = 0.012–0.035) and planted forest plots (z = 2.04, *p* = 0.041, effect size = 0.017, 95% CI = 0.001–0.033; Wald Z-tests, two-sided; Supplementary Fig. 8c–f).

### AM tree dominance change and its drivers

**Fig. 3:**
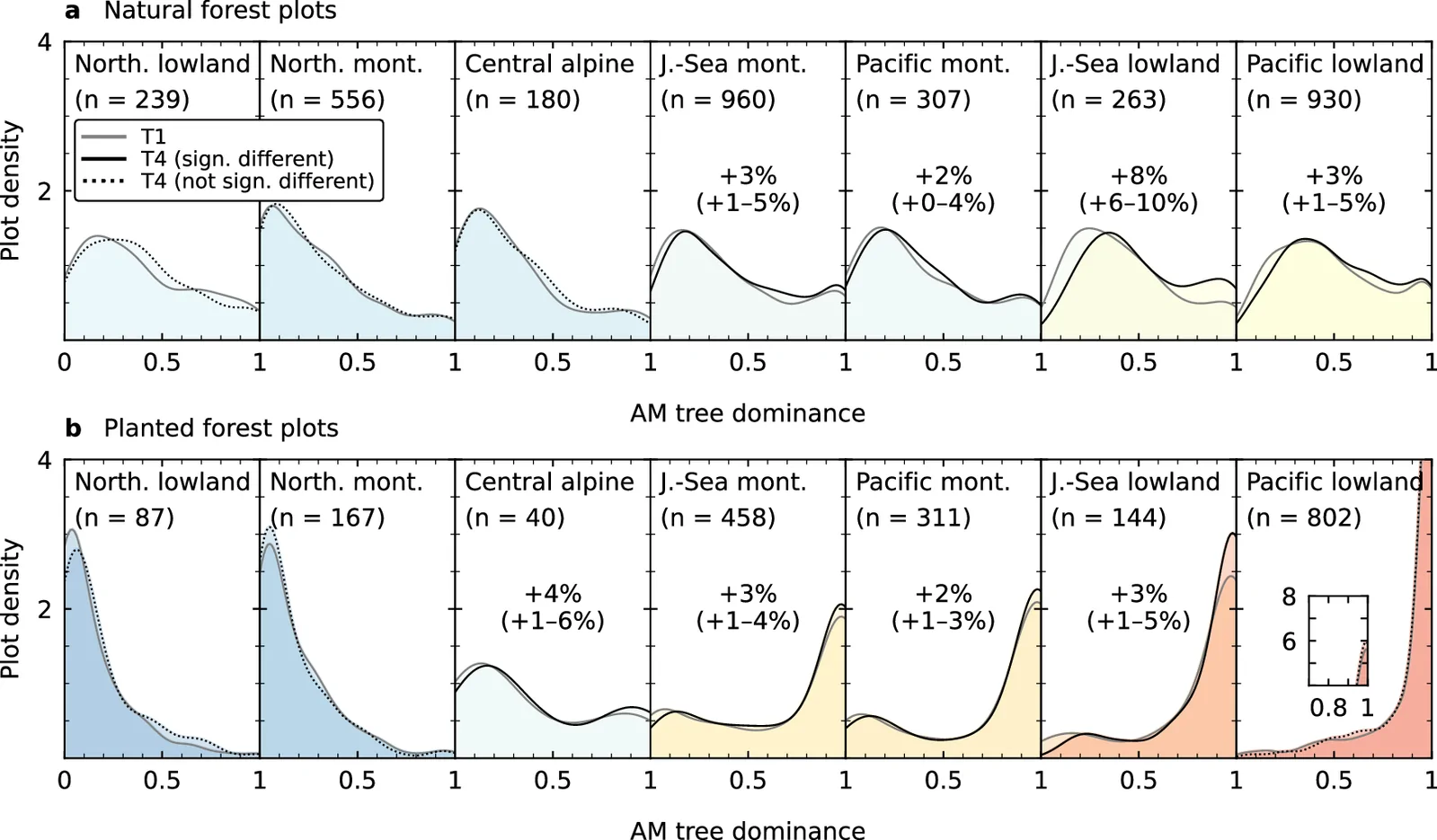
Change in the arbuscular mycorrhizal (AM) tree dominance distribution between inventory campaigns T1 (1999–2004) and T4 (2014–2019). **a** Plot density by AM tree dominance compared between campaigns T1 and T4 in natural forest plots. **b** Plot density by AM tree dominance compared between campaigns T1 and T4 in planted forest plots. Plot densities were calculated with kernel density estimation. Ecoregion-specific average marginal effects were computed from generalized linear models (GLMs) 9 and 10. Solid black lines indicate a significantly higher AM tree dominance in T4 compared to that in T1 (*p* < 0.05; Wald Z-test, two-sided; Supplementary Table 3). In significant cases, the increase in AM tree dominance (average marginal effect with 95% confidence interval) is shown as a percentage. The number of plots that were compared between inventory campaigns is indicated below each ecoregion name. The color under each curve corresponds to the mean AM tree dominance in the analyzed plots of each ecoregion (color legend in Fig. 1c). Source data are provided as a Source data file.

AM tree dominance increased significantly by 2.5% (z = 2.68, *p* = 0.007, 95% CI: 0.7–4.4%) and 1.3% (z = 2.33, *p* = 0.020, 95% CI: 0.2–2.4%; average marginal effects; Wald Z-tests, two-sided) in natural and planted forest plots, respectively, during the 15-year period between NFI campaigns T1 and T4 (GLMs 9–10; Supplementary Data 1), consistent with hypothesis H3. The campaign effect varied significantly by ecoregion (natural: χ^2^(3) = 49.98, *p* = 8.07 × 10^−11^; planted: χ^2^(3) = 42.00, *p* = 4.01 × 10^−9^; χ^2^-test for random slopes, one-sided; Supplementary Table 1), indicating ecoregion-specific increases of 2–8% (average marginal effects; Fig. 3a, b; Supplementary Table 3). All significant increases were associated with reductions in NFI plots with 0–20% AM tree dominance (kernel density estimates; Fig. 3a, b). In natural forest plots, AM tree dominance increases resulted from higher net basal area increases of AM and compared to EM trees (Supplementary Figs. 17a, b and 23a, c). In planted forest plots, basal area increases were largely reflective of the stem growth of the dominant economic tree species and, hence, less conclusive (Supplementary Figs. 18a, b and 23b, d).

**Fig. 4:**
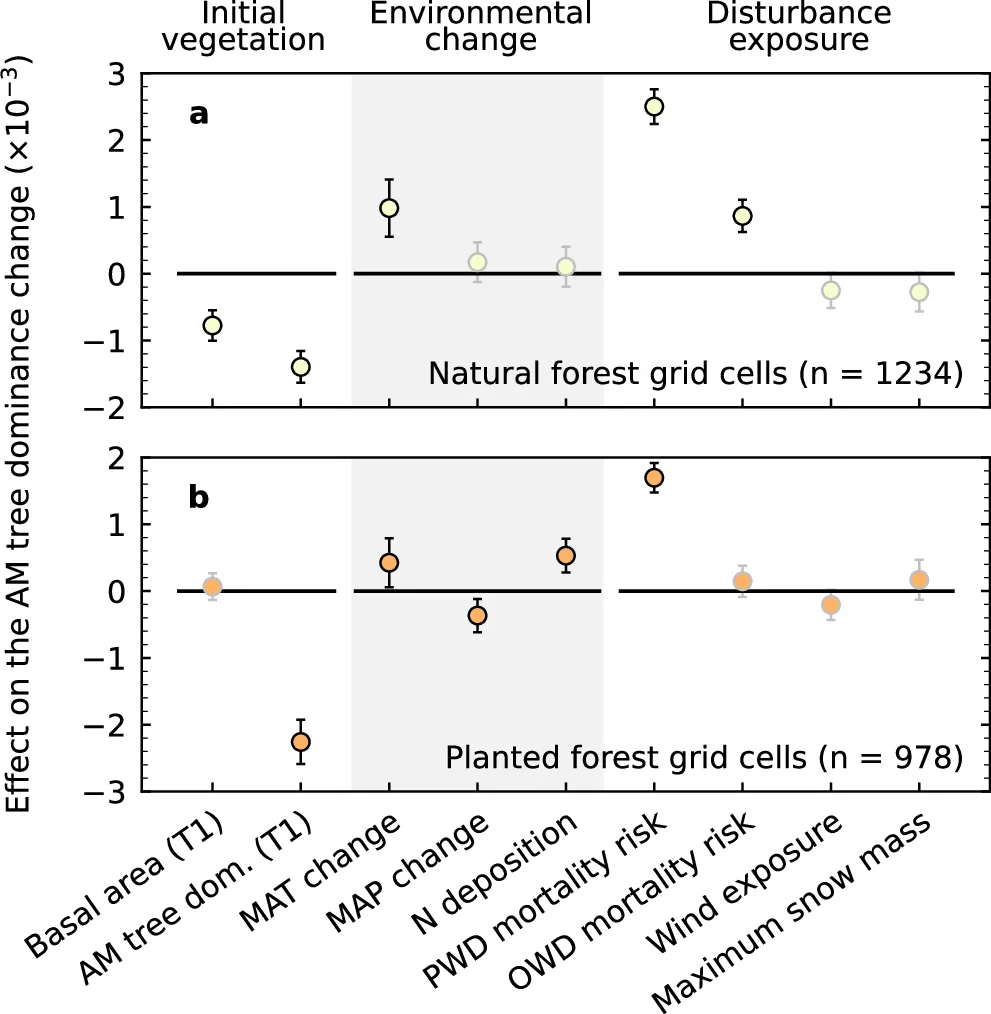
Predictors of the arbuscular mycorrhizal (AM) tree dominance change between inventory campaigns T1 (1999–2004) and T4 (2014–2019). **a** Comparison of predictor effect sizes for the AM tree dominance change in natural forests in generalized linear model (GLM) 11. **b** Comparison of predictor effect sizes for the AM tree dominance change in planted forests in GLM 12. MAT, MAP, PWD, and OWD stand for mean annual temperature, mean annual precipitation, pine wilt disease, and oak wilt disease, respectively. Analysis was conducted with grid-based data (15-km resolution). Predictor effect sizes were computed from GLMs with standardized predictors. Error bars indicate 95% confidence intervals. Dot colors correspond to the mean AM tree dominance of the analyzed grid cells in T4 (color legend in Fig. 1). Dots with black borders indicate significant effects (*p* < 0.05; Wald Z-test, two-sided; Supplementary Data 1). *P* values from left to right (**a**): 2.67 × 10^−11^, 1.53 × 10^−30^, 7.25 × 10^−6^, 0.257, 0.493, 1.49 × 10^−79^, 1.90 × 10^−12^, 0.063, and 0.064. *P* values from left to right (**b**): 0.516, 1.04 × 10^−40^, 0.024, 0.004, 3.63 × 10^−5^, 5.24 × 10^−51^, 0.219, 0.074, and 0.265. Marginal effect predictions with underlying data distributions of all predictors are shown in Fig. 5 and Supplementary Figs. 24 and 25. Source data are provided as a Source data file.

**Fig. 5:**
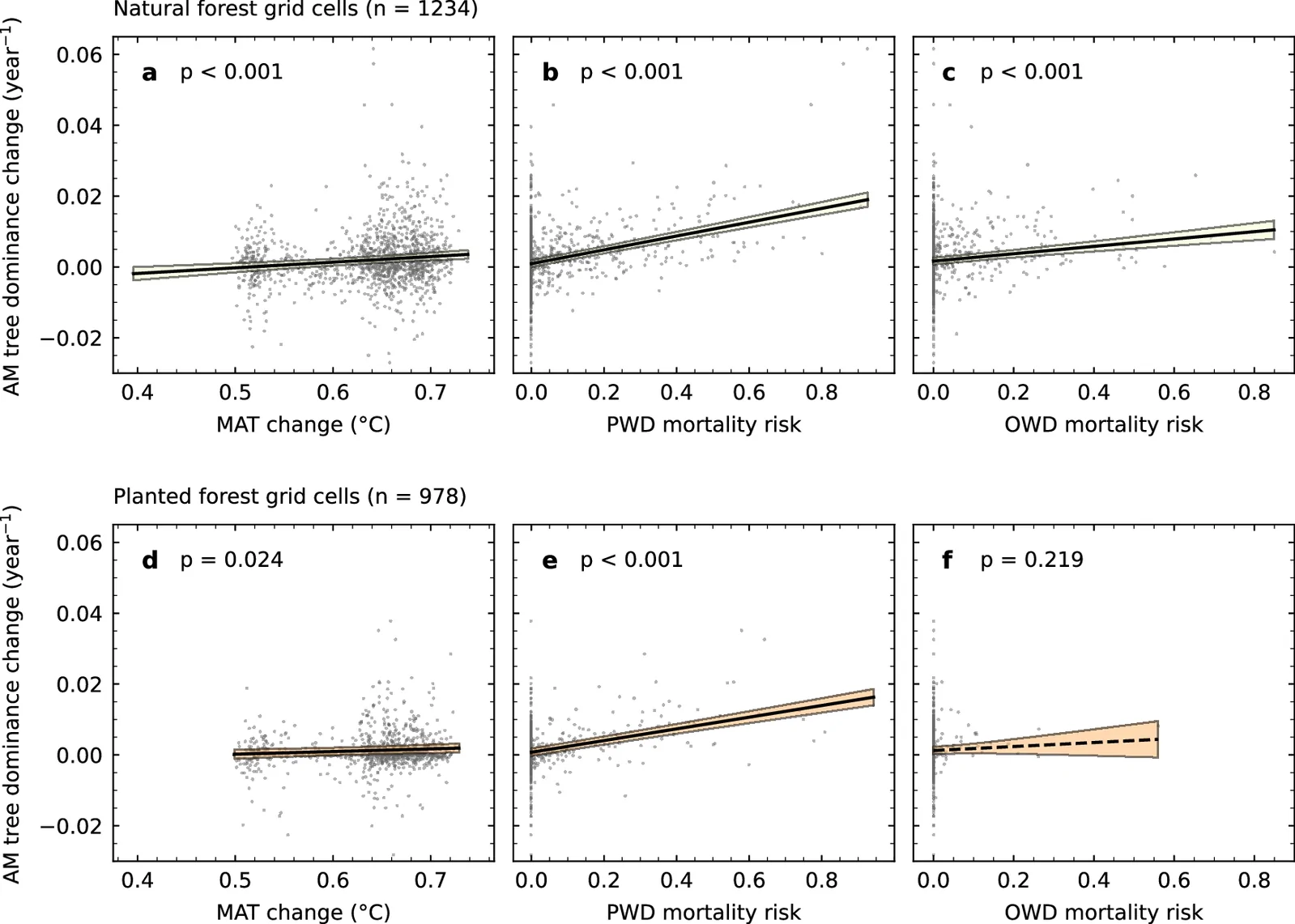
Relationships of the arbuscular mycorrhizal (AM) tree dominance change to climate warming and tree disease mortality risks. **a**–**c** Marginal effect predictions for natural forest grid cells in generalized linear model (GLM) 11. **d**–**f** Marginal effect predictions for planted forest grid cells in GLM 12. MAT, PWD, and OWD stand for mean annual temperature, pine wilt disease, and oak wilt disease, respectively. Marginal effect predictions were computed from GLMs with standardized predictors. Solid lines indicate significant predictor effects (*p* < 0.05; Wald Z-test, two-sided; Supplementary Data 1). Exact *p* values for predictor effects are 7.25 × 10^−6^ (**a**), 1.49 × 10^−79^ (**b**), 1.90 × 10^−12^ (**c**), and 5.24 × 10^−51^ (**e**). Shades represent 95% confidence intervals. Shade colors correspond to the mean AM tree dominance of the analyzed grid cells in T4 (color legend in Fig. 1c). All predictors accounted for in marginal effect predictions are shown in Fig. 4. Marginal effect predictions of other predictors are shown in Supplementary Figs. 24–25. Source data are provided as a Source data file.

To identify the drivers of AM tree dominance changes, effect sizes of standardized predictors were compared (GLMs 11–12; Supplementary Data 1). For this, plot data was interpolated to a 15-km-resolution grid, the lowest resolution among the tested predictors (Supplementary Figs. 21 and 22). Consistent with hypothesis H4, MAT change and PWD and OWD exposure—here estimated as disease-related mortality risks—had significant positive effects (Figs. 4 and 5). PWD mortality risk represented the largest significant positive effect in both natural and planted forest grids, whereas OWD mortality was similar in effect size to MAT change in the natural forest grid (Fig. 4a, b). The effect of N deposition was significant in the planted forest grid but small (Fig. 4b). The relative effect sizes changed little when the mortality risk of the less disease-affected oak species *Quercus serrata* was modified (Supplementary Fig. 26). To confirm the effects of PWD and OWD exposure, AM tree dominance changes between T1 and T4 were estimated separately for infected and non-infected forest plots (GLMs 13–14; Supplementary Data 1). In non-infected natural and planted forest plots, AM tree dominance increased by only 0.6% (z = 2.59, *p* = 0.010, 95% CI: 0.2–1.1%) and 0.4% (z = 2.10, *p* = 0.036, 95% CI: 0.03–0.7%; average marginal effects; Wald Z-test, two-sided), respectively. In infected plots natural and planted forest plots, AM tree dominance increased by 9.2% (z = 15.62, *p* = 4.35 × 10^−54^, 95% CI: 8.0–10.4%) and 9.0% (z = 8.25, *p* = 2.04 × 10^−16^, 95% CI: 6.8–11.1%; average marginal effects; Wald Z-test, two-sided), respectively.

### Potentially incorrect mycorrhizal type assignments

The sensitivity of our primary study results to potentially incorrect mycorrhizal type assignments was tested by recomputing GLMs 1–4 and 9–12 after reassigning the mycorrhizal types of tree species with contradictory reports (*n* = 37; Supplementary Data 2). Reassignments were made for randomly selected subsets of tree species in 100 iterations (Supplementary Figs. 28–34). After reassigning mycorrhizal types, negative effects of wind exposure and maximum snow mass on the AM tree dominance change in natural forest plots became significant in >70 iterations (Wald Z-tests, two-sided; GLM 11; Supplementary Figs. 33h, i). Effect sizes were, however, small compared to other predictors. Furthermore, the effect of MAT change on the AM tree dominance change in planted forest plots became insignificant in 66 iterations (Wald Z-tests, two-sided; GLM 12; Supplementary Fig. 34d). In all 66 iterations, EM species of the genus *Castanopsis* were reassigned the mycorrhizal type AM-EM. Other results were little affected by the reassignments (Supplementary Figs. 28–34).

## Discussion

Temporal distribution shifts of mycorrhizal tree symbioses were observed in both natural and planted temperate forests, with AM tree dominance increasing significantly within 15 years. Ecoregion-specific increases in AM tree dominance were 2–8% within 15 years (Fig. 3a, b) and, hence, similar to those in North America where AM tree dominance increased by 5–17% within 30 years^15^. However, the apparent drivers of the AM tree dominance change differed. The comparatively large positive effects of PWD and OWD mortality risks indicate that tree diseases were the main drivers of the AM tree dominance increase, outweighing the positive effect of climate warming (Figs. 4 and 5). Effects of N deposition, MAP change, and exposure to abiotic disturbance—important drivers in North America^15^—were small or insignificant. While our results do not allow us to conclude why the effect of N deposition was small, a study of ecosystem N saturation in East and Southeast Asia indicated comparatively high leaching of deposited N in Japan^19^. Similar findings were presented in a regional study of stream water nitrate concentrations^29^. Thus, much of the deposited N may be leached out of the top soil under high precipitation, limiting N accumulation and its effect on mycorrhizal nutrient uptake. Hence, anthropogenic N deposition is likely less influential on tree symbiosis shifts in the precipitation-rich temperate forests of Japan compared to drier temperate forests in North America.

Significant effects of PWD and OWD are in line with the severe impact of tree diseases and pests on the composition, structure, and function of forest ecosystems^23,30,31^. More so than abiotic forest disturbance, tree diseases and pests tend to selectively remove tree species and genera^30,32,33^. Similar to OWD and PWD, which cause mortality of common *Quercus* and *Pinus* species, respectively^28,34^, many major biotic disturbance agents in Europe, the United States, and China target EM tree species, specifically, members of the Pinaceae family^23^. While the removal of EM trees does not necessarily result in the spread of AM trees, environmental changes such as climate warming benefit AM more than EM tree species^9,15^ and make a replacement with AM trees more likely. Indeed, our results show distinct increases in the dominance and net biomass of AM trees in natural forest plots that were largely EM tree-dominated in T1 (Fig. 3a; Supplementary Fig. 17a). Such a tree disease-induced increase in AM tree dominance was shown to have large long-term effects on forest C and N cycling on a regional scale, including increased N leaching^35^. Our findings show that tree diseases-induced tree symbiosis shifts may occur on a much broader scale, impacting the structure and nutrient cycling of temperate forests across many degrees of latitude.

The significant effect of climate warming on the AM tree dominance change is consistent with the finding that climatic controls of SOM decomposition are the primary drivers of broad-scale tree symbiosis distributions^9^. In line with the tendency of AM trees to thrive in decomposition-enhancing, warm and wet climates, AM tree dominance along the Japanese archipelago was significantly positively affected by MAT and MAP (Fig. 2a, b; Supplementary Fig. 7a, b). The larger effect size of MAT compared to MAP was expected because, in precipitation-rich climates, soil moisture likely does not limit SOM mineralization. Similarly, mycorrhizal alleviation of water stress^5^, which could increase the apparent influence of MAP, likely contributed little in this study because drought has been limited^36^. Conversely, mycorrhizal alleviation of temperature-induced stress—in the form of, e.g., lowered photosynthetic activity, disrupted phenology, or inhibited off-spring development^37^—may explain some of the MAT effect. Notably, the MAT effect was strongest in natural forests of cooler ecoregions (Fig. 2a, Supplementary Table 2), in line with the increased temperature sensitivity attributed to boreal tree species near their distribution edges in cool temperate areas^5^. Thus, our findings overall confirm the close climate-coupling of tree symbiosis distributions shown in previous research^9,15^.

Yet, unlike the gradual latitudinal transition of AM tree dominance in natural forests, AM tree dominance changed abruptly at 35–36°N and 41–43°N in planted forests (Fig. 1b). Most studies on tree symbiosis distributions have not distinguished between planted and natural forests. However, the tree symbiosis abundance in planted forests is likely less responsive to climatic gradients, as tree species compositions are influenced by management decisions related to productivity and conservation. Indeed, effects of MAT on AM tree dominance and MAT change on the AM tree dominance change were less pronounced in planted compared to natural forests (Fig. 4a, b, Supplementary Fig. 7a, b), illustrating the smaller influence of climate and its control of SOM decomposition in planted forests. Particularly in the temperate forest biome, where the influence of human land use^38^ and planted forest proportions are high^18^, separation of natural and planted forests may, therefore, enable more accurate assessment of the climate feedbacks of broad-scale tree symbiosis distributions.

Forest nutrient cycling was closely linked to the dominating mycorrhizal symbiosis type. The ratio of top soil C and N stocks significantly decreased with AM tree dominance (Fig. 2c, d), similar to temperate forests in North America^15,39^. In natural forests, this decrease was associated with higher N stocks as well as lower C stocks (Supplementary Fig. 8c, e). However, absolute C and N stocks were more strongly affected by MAT and slope inclination, key abiotic determinants of forest SOM decomposition and soil erosion, respectively, in the local climate^40,41^. More distinct was the effect of AM tree dominance on soil C and N stock ratios (Supplementary Fig. 8a, b). High availability of N per unit C in soil indicates reduced N demand by saprotrophic soil microbes and increased potential for nitrification and subsequent N leaching^15,42,43^, linking N availability to N retention. While the relationship of AM tree dominance to N availability is partially explained by a competitive advantage of AM trees in N-rich soil^15,39^, extensive evidence of mycorrhizal effects on, e.g., microbial community functions^44^, soil biochemistry^45^, and leaf litter quality^13^ suggests that AM trees actively promote the soil N mineralization and availability in temperate forests. The relationship of AM tree dominance to the ratio of soil C and N stocks, hence, indicates that the AM tree dominance shift along the Japanese archipelago will decrease N retention. Nevertheless, data on soil C and N stocks in our study were limited to NFI campaign T2. To strongly support a link between AM tree dominance and N retention, follow-up studies are needed that analyze time series data.

Comparison of the estimated AM tree dominance across all NFI plots in temperate ecoregions in T4 (aggregated to a 1°-resolution grid of means^9^; beta distribution assumed) to predictions made for Japan in a recently published global tree symbiosis map^9^ shows that the observed AM tree dominance was significantly higher than predicted (z = 9.55, *p* = 2.54 × 10^−16^, average marginal effect = 18%, 95% CI = 14–21%; Wald Z-test, two-sided; *n* = 62; GLM 15; Supplementary Data 1, Supplementary Fig. 27). This difference may be partially explained by the environmental change and disturbance exposure over time, as our estimates are based on more recent tree inventory data^9,46^. However, the higher AM tree dominance also exceeded the AM tree dominance projected for most of Japan by 2070^9^, suggesting that previous AM tree dominance predictions were underestimated, likely due to the limited data coverage across Japan^9^. Our analysis of extensive tree inventory data, hence, indicates that AM symbiosis currently plays a more important role in forest ecosystems along the Japanese archipelago than previously expected.

The sensitivity of our primary study results to potentially incorrect mycorrhizal type assignments was generally low (Supplementary Figs. 28–34). Notably, however, the effect of MAT change on the AM tree dominance change in planted forests became usually insignificant when the EM oak species *Castanopsis sieboldii* and *Castanopsis cuspidata* were reassigned AM-EM (Supplementary Fig. 34d). The two species are dominant in natural forests of warmer ecoregions and regenerate naturally in nearby planted forests (Supplementary Fig. 35). AM-EM assignments of *C. sieboldii* and *C. cuspidata* originated from the FungalRoot database^47^, which references a pioneering mycorrhizal survey of Japanese plant species by Maeda (1954) but cautions that AM colonization is unimportant in adult trees^48^. Indeed, both *C. sieboldii* and *C. cuspidata* are considered to primarily host EM fungi in Japanese forests^49–51^, suggesting that the reported study results are not invalidated by the outcome of the sensitivity analysis. Nevertheless, the presence of reassignment effects underpins calls to carefully consider and verify mycorrhizal type assignments in ecological meta-analyses^52^.

In conclusion, our findings indicate that tree diseases drive a broad-scale tree symbiosis shift along the Japanese archipelago, contributing to a more important role of AM symbiosis in forest ecosystems than previously predicted. The observed relationship between AM tree dominance and soil nutrient status in NFI campaign T2 suggests that tree symbiosis shifts will alter forest N cycling, posing environmental risks such as increased N leaching to surface waters. Owing to international trade and climate change, forest disturbance from tree diseases and pests is intensifying globally^53–55^. PWD has recently spread to Europe and is expected to cause severe disturbance to local forests based on models of the local climate, disease spread, and host distribution^20,56^. Thus, distribution shifts of tree symbioses may occur in a wide range of forest ecosystems, despite restrictions on anthropogenic N pollution. Yet, data collection on tree diseases and pests remains severely limited, with even rudimentary annual data on disease-affected areas being available for <40% of global forests^18^. Our results should promote follow-up studies on how tree diseases link to tree symbiosis distributions, particularly in dry temperate forests of continental China, where N deposition and tree diseases may both be important factors of tree symbiosis distribution changes, as deposited N accumulates more easily^19^. Moreover, our findings highlight the importance of developing and enforcing strategies for pest and disease control in forests–including extensive monitoring of disease spread, early removal of disease-affected trees, and the creation of disease-resilient forest ecosystems^57^–to decelerate tree symbiosis shifts and their impact on ecosystem functioning.

## Methods

### Tree inventory data

The diameter at breast height (DBH; cm), Japanese species name, and living status (dead versus alive) of individual trees in approximately 13,900 forest plots were obtained from the NFI dataset of the Japanese Forestry Agency^58^. NFI plots were set up on the intersections of a 4 × 4 km square grid. Each plot was comprised of an inner (0.01 ha), middle (0.03 ha), and outer section (0.06 ha), where stems with a minimum DBH of 1, 5, and 18 cm were recorded, respectively. Data was available for four consecutive 5-year campaigns from 1999 to 2019 (T1–T4). For this study, inventory data of stems with a DBH ≥ 5 cm in the inner and middle plot sections were analyzed. Additionally, plot location coordinates (rounded to 0.01°), elevations (rounded to 10 m), investigation dates, stand ages, cutting histories, location changes, occurrence of disturbance (type, observed/not observed), and forest regeneration types were obtained from the NFI dataset. Further information on the NFI dataset has been provided elsewhere^59,60^.

### Species translation and mycorrhizal type assignment

Japanese species names (1231 unique entries) were corrected for obvious misspellings, replaced with more common name alternatives, and cleared of unnecessary punctuation and characters. Furthermore, entries that failed to identify plants at the genus or species level and entries that referred to herbaceous plants or dwarf bamboo (genera *Sasa*, *Sasaella*, *Pseudosasa*, *Pleioblastus*), which commonly have a DBH < 5 cm, were excluded. Among the remaining 931 entries, 927 were successfully translated into standard scientific names using extensive plant name indices^61,62^, corresponding to 740 unique plant species and 28 unique plant genera. Thick-stemmed bamboo species as well as woody fern, vine, and palm species were considered trees, as in previous studies^9^. Corresponding mycorrhizal types were assigned using databases of species-level^9^ (307 cases) and genus-level assignments^63^ (461 cases). Genus-level assignments were based on ≥67% consistency of mycorrhizal type diagnoses in the FungalRoot database^63^ which has since been updated to version 2.0^47^. Among the translated species, 581 were AM, 108 were EM, 45 were ericoid mycorrhizal, 17 were AM-EM, and none were non-mycorrhizal (NM). Furthermore, 17 were weakly or facultatively AM (AM-NM) species, which belong to genera with inconsistent or low records of AM fungal colonization^9,63^, whose capacity to form mycorrhiza may depend on, e.g., habitat conditions^2,52^.

Incorrect mycorrhizal type assignments may bias the conclusions of ecological meta-analyses^52^. Assignments at the genus-level, for example, while consistent with the strong phylogenetic conservatism of mycorrhizal types^2^, may ignore mycorrhizal type variation within genera due to habitat or nutritional specialization^2,63^. To assess the extent of potentially incorrect mycorrhizal type assignments in our study, we compared species-level assignments^9^, genus-level assignments^63^, and species-level diagnoses in FungalRoot^47^ to identify contradictory reports. For this, we extracted all diagnoses of NFI tree species from the FungalRoot database (retrieved via PlutoF^64^ on April 27, 2026; 1665 entries) and removed diagnoses where AM colonization was undetermined (586 entries), diagnoses marked as incorrect report, probably incorrect, and misleading (194 entries), and diagnoses of the type Other (2 entries). In total, 37 NFI tree species had contradictory reports (Supplementary Data 2), which were used to test the sensitivity of our study results to potentially incorrect mycorrhizal type assignments (see the Statistical analysis section).

### Plot categorization

NFI plots were separated into natural (i.e., naturally regenerated or sprouted plots) and planted plots, which differed in basal area, stand age, tree species composition, and species richness (i.e., the number of tree species in a plot^65^; Supplementary Table 7). Furthermore, plots were assigned ecoregions^66–69^ (Fig. 1; Supplementary Table 5). Ecoregions were mainly defined by latitudinal and elevational differences in temperature^67^ (Supplementary Table 6). While precipitation was generally high (1200–2200 mm by ecoregion; Supplementary Table 6), stronger influences of winter and summer monsoons in Japan-Sea and Pacific ecoregions, respectively, result in different seasonal precipitation patterns, i.e., higher snowfall in Japan-Sea ecoregions^25,67^. In planted forests, AM conifer species *C. obtusa* and *C. japonica* were dominant in Japan-Sea and Pacific ecoregions, while EM conifer species (in particular *Larix kaempferi* and *Abies sachalinensis*) were dominant in the cooler northern and alpine ecoregions (Supplementary Table 7). Data from plots designated as harvested plantations or bare land, plots with undefined or missing regeneration types, plots younger than 5 years in T1, and data from investigations with erroneous dates were excluded from statistical analyses. Furthermore, a single DBH entry of >300 cm in T4 that was not recorded in T1–T3 was removed due to assumed error in reporting. Finally, the small number of plots (*n* = 99) assigned to subtropical ecoregions were excluded from statistical analyses. For comparisons of NFI data between campaigns, only plots that were measured repeatedly with valid investigation dates and recorded as neither cut nor relocated were considered. Plots were considered cut based on the on-site observations (traces of felling or stump removal, cut branches, pruning or cutting scars, replanting, and artificial seeding) and cutting histories recorded in the NFI dataset.

### Calculation of basal area and AM tree dominance

The basal area (m^2^) of each plot and campaign was calculated as the sum of the basal areas of all living tree stems. Stems of unidentified species accounted for <0.1% of the basal area across all plots in T4. The dominance of AM, EM, ericoid, and non-mycorrhizal trees was calculated as the basal area proportion of the tree species of the respective mycorrhizal type^15^. Dominance values, which were between 0 and 1, were expressed as percentages in the main text to improve readability. For tree species that were assigned two mycorrhizal types (e.g., AM-EM, AM-NM), only half of their basal area was included, similar to previous studies^15,24^. The change in AM tree dominance was calculated by dividing the difference between the T1 and T4 estimates by the time between the campaigns (approximately 15 years).

### Soil data

Soil C stock, N stock, and their ratio (0–30 cm soil depth) were calculated from the National Forest Soil Carbon Inventory (NFSCI) conducted in 2379 NFI plots from 2006 to 2010, in parallel to NFI campaign T2 (2004–2009). NFSCI plots were evenly distributed among the full set of NFI plots (Supplementary Fig. 36) and had similar climate and basal area distributions (Supplementary Fig. 37), suggesting little influence by the plot reduction on the representativeness of observed soil-climate-vegetation relationships. Sampling and sample analysis of the NFSCI have been described elsewhere^40,41,70^. Briefly, four 30 cm-deep soil profiles were excavated at each plot. Then, C and N concentrations in the mineral soil (CONC; mg g^−1^), apparent coarse rock fractions (RF; %), and bulk densities of the mineral soil (BD; g cm^−3^) in soil layers at 0–5, 5–15, and 15–30 cm depths were compiled for all four sampling locations per plot. RF was estimated by visually assessing the area of rock fragments in the soil layers. BD was estimated from the mass of 400-cm^3^ cylindrical cores or a cuboid clods after oven-drying (105 °C, 24 h) and sieving (2 mm mesh size). C and N concentrations were estimated by dry combustion of air-dried and sieved (2 mm mesh size) soil samples. At each sampling location, the masses of C and N (MASS; kg m^−2^) in each soil layer with height H (m) were then calculated as: ${{\rm{MASS}}}={{\rm{CONC}}}\times {{\rm{BD}}}\times (1-{{\rm{RF}}}/100)\times {{\rm{H}}}$. Finally, soil C and N stocks in each plot were calculated by combining the soil layer C and N masses for each sampling location and calculating the average across the four sampling locations.

Slope inclinations across Japan were computed from the digital elevation model of Japan’s Geospatial Information Authority^71^ using the software SAGA (version 9.5.1). The deposition of tephra (cm), fragmental material ejected during volcanic eruptions, across Japan was obtained from a dataset compiled from tephra deposition maps for significant volcanic events within the last 100,000 years^72,73^. The exact plot coordinates available in the NFSCI dataset were used to extract the slope inclinations and tephra depositions of plots.

### Climate and N deposition data

MAT and MAP were obtained from data of averaged climate variables for 1991‒2020 collected by the Japan Meteorological Agency^74^. Using the same data, temperature seasonality was calculated as the standard deviation of monthly mean temperatures^9^ and precipitation seasonality was calculated as the coefficient of variation of monthly precipitation sums^9^. MAT and MAP changes were calculated as the difference between average monthly climate variables for 2000‒2019 (current climate) and 1980–1999 (preceding climate) in the TerraClimate dataset (version 1.0)^75^, which uses multi-source data and climatologically-aided interpolation to generate consistent time-series data. N deposition (kg N ha^−1^ year^−1^) was calculated as the average annual wet and dry deposition of N between 2000 and 2020 in a dataset simulated using a Community Multiscale Air Quality (CMAQ) model coupled with a Weather Research and Forecasting (WRF) model^76^.

### Exposure to biotic disturbance

OWD and PWD mortality risks in the NFI plots were calculated as the basal area proportions in T1 of tree species with high disease mortality. OWD in Japanese temperate forests—which is caused by the pathogenic fungus *Raffaelea quercivora* and vectored by the wood-boring ambrosia beetle *Platypus quercivorus*^27^—attacks a wide range of tree species of the Fagaceae family; however, only *Quercus crispula* and *Q. serrata* show high mortality^34^. PWD in Japanese temperate forests—which is caused by the pinewood nematode *Bursaphelenchus xylophilus* and vectored by pine sawyer beetles (*Monochamus spp*.)^20^—leads to high mortality of *Pinus densiflora* and *Pinus thunbergii*^28^. While broad-scale data on tree species-specific mortality rates are lacking, regional studies have reported lower cumulative mortality during disease outbreaks for *Q. serrata* (20–30%) than for *Q. crispula*, *P. densiflora*, and *P. thunbergii* (70–100%)^34,77–80^. To account for this difference, only half of the summed basal area of *Q. serrata* was added to the OWD mortality risk. Additionally, OWD mortality risks were estimated for proportions 0.3–0.7. When no disturbance by OWD or PWD was reported in a plot in any of the four campaigns, the disease mortality risk was assumed to be zero. However, it should be noted that under-reporting in apparently non-infected plots may have biased disease mortality effects toward the null in the statistical analysis. Disturbance caused by other tree diseases was less common in NFI plots and was not analyzed further (Supplementary Fig. 19).

### Exposure to abiotic disturbance

Wind exposure was calculated as a dimensionless wind effect index from wind speed, wind direction, and elevation data^81,82^ using the software SAGA (version 9.5.1; tool Wind Effect). Wind disturbance in forests results mostly from extreme winds^31^, in the specific case of Japan from typhoons^83^. Hence, the annual maximum wind speeds, obtained from hourly wind data in ERA5-Land^84^ and averaged for 2000–2019, were used for computation. Elevation data (≤ 300 km from the study area) were obtained from a global digital elevation/terrain model^85^. As most typhoons approach the archipelago from the south-west^86^, wind disturbance exposure was estimated for wind directions between 0 to 90° (5°-steps; direction into which the wind blows) and averaged. The maximum snow mass was calculated as the average annual maximum snow water equivalent (i.e., snow water mass per ground area) in daily data for 1999–2019 published by the National Agriculture and Food Research Organization of Japan^87^. The density distributions of wind and snow disturbance damage across NFI plots were significantly correlated with wind exposure (*ρ* = 0.38, *p* = 8.46 × 10^−71^) and maximum snow mass estimates, respectively (*ρ* = 0.72, *p* = 1.01 × 10^−239^; Spearman’s rank correlation, one-sided; Supplementary Fig. 20). See the Statistical analysis section for details. Other types of abiotic disturbance were less common in NFI plots and were not analyzed further (Supplementary Fig. 19).

### Data processing

Information on dataset formats is listed in Supplementary Table 4. Data were compiled using Python code (version 3.13.5) in Visual Studio Code (version 1.103.2). Tabular data were processed with the Python package pandas (version 2.3.1) and numpy (version 2.3.1). Vector data (format shapefile) were processed with the Python packages geopandas (version 1.1.1) and shapely (version 2.1.1). Data in GeoTIFF, netCDF, and GRIB formats were accessed using the Python packages rasterio (version 1.4.3), xarray (version 2024.9.0), and pygrib (version 2.1.6), respectively, and converted to vector data. Plot-level data were compiled by merging tabular and vector data using the functions merge (package pandas) and sjoin (package geopandas). When the rounded coordinates of NFI plots were beyond any polygons of the vector data, the function sjoin_nearest (package geopandas) was used to join by the nearest distance. Grid-level data for statistical modeling (15-km resolution) was interpolated from tabular and vector data at the coarsest spatial resolution of the obtained datasets (N deposition) by piecewise linear interpolation at grid cell centers using the function griddata of the Python package SciPy (version 1.16.1). Ecoregions were assigned to grid cells by maximum area overlap. Grid cells that did not contain plots were excluded from statistical analyses. The remaining grid cells contained 2.6 ± 1.8 natural and 1.9 ± 1.2 planted plots (mean ± standard deviation). Figures were created using the Python package matplotlib (version 3.10.3). Maps shown in any figures were plotted from vector data using geopandas.

### Statistical analysis

Statistical analysis was performed using R scripts executed with the Python package rpy2 (version 3.5.11; R version 4.5.1). Predictor effects were compared using GLMs with standardized predictors^88^ using the R package glmmTMB (version 1.1.11). Variance inflation factors for confirming low predictor collinearity (function check_collinearity) were computed using the R package performance (version 0.14.0). Goodness-of-fit of GLMs was confirmed by inspecting model residuals (R package DHARMa; version 0.4.7). For the response variable AM tree dominance, which had values in a closed interval between 0 (only EM trees present) and 1 (only AM trees present), a beta distribution was assumed and ordered beta regression was applied (family ordbeta; link function logit). For other response variables, a Gaussian distribution was assumed (family gaussian; link function log). Marginal effect predictions were computed from GLMs using the function predict_response with empirical margins (R package ggeffects; version 2.2.1). Groupwise and overall average marginal effects of predictors were computed from GLMs using the function avg_comparisons (R package marginaleffects; version 0.26.0). Average marginal effects were derived from counterfactual comparisons between two predictor groups or unit-levels^89^.

GLMs are listed in Supplementary Data 1. GLMs were constructed to assess the relationships of AM tree dominance to MAT (GLMs 1 and 2) and the soil C stock, N stock, and their ratio (GLMs 3–8); the change in AM dominance between campaigns T1 and T4 (GLMs 9 and 10); and the drivers of the AM tree dominance change (GLMs 11 and 12). Additional effects accounted for were MAP, basal area^15^, and temperature and precipitation seasonality^9,65^ in GLMs 1 and 2; MAT and MAP^15,40,41^, basal area^15^, species richness^65^, slope inclination^40,41^, and tephra deposition^90^ in GLMs 3–8; and initial basal area and AM tree dominance^15^, MAP change^15^, and exposure to wind and snow disturbance–common abiotic disturbance types in Japan in GLMs 11 and 12. Significance of predictor effects and average marginal effects was tested using Wald Z-tests (two-sided), which assume an asymptotic probability distribution.

To assess the effect of the plot infection status on the AM tree dominance change, GLMs 9 and 10 were modified by adding the plot infection status (infected versus non-infected) and its interaction with the inventory campaign as predictors (GLMs 13 and 14). When grid-level data were analyzed (GLMs 11 and 12), the plot numbers per grid cell were used as weights to account for uneven plot distributions among grid cells (argument weights of the function glmmTMB). Random intercepts by ecoregion (GLMs 1–8, 11, and 12) or by plot nested within ecoregion (GLMs 9, 10, 13, and 14) were included to account for spatial data interdependencies^15^. Variation in the effect of the main predictor by ecoregion was assessed by comparing models with and without random slopes using χ^2^-tests (one-sided; function anova; GLMs 1–10, 13, and 14). Random slopes were deemed non-significant and removed when their inclusion did not significantly reduce the residual sum of squares of the model or when the more complex model failed to converge. Beta distribution means and 95% confidence intervals of AM tree dominance were estimated from GLMs of the form AM tree dominance ~1 using the function emmeans (R package emmeans; version 1.11.1).

Low spatial autocorrelation of GLM residuals was confirmed in spline correlograms^91^ (function spline.correlog in the R package ncf, version 1.3.3). Spatial autocorrelation at short distances between plots or grid cells was either low (≤ 0.10; GLMs 1–4 and 8) or insignificant (GLMs 5–7 and 9–15; Supplementary Figs. 38–41). Furthermore, the sensitivity of GLMs 1–4 and 9–12 to potentially incorrect mycorrhizal type assignments was tested by recomputing predictor effects after reassigning the mycorrhizal types of tree species with contradictory reports, in line with a previous study^92^. Reassignments were made for randomly selected subsets of tree species in 100 iterations (Supplementary Data 2).

Distribution changes in AM tree dominance between T1 and T4 were visualized with kernel density estimates computed with Gaussian kernels and default bandwidth estimation according to Scott’s rule (function gaussian_kde; package SciPy). To map the distribution of common types of forest disturbance across Japan, a point layer of plots where a disturbance was recorded in any of the four NFI campaigns was converted to a continuous density layer with kernel density estimation (function gaussian_kde; package SciPy). Thereby, the bandwidth was estimated as half of the diagonal length of a 15-km grid square^93^. Positive correlations between wind exposure and wind disturbance density and between maximum snow mass and snow disturbance density (log-transformed variables) were assessed using Spearman’s correlation coefficients (one-sided; function spearmanr; package SciPy).

### Reporting summary

Further information on research design is available in the Nature Portfolio Reporting Summary linked to this article.

## Supplementary information

Supplementary Information

Description of Additional Supplementary Information

Supplementary Data 1

Supplementary Data 2

Reporting Summary

Transparent Peer Review file

## Source data

Source Data

## Data Availability

The plot- and grid-level data generated in this study have been deposited in Figshare^94^ under accession code https://doi.org/10.6084/m9.figshare.29985265. Data files are contained in a compressed file archive (Data_for_analysis.zip). The NFI data^58^, elevation data^71^, averaged climate data^74^, and snow water equivalent data^87^ for Japan used in this study are available from the relevant government agencies via the links cited in this sentence. The tephra deposition data^72^ used in this study are available via Zenodo^73^. The ecoregion data^66,67,69^ used in this study are available via the Google Earth Engine^68^. The plant name data used in this study are available via online indices^61,62^. The mycorrhizal type data used in this study are available in the FungalRoot database^47,63^ via PlutoF^64^ and the supplementary materials of Steidinger et al.^9^ and Soudzilovskaia et al.^63^. The elevation data outside of Japan used in this study are available from a global digital elevation/terrain model^85^. The climate time-series data used in this study are available in the TerraClimate dataset^75^. The wind speed data used in this study are available from ERA5-Land^84^. The global tree symbiosis map data used in this study are available in the supplementary materials of Steidinger et al.^9^. Details on associated licenses are provided in the Figshare dataset (README_data.txt). Online translation tools may be used to navigate resources in Japanese. The vector data used to draw East Asian country borders^95^ in Fig. 1a were obtained from https://www.geoboundaries.org/ under the CC BY 4.0 license (https://creativecommons.org/licenses/by/4.0/). The vector data used to draw ecoregions and Japanese coastlines in all remaining figures were obtained via the Google Earth Engine^68^ under the CC BY 4.0 license (https://creativecommons.org/licenses/by/4.0). Restrictions apply to the availability of N deposition^76^ and NFSCI soil data which were used under license for the current study and are not publicly available. N deposition data are available upon request to the corresponding author of Chatani et al.^76^. NFSCI soil data are available upon request to the corresponding author of this study and with permission of the Japanese Forestry Agency which owns the data. The agency is currently preparing operations towards publication of the NFSCI data within an unspecified timeframe. Publication of the extensive government-owned data collected on private and public land requires careful consideration of data privacy and safety to prevent exploitation or misuse. Until formal publication by the Japanese Forestry Agency, the NFSCI data can only be made available to qualified individual researchers for the validation and verification of our study results, contingent on approval by the relevant department of the Japanese Forestry Agency. Responses to data access requests may take up to four weeks. Information on predictor effects in GLMs 1–15 is summarized in Supplementary Data 1. Mycorrhizal type assignments are provided in Supplementary Data 2. Source data are provided with this paper.

## Code availability

The custom computer code used to generate the statistical results has been deposited in Figshare under accession code https://doi.org/10.6084/m9.figshare.29985265. The code consists of the main analysis script (data_analysis_script.py) and two collections of functions (python_functions.py, R_functions.R). The code is mainly written in Python, with R functions used for statistical testing. Instructions on how the code is executed are provided (README_code.txt). All files are contained in a compressed file archive (Code_for_analysis.zip).
