# Supplementary material for: Broad-scale shift in dominance of arbuscular mycorrhizal trees along the Japanese archipelago associated with tree diseases and climate warming
Source: Nat Commun. 2026 Sep 11;17:10731. doi: 10.1038/s41467-026-77711-w (PMC13658775; doi:10.1038/s41467-026-77711-w)
Supplement: Supplementary file 1 [file 41467_2026_77711_MOESM1_ESM.pdf]

Supplementary Information

Broad-scale shift in dominance of arbuscular mycorrhizal trees along the Japanese archipelago associated with tree diseases and climate warming

Holger Schaefer[1*], Naoyuki Yamashita[2], Shoji Hashimoto[1,3], Yoshiyuki Inagaki[2], Ayumi Kawanishi[1], Satoru Chatani[4], Hikari Shimadera[5], Hitomi Furusawa[1], Akihiro Imaya[1]

[1] Department of Forest Soils, Forestry and Forest Products Research Institute (FFPRI), Tsukuba, Japan

[2] Shikoku Research Center, Forestry and Forest Products Research Institute (FFPRI), Kochi, Japan

[3] Graduate School of Agricultural and Life Sciences, The University of Tokyo, Tokyo, Japan

[4] National Institute for Environmental Studies (NIES), Tsukuba, Japan

[5] Graduate School of Engineering, The University of Osaka, Suita, Japan

* Corresponding author

E-mail: schaefer.holger.24x@kyoto-u.jp

Supplementary Table 1 | Comparison of generalized linear models (GLMs) with and without random slopes using χ^2-tests (one-sided). Sample sizes for the GLMs are provided in Supplementary Data 1. AM, MAT, and df stand for arbuscular mycorrhizal, mean annual temperature, and degrees of freedom, respectively.

GLM	Dependent variable	Random effect	df	AIC	χ^2 value	df(χ^2)	p value
1	AM tree dominance in natural forests	(1 | ecoregion)	10	2919		-	-
		(MAT | ecoregion)	12	2906	17.22	2	1.82×10^{-4}
2	AM tree dominance in planted forests	(1 | ecoregion)	10	3114		-	-
		(MAT | ecoregion)	12	2933	185.12	2	6.34×10^{-41}
3	Soil CN stock ratio in natural forests	(1 | ecoregion)	10	5764		-	-
		(soil CN | ecoregion)	12	5768	0.33	2	0.849
4	Soil CN stock ratio in planted forests	(1 | ecoregion)	10	5329		-	-
		(soil CN | ecoregion)	12	NA	NA	2	NA*
5	Soil C stock ratio in natural forests	(1 | ecoregion)	10	5379		-	-
		(soil C | ecoregion)	12	5381	1.78	2	0.411
6	Soil C stock ratio in planted forests	(1 | ecoregion)	10	5126			-
		(soil C | ecoregion)	12	5130	0.06	2	0.969
7	Soil N stock ratio in natural forests	(1 | ecoregion)	10	-628		-	-
		(soil N | ecoregion)	12	-624	0.03	2	0.987
8	Soil N stock ratio in planted forests	(1 | ecoregion)	10	-622			-
		(soil N | ecoregion)	12	-619	1.27	2	0.529
9	AM tree dominance in natural forests	(1 | ecoregion/plot)	7	-1340		-	-
		(1 | ecoregion/plot) + (0+campaign | ecoregion)	10	-1384	49.98	3	8.07×10^{-11}
10	AM tree dominance in planted forests	(1 | ecoregion/plot)	7	-82		-	
		(1 | ecoregion/plot) + (0+campaign | ecoregion)	10	-118	42.00	3	4.01×10^{-9}
13	AM tree dominance in natural forests	(1 | ecoregion/plot)	9	-1594			-
		(1 | ecoregion/plot) + (0+campaign | ecoregion)	12	NA	NA	3	NA
14	AM tree dominance in planted forests	(1 | ecoregion/plot)	9	-353		-	-
		(1 | ecoregion/plot) + (0+campaign | ecoregion)	12	NA	NA	3	NA

*Estimates are missing when the model did not converge properly. Overparameterization was assumed in these cases.

Supplementary Table 2 | Ecoregion-specific average marginal effects of mean annual temperature (MAT) on arbuscular mycorrhizal (AM) tree dominance computed from generalized linear models (GLMs) 1 and 2. Effect significance was determined using Wald Z-tests (two-sided). Sample sizes indicate the numbers of plots. CI stands for confidence interval. Residual degrees of freedom are 5866 (GLM 1) and 5619 (GLM 2).

Dependent variable	Pre-dictor	Ecoregion	n	Estimate	Standard error	95% CI	z value	p value
AM tree dominance in natural forest plots (GLM 1)	MAT	Central alpine	286	0.036	0.005	0.026- 0.045	7.30	2.83×10^{-13}
		Japan-Sea lowland	373	0.017	0.005	0.007- 0.027	3.27	0.001
		Japan-Sea montane	1545	0.028	0.005	0.018 - 0.039	5.38	7.33×10^{-8}
		Northern lowland	399	0.031	0.005	0.021 - 0.041	6.03	1.59×10^{-9}
		Northern montane	1056	0.044	0.004	0.036 - 0.052	10.29	8.05×10^{-25}
		Pacific lowland	1685	0.019	0.005	0.008 - 0.029	3.59	3.26×10^{-4}
		Pacific montane	534	0.024	0.005	0.014 - 0.035	4.54	5.71×10^{-6}
AM tree dominance in planted forest plots (GLM 2)	MAT	Central alpine	120	0.056	0.012	0.034 - 0.079	4.86	1.17×10^{-6}
		Japan-Sea lowland	303	0.011	0.008	-0.005 - 0.026	1.38	0.169
		Japan-Sea montane	1298	0.060	0.010	0.041 - 0.080	6.07	1.31×10^{-9}
		Northern lowland	254	0.026	0.009	0.009 - 0.043	3.02	0.003
		Northern montane	437	0.007	0.008	-0.009 - 0.023	0.85	0.395
		Pacific lowland	2309	0.003	0.007	-0.012 - 0.018	0.38	0.700
		Pacific montane	910	0.054	0.009	0.037 - 0.071	6.30	3.03×10^{-10}

Supplementary Table 3 | Ecoregion-specific average marginal effects of inventory campaign (T1 versus T4) on arbuscular mycorrhizal (AM) tree dominance computed from generalized linear models (GLMs) 9 and 10. Effect significance was determined using Wald Z-tests (two-sided). Sample sizes indicate the numbers of plots. CI stands for confidence interval. Residual degrees of freedom are 6860 (GLM 9) and 4008 (GLM 10).

Dependent variable	Predictor	Ecoregion	n	Estimate	Standard error	95% CI	z value	p value
AM tree dominance in natural forest plots (GLM 9)	Inventory campaign	Central alpine	180	0.007	0.009	-0.010 - 0.023	0.79	0.428
		Japan-Sea lowland	263	0.077	0.011	0.056 - 0.098	7.12	1.12×10^{-12}
		Japan-Sea montane	960	0.031	0.009	0.012 - 0.049	3.27	0.001
		Northern lowland	239	0.005	0.009	-0.013 - 0.023	0.57	0.568
		Northern montane	556	0.003	0.008	-0.013 - 0.019	0.40	0.688
		Pacific lowland	930	0.029	0.010	0.009 - 0.049	2.83	0.005
		Pacific montane	307	0.019	0.009	0.001 - 0.037	2.07	0.038
AM tree dominance in planted forest plots (GLM 10)	Inventory campaign	Central alpine	40	0.035	0.012	0.012 - 0.059	2.93	0.003
		Japan-Sea lowland	144	0.032	0.009	0.015 - 0.050	3.65	2.63×10^{-4}
		Japan-Sea montane	458	0.027	0.007	0.014 - 0.040	4.09	4.39×10^{-5}
		Northern lowland	87	0.021	0.013	-0.003 - 0.046	1.70	0.090
		Northern montane	167	-0.006	0.008	-0.022 - 0.009	-0.79	0.431
		Pacific lowland	802	-0.000	0.004	-0.009 - 0.008	-0.11	0.911
		Pacific montane	311	0.023	0.005	0.013 - 0.033	4.52	6.05×10^{-6}

Supplementary Table 4 | Dataset formats and resolutions.

Data	Type	Format	Resolution
National Forest Inventory (NFI)	Tabular	CSV	-
Species names	Tabular	CSV	-
Mycorrhizal types (species-level)	Tabular	CSV	-
Mycorrhizal types (genus-level)	Tabular	CSV	-
National Forest Soil Carbon Inventory (NFSCI)	Tabular	CSV	-
Tephra deposition	Grid	GeoTIFF	1 km
Elevation (for slope inclination estimation)	Grid	GeoTIFF	0.1 km (converted from 0.01 km)
Mean annual temperature (MAT), mean annual precipitation (MAP)	Grid	Shapefile	1 km
MAT, MAP change	Grid	netCDF	4 km
N deposition	Grid	GeoTIFF	15 km
Wind speed, direction	Grid	GRIB	0.1◦
Elevation (for wind exposure estimation)	Grid	GeoTIFF	1/60°
Snow water equivalents	Grid	CSV	1 km
Ecoregions	Irregular polygons	Shapefile	-

Supplementary Table 5 | Abbreviated ecoregion names and their equivalents in Olson et al. (2001).

Ecoregion after Olson (2001)	Abbreviated name
Honshu alpine conifer forests	Central alpine
Hokkaido montane conifer forests	Northern montane
Nihonkai montane deciduous forests	Japan-Sea montane
Taiheiyo montane deciduous forests	Pacific montane
Hokkaido deciduous forests	Northern lowland
Nihonkai evergreen forests	Japan-Sea lowland
Taiheiyo evergreen forests	Pacific lowland

Supplementary Table 6 | Plot attributes (mean ± standard deviation) by ecoregion. Climate data is based on averaged climate variables for 1991–2020. Mean annual temperature (MAT), mean annual precipitation (MAP), and elevation are summarized for National Forest Inventory (NFI) plots in inventory campaign T4. Slope inclination is summarized for National Forest Soil Carbon Inventory (NFSCI) plots in inventory campaign T2. Sample sizes (numbers of plots) are provided in brackets.

Ecoregion	Elevation (m)	Slope inclination (◦)	MAT (◦C)	MAP (mm)
Central alpine	924 ± 486 (484)	26 ± 10 (72)	7.1 ± 2.0 (501)	1889 ± 466 (501)
Japan-Sea lowland	281 ± 221 (794)	24 ± 9 (139)	12.7 ± 1.4 (813)	2116 ± 356 (813)
Japan-Sea montane	548 ± 372 (3313)	24 ± 9 (547)	9.0 ± 1.9 (3371)	1945 ± 568 (3371)
Northern lowland	223 ± 164 (735)	19 ± 8 (124)	6.0 ± 1.0 (739)	1242 ± 270 (739)
Northern montane	444 ± 291 (1714)	19 ± 10 (292)	4.3 ± 1.4 (1716)	1259 ± 292 (1716)
Pacific lowland	343 ± 251 (4491)	25 ± 8 (798)	13.7 ± 1.8 (4543)	2152 ± 634 (4543)
Pacific montane	721 ± 445 (1751)	28 ± 9 (293)	10.2 ± 2.2 (1812)	2004 ± 699 (1812)

Supplementary Table 7 | Plot vegetation attributes (mean ± standard deviation) by ecoregion. Data are summarized for National Forest Inventory (NFI) plots in inventory campaign T4. Stand ages were estimated in T1. Sample sizes (numbers of plots) are provided in brackets.

Ecoregion	Regeneration type	Most common tree genera	Basal area (m^2 ha^{-1})	Stand age (years)	Species richness
Central alpine	natural	*Fagus, Quercus, Betula*	28 ± 21 (366)	107 ± 63 (352)	6.8 ± 5.4 (366)
	planted	*Cryptomeria, Larix, Pinus*	36 ± 24 (135)	38 ± 21 (131)	6.3 ± 4.7 (135)
Japan-Sea lowland	natural	*Abies, Betula, Quercus*	28 ± 19 (454)	49 ± 24 (436)	10.4 ± 6.7 (454)
	planted	*Abies, Larix, Picea*	43 ± 28 (359)	37 ± 17 (358)	5.4 ± 5.1 (359)
Japan-Sea montane	natural	*Fagus, Quercus, Acer*	27 ± 20 (1932)	73 ± 50 (1893)	7.6 ± 5.4 (1932)
	planted	*Cryptomeria, Chamaecyparis, Larix*	45 ± 26 (1439)	37 ± 16 (1426)	5.3 ± 4.7 (1439)
Northern lowland	natural	*Quercus, Pinus, Carpinus*	24 ± 16 (466)	78 ± 36 (456)	6.6 ± 4.2 (466)
	planted	*Cryptomeria, Chamaecyparis, Pinus*	28 ± 16 (273)	35 ± 16 (273)	6.1 ± 4.0 (273)
Northern montane	natural	*Quercus, Acer, Abies*	24 ± 17 (1250)	90 ± 34 (1236)	5.9 ± 4.1 (1250)
	planted	*Abies, Larix, Betula*	28 ± 15 (466)	36 ± 19 (465)	5.7 ± 3.7 (466)
Pacific lowland	natural	*Quercus, Pinus, Cryptomeria*	30 ± 21 (2001)	42 ± 27 (1960)	10.8 ± 6.6 (2001)
	planted	*Cryptomeria, Chamaecyparis, Quercus*	48 ± 26 (2542)	36 ± 15 (2522)	5.2 ± 4.9 (2542)
Pacific montane	natural	*Quercus, Castanopsis, Cryptomeria*	26 ± 21 (727)	61 ± 50 (719)	8.7 ± 6.8 (727)
	planted	*Cryptomeria, Chamaecyparis, Quercus*	45 ± 28 (1085)	38 ± 15 (1075)	4.6 ± 5.0 (1085)

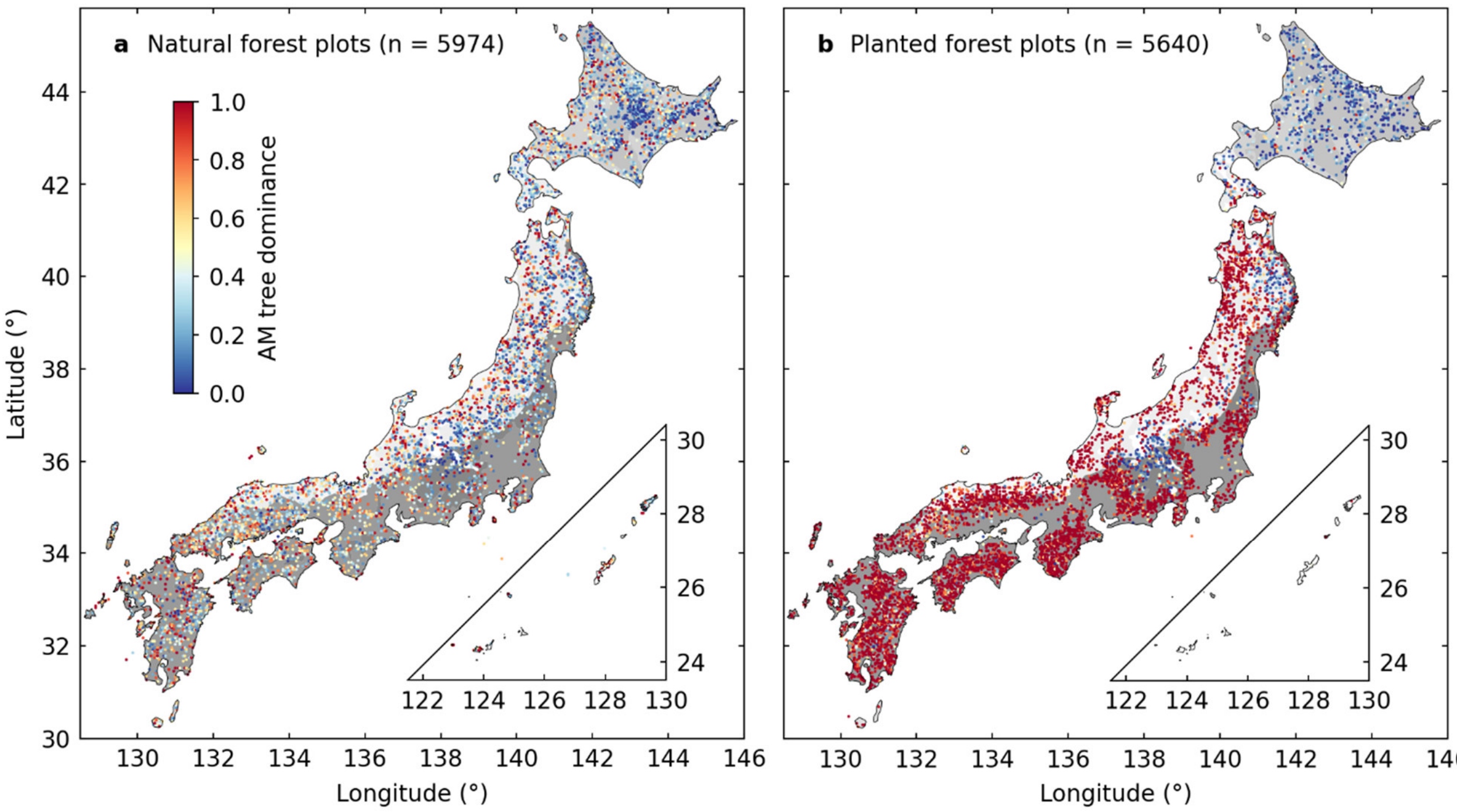

Supplementary Figure 1 | Distribution of arbuscular mycorrhizal (AM) tree dominance in National Forest Inventory (NFI) campaign T4 (2014 –2019). a AM tree dominance in natural forest plots. **b** AM tree dominance in planted forest plots. Ecoregions of Japan after Olson et al. (2001)[66,67,69] are indicated in shades of grey. Ecoregions and coastlines were drawn based on data from RESOLVE[68]. See Figure 1 and Supplementary Tables 5–7 for more details on the ecoregions.

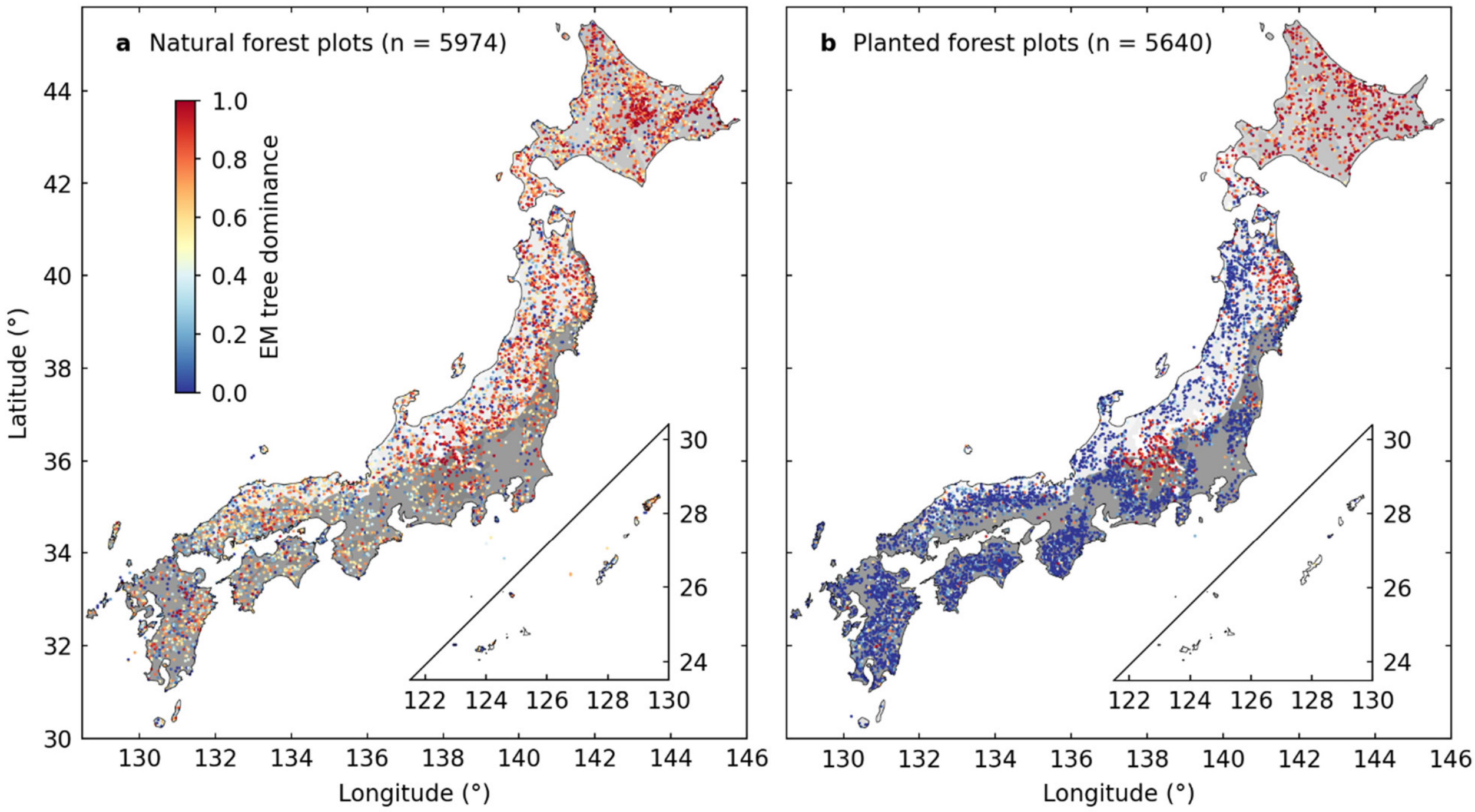

Supplementary Figure 2 | Distribution of ectomycorrhizal (EM) tree dominance in National Forest Inventory (NFI) campaign T4 (2014–2019). a EM tree dominance in natural forest plots. **b** EM tree dominance in planted forest plots. Ecoregions of Japan after Olson et al. (2001)[66,67,69] are indicated in shades of grey. Ecoregions and coastlines were drawn based on data from RESOLVE[68]. See Figure 1 and Supplementary Tables 5–7 for more details on the ecoregions.

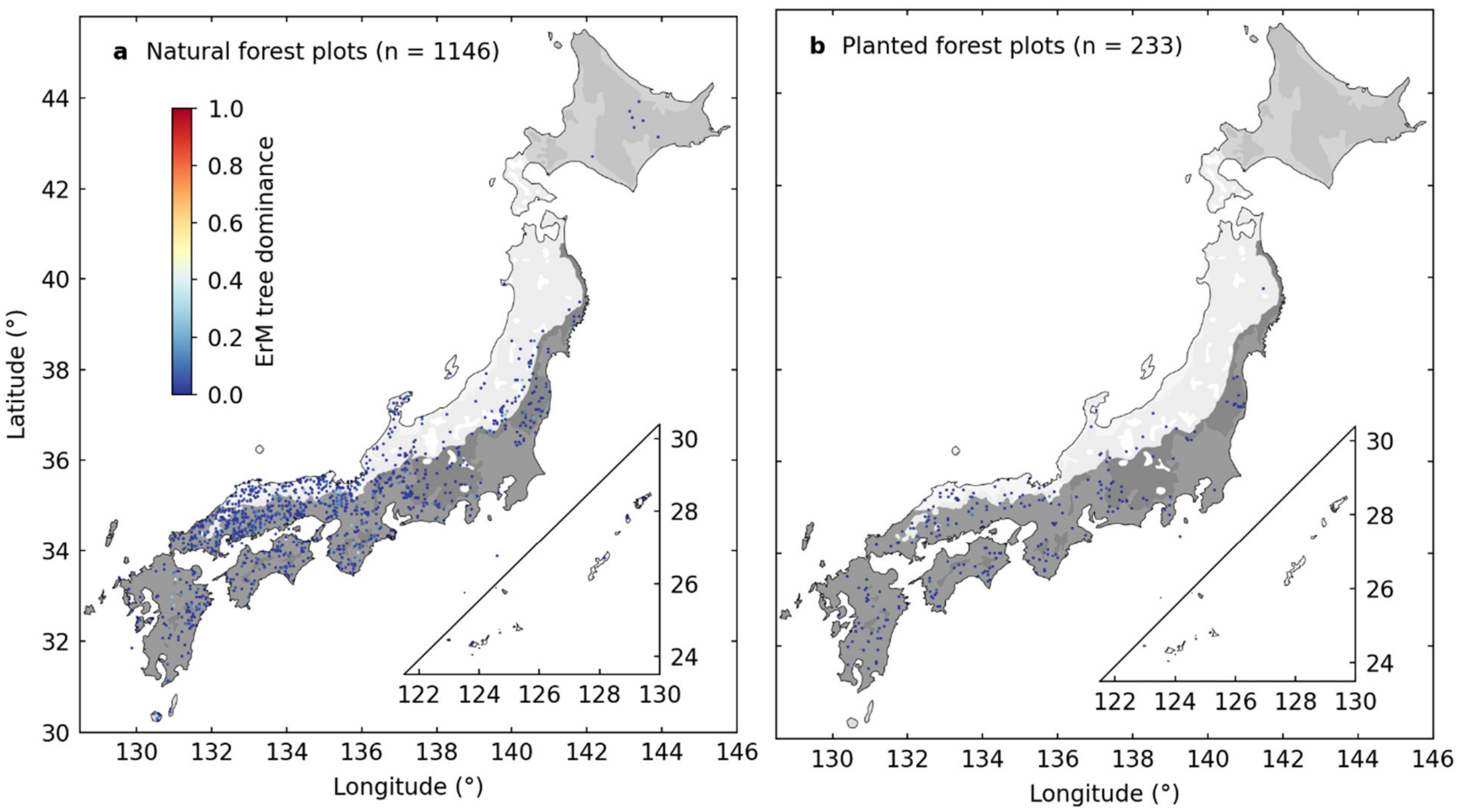

Supplementary Figure 3 | Distribution of ericoid mycorrhizal (ErM) tree dominance in National Forest Inventory (NFI) campaign T4 (2014 –2019). a ErM tree dominance in natural forest plots (values > 0). **b** ErM tree dominance in planted forest plots (values > 0). Ecoregions of Japan after Olson et al. (2001)[66,67,69] are indicated in shades of grey. Ecoregions and coastlines were drawn based on data from RESOLVE[68]. See Figure 1 and Supplementary Tables 5–7 for more details on the ecoregions.

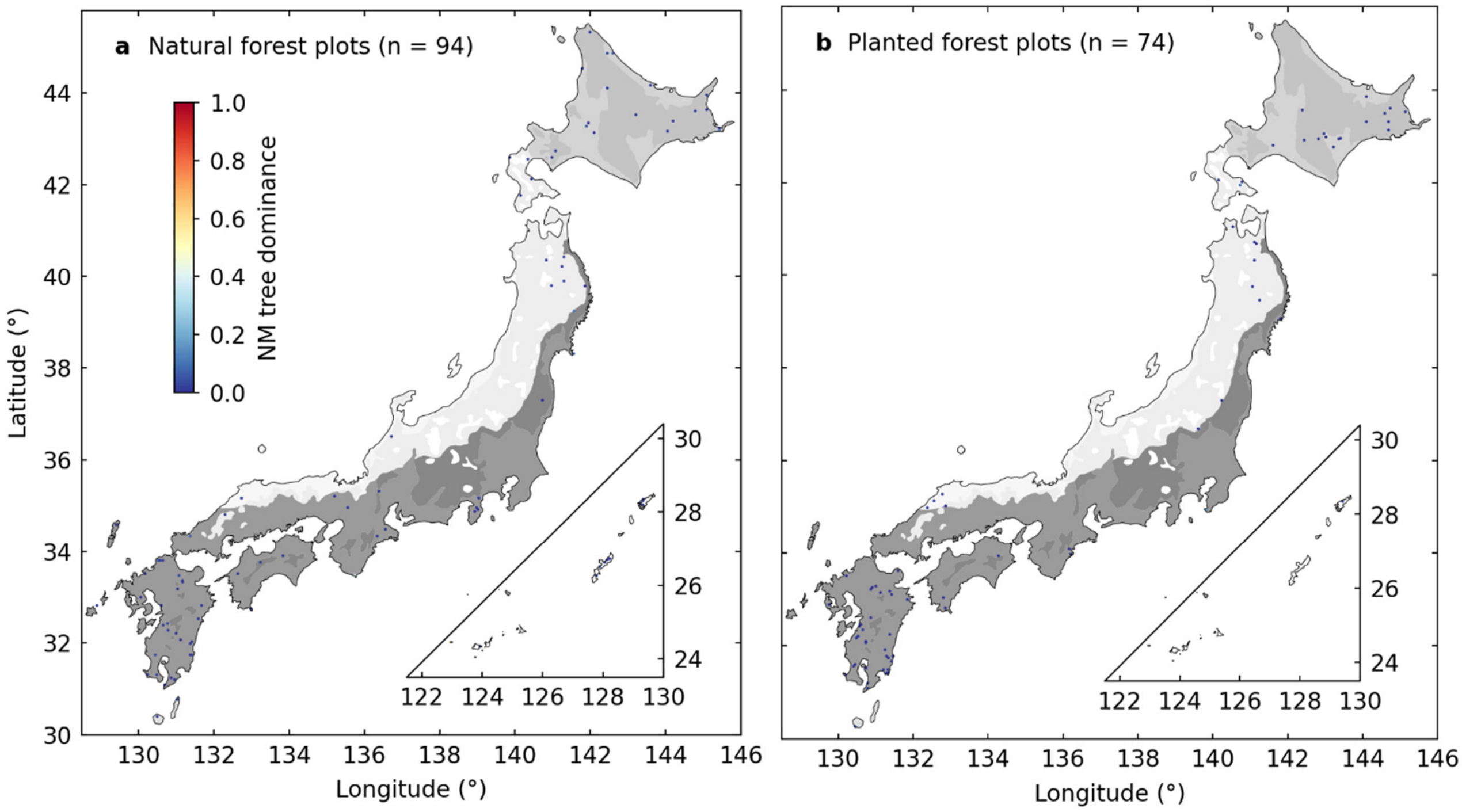

Supplementary Figure 4 | Distribution of non-mycorrhizal (NM) tree dominance in National Forest Inventory (NFI) campaign T4 (2014 –2019). a NM tree dominance in natural forest plots (values > 0). **b** NM tree dominance in planted forest plots (values > 0). NM tree dominance values resulted from the basal area contributions of AM-NM tree species. Obligate NM tree species were not identified in the NFI dataset. Ecoregions of Japan after Olson et al. (2001)[66,67,69] are indicated in shades of grey. Ecoregions and coastlines were drawn based on data from RESOLVE[68]. See Figure 1 and Supplementary Tables 5–7 for more details on the ecoregions.

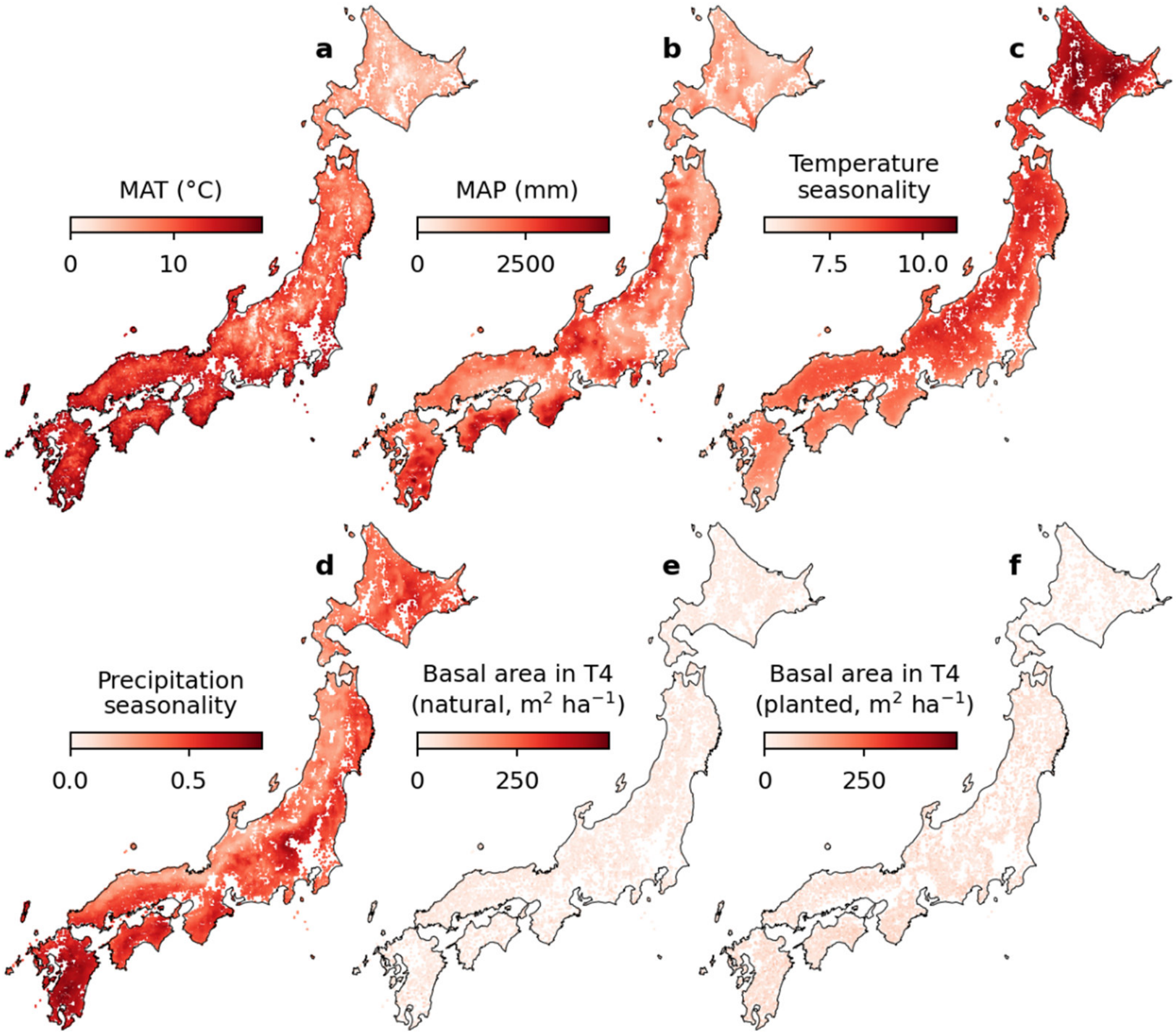

Supplementary Figure 5 | Distributions of predictors in generalized linear models (GLMs) 1–2 among National Forest Inventory (NFI) plots. Distributions among natural (n = 5878) and planted plots (n = 5631) in inventory campaign T4 are shown for (**a**) mean annual temperature (MAT), (**b**) mean annual precipitation (MAP), (**c**) temperature seasonality, (**d**) precipitation seasonality, and (**e, f**) basal area. Coastlines were drawn based on the total extent of all temperate ecoregions of Japan after Olson et al. (2001)[66,67,69] using data from RESOLVE[68].

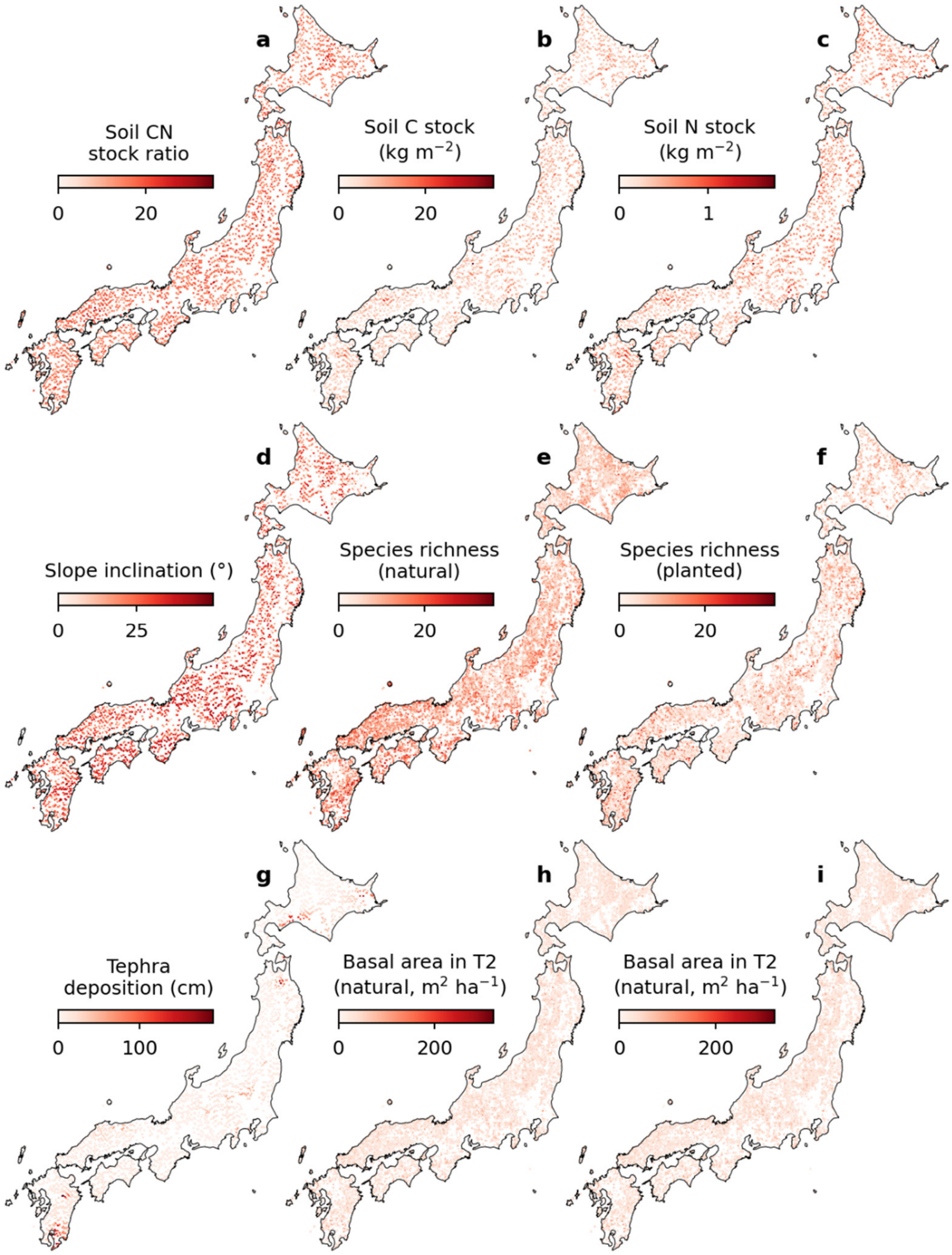

Supplementary Figure 6 | Distributions of predictors and dependent variables in generalized linear models (GLMs) 3–8 among National Forest Inventory (NFI) plots. a Distributions among natural (n = 1094) and planted plots (n = 1068) in inventory campaign T2 are shown for (**a**) the soil CN stock ratio, (**b**) soil C stock, (**c**) soil N stock, (**d**) slope inclination, (**e, f**) species richness, (**g**) tephra deposition, and (**h, i**) basal area. MAT and MAP distributions are shown in Supplementary Fig. 5. Coastlines were drawn based on the total extent of all temperate ecoregions of Japan after Olson et al. (2001)[66,67,69] using data from RESOLVE[68].

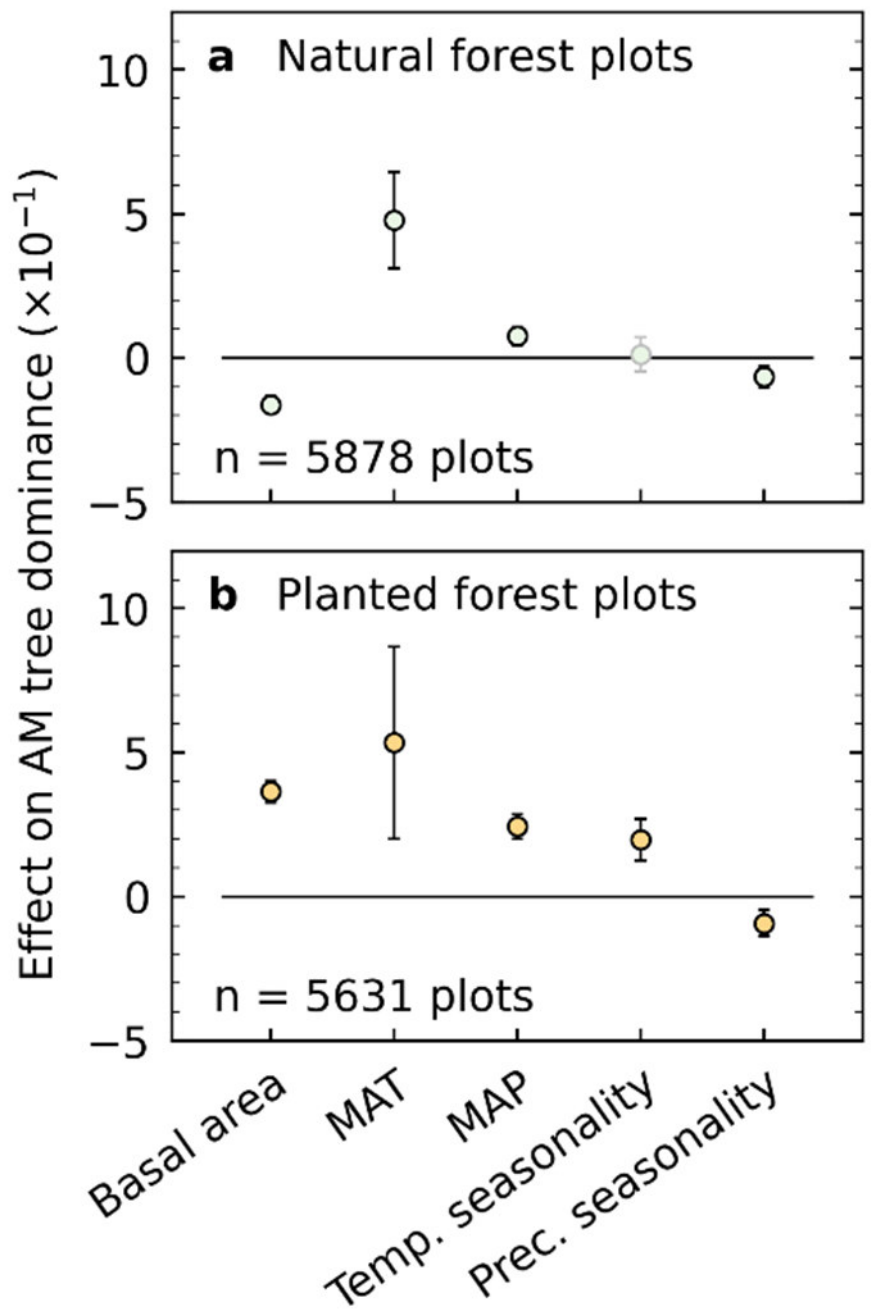

Supplementary Figure 7 | Predictor effect size comparison for generalized linear models (GLMs) 1 and 2. a Predictor effect sizes in natural forest plots. **b** Predictor effect sizes in planted forest plots. Error bars indicate 95% confidence intervals. Dot colors correspond to the mean AM tree dominance of the analyzed plots (color legend in Fig. 1c). Effect sizes were computed from GLMs with standardized predictors (Supplementary Data 1). Dots with black borders indicate significant effects ($p < 0.05$; Wald Z-test, two-sided). P values from left to right (**a**): 1.37×10^{-27}, 2.04×10^{-8}, 7.69×10^{-6}, 0.701, and 4.26×10^{-4}. P values from left to right (**b**): 2.43×10^{-81}, 0.002, 2.53×10^{-30}, 7.98×10^{-8}, and 4.14×10^{-5}. Marginal effect predictions with underlying data distributions are shown in Fig. 2a, b and Supplementary Figs. 9–10. Source data are provided as a Source Data file.

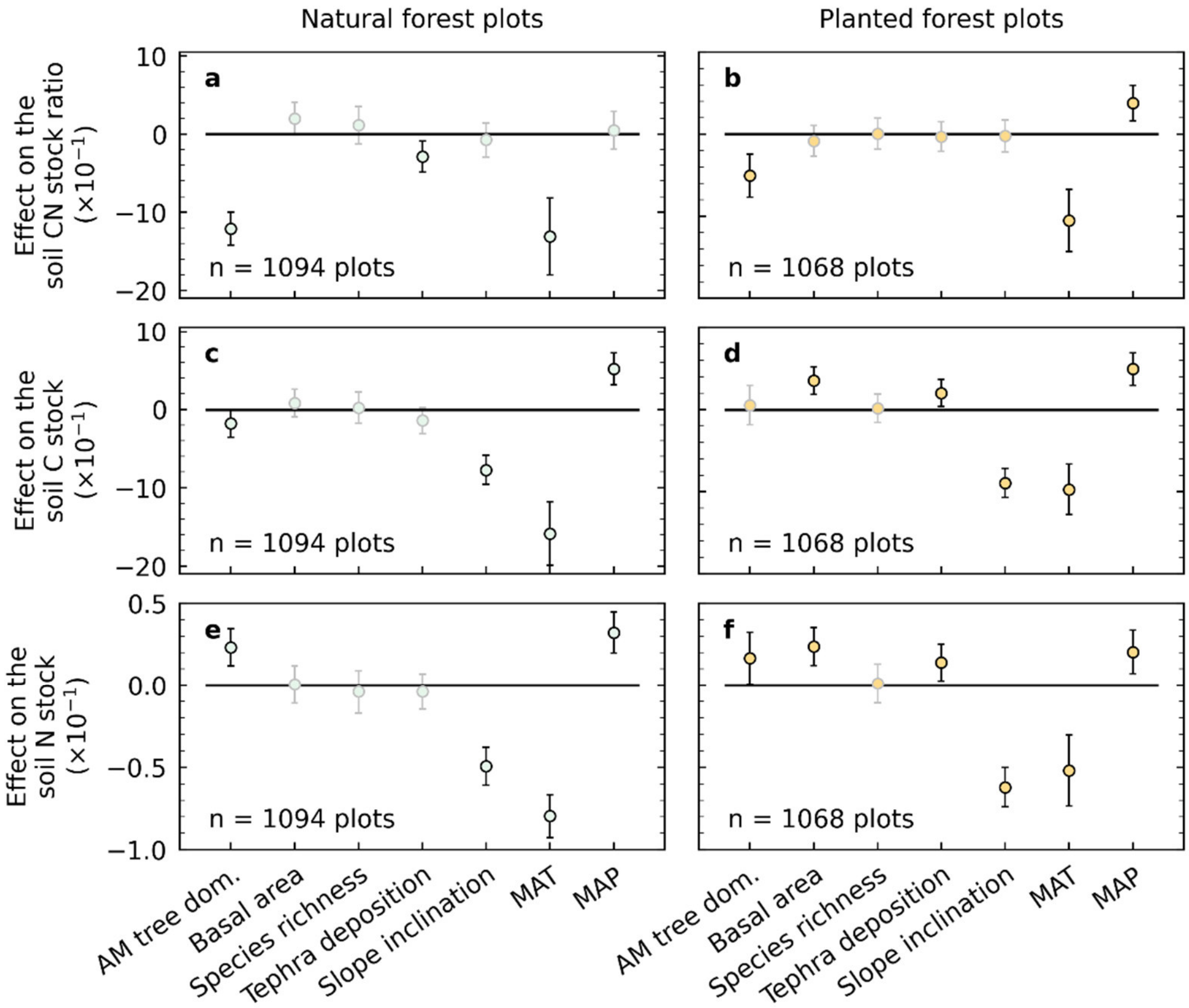

Supplementary Figure 8 | Predictor effect size comparison for generalized linear models (GLMs) 3–8. a-b Predictor effect sizes on the soil CN stock ratio in natural and planted forest plots. **c-d** Predictor effect sizes on the soil C stock in natural and planted forest plots. **e-f** Predictor effect sizes on the soil N stock in natural and planted forest plots. Errors bars indicate 95% confidence intervals. Dot colors correspond to the mean AM tree dominance of the analyzed plot populations (color legend in Fig. 1c). Effect sizes were computed from GLMs with standardized predictors (Supplementary Data 1). Dots with black borders indicate significant effects ($p < 0.05$; Wald Z-test, two-sided). Marginal effect plots with underlying data distributions are shown in Supplementary Figs. 11–16. Exact p values are provided in the source data. Source data are provided as a Source Data file.

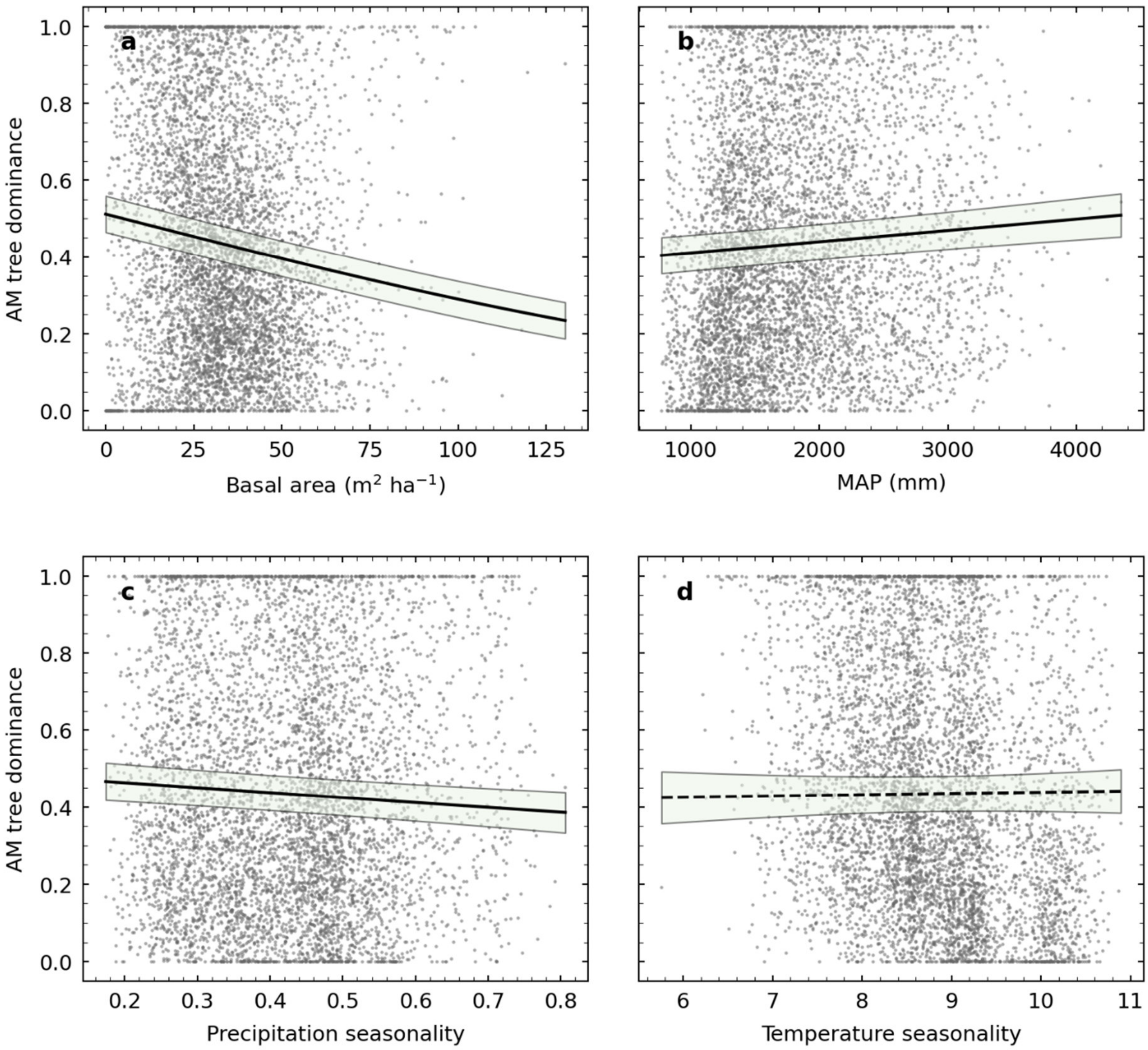

Supplementary Figure 9 | Marginal effect predictions for generalized linear model (GLM) 1 with sample size n = 5878 plots. Marginal effect predictions are shown for (**a**) basal area, (**b**) mean annual precipitation (MAP), (**c**) precipitation seasonality, and (**d**) temperature seasonality. Marginal effect predictions (lines) are shown with 95% confidence intervals (shades). Marginal effect predictions of insignificant predictors ($p < 0.05$; Wald Z-test, two-sided) have dashed lines. Exact p values for predictor effects are provided in Supplementary Data 1. Grey dots show the underlying data. Shade colors correspond to the mean AM tree dominance of the analyzed plots (color legend in Fig. 1c). Source data are provided as a Source Data file.

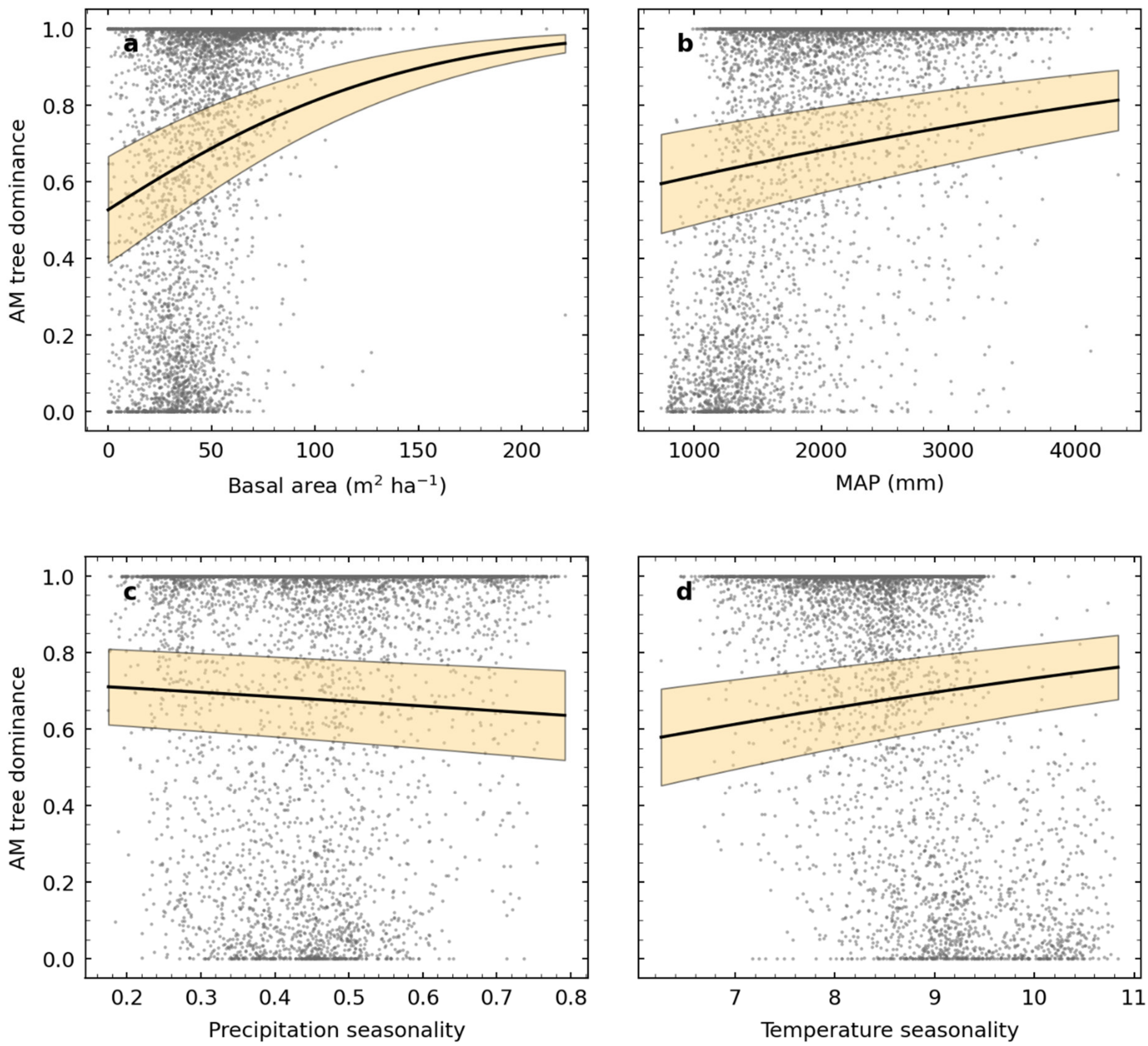

Supplementary Figure 10 | Marginal effect predictions for generalized linear model (GLM) 2 with sample size n = 5631 plots. Marginal effect predictions are shown for (**a**) basal area, (**b**) mean annual precipitation (MAP), (**c**) precipitation seasonality, and (**d**) temperature seasonality. Marginal effect predictions (lines) are shown with 95% confidence intervals (shades). Marginal effect predictions of insignificant predictors ($p < 0.05$; Wald Z-test, two-sided) have dashed lines. Exact p values for predictor effects are provided in Supplementary Data 1. Grey dots show the underlying data. Shade colors correspond to the mean AM tree dominance of the analyzed plots (color legend in Fig. 1c). Source data are provided as a Source Data file.

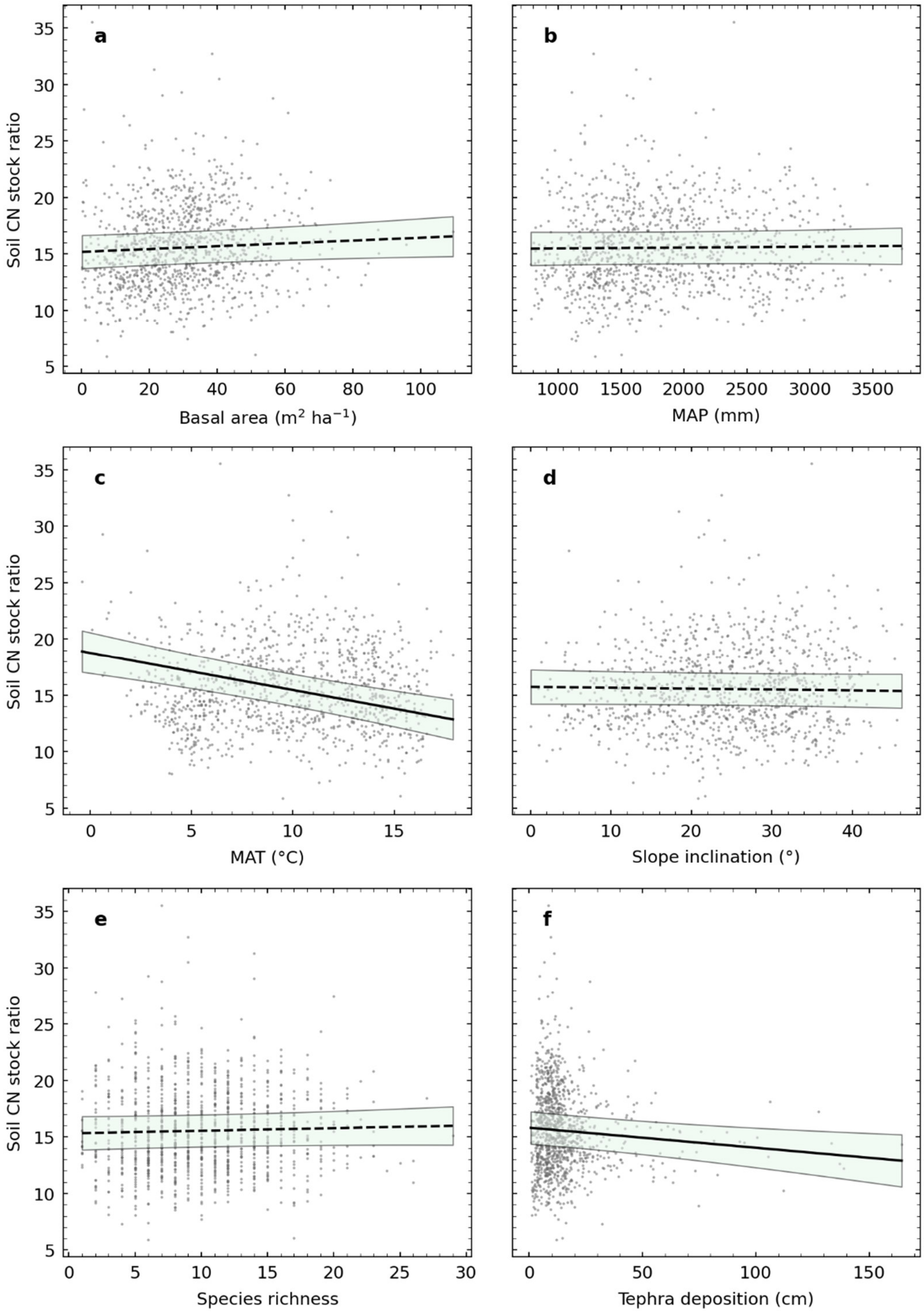

Supplementary Figure 11 | Marginal effect predictions for generalized linear model (GLM) 3 with sample size n = 1094 plots. Marginal effect predictions are shown for (**a**) basal area, (**b**) mean annual precipitation (MAP), (**c**) mean annual temperature (MAT), (**d**) slope inclination, (**e**) species richness, and (**f**) tephra deposition. Marginal effect predictions (lines) are shown with 95% confidence intervals (shades). Marginal effect predictions of insignificant predictors ($p < 0.05$; Wald Z-test, two-sided) have dashed lines. Exact p values for predictor effects are provided in Supplementary Data 1. Grey dots show the underlying data. Shade colors correspond to the mean AM tree dominance of the analyzed plots (color legend in Fig. 1c). Source data are provided as a Source Data file.

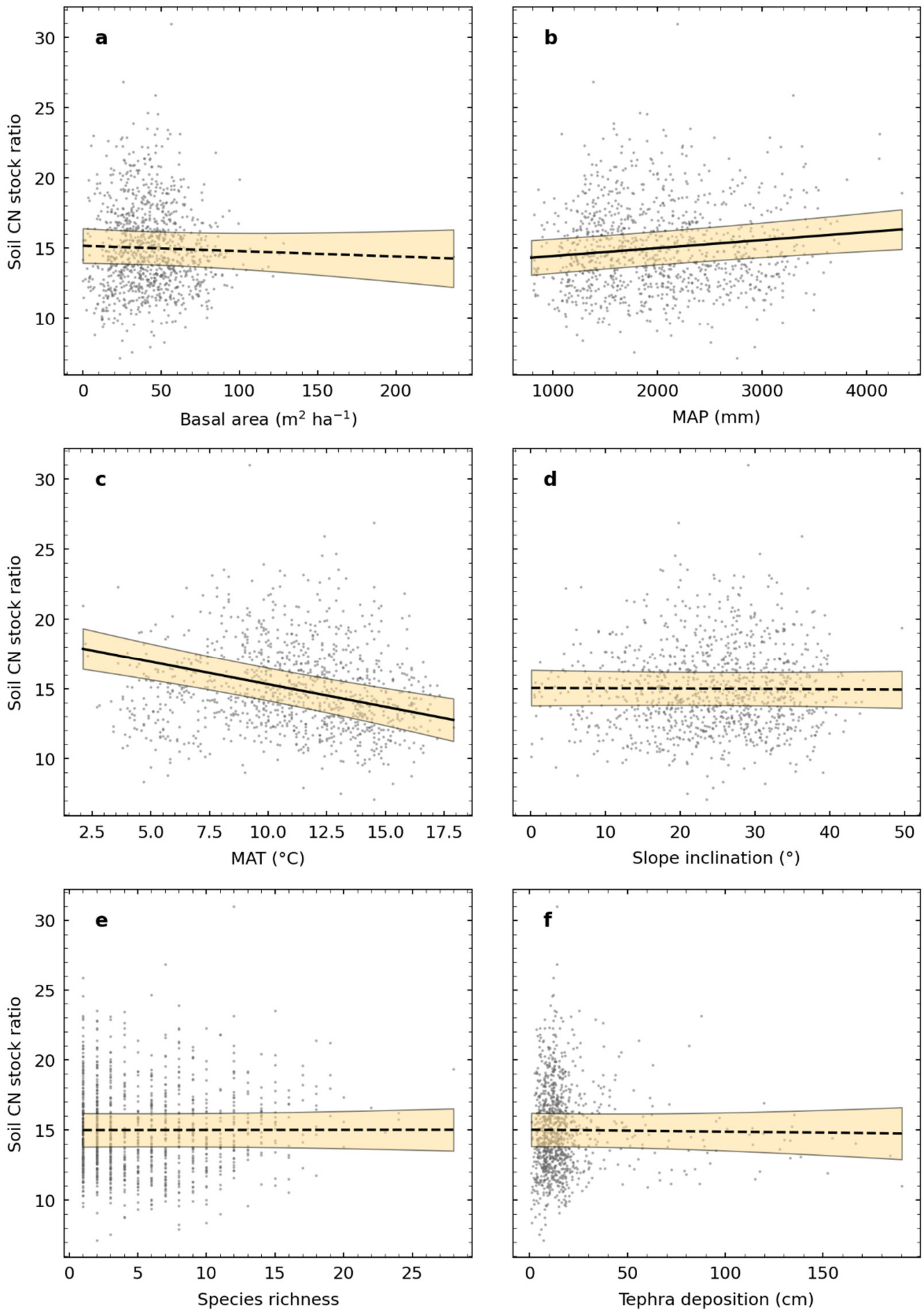

Supplementary Figure 12 | Marginal effect predictions for generalized linear model (GLM) 4 with sample size n = 1068 plots. Marginal effect predictions are shown for (**a**) basal area, (**b**) mean annual precipitation (MAP), (**c**) mean annual temperature (MAT), (**d**) slope inclination, (**e**) species richness, and (**f**) tephra deposition. Marginal effect predictions (lines) are shown with 95% confidence intervals (shades). Marginal effect predictions of insignificant predictors ($p < 0.05$; Wald Z-test, two-sided) have dashed lines. Exact p values for predictor effects are provided in Supplementary Data 1. Grey dots show the underlying data. Shade colors correspond to the mean AM tree dominance of the analyzed plots (color legend in Fig. 1c). Source data are provided as a Source Data file.

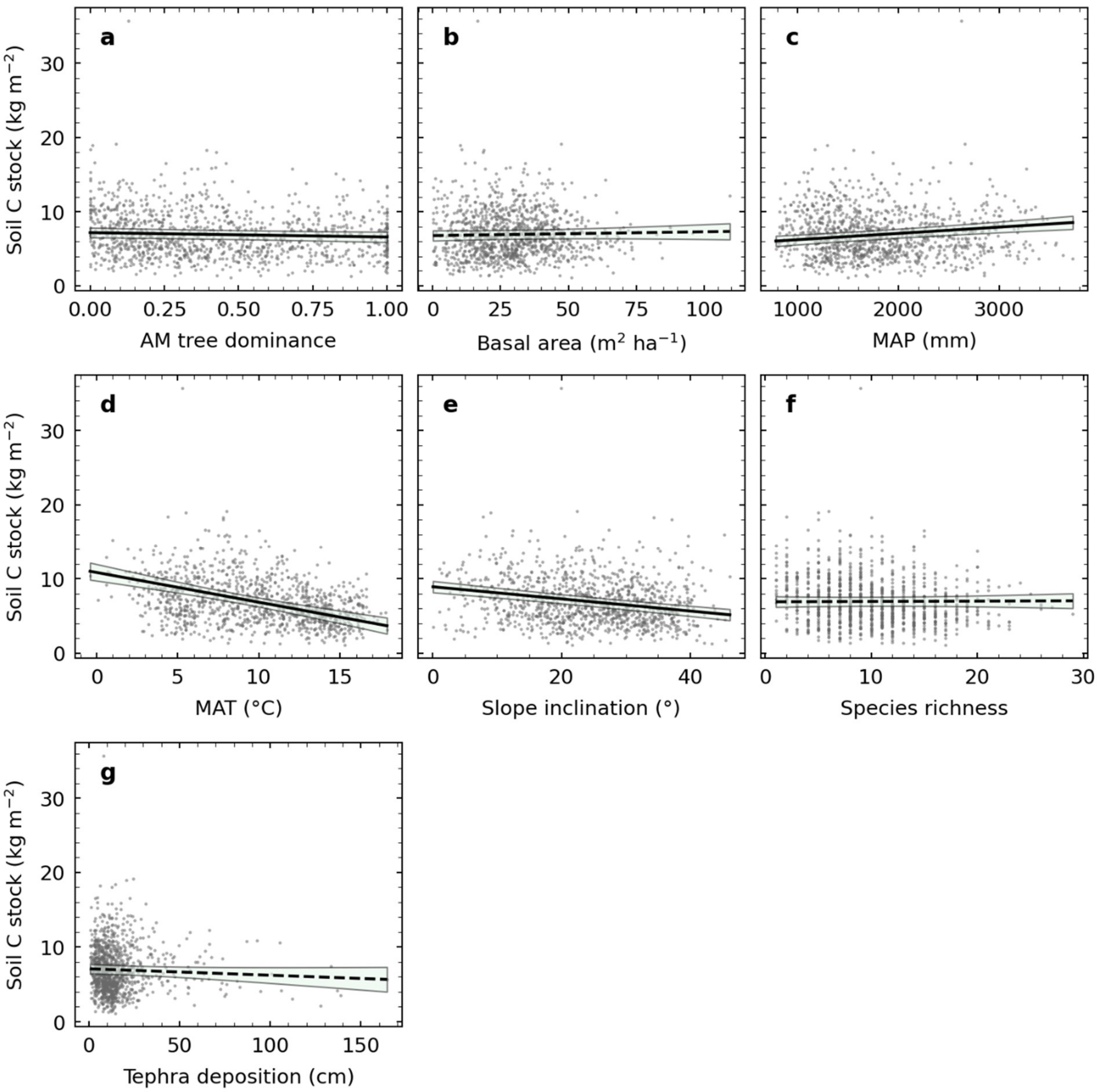

Supplementary Figure 13 | Marginal effect predictions for generalized linear model (GLM) 5 with sample size n = 1094 plots. Marginal effect predictions are shown for (**a**) arbuscular mycorrhizal (AM) tree dominance, (**b**) basal area, (**c**) mean annual precipitation (MAP), (**d**) mean annual temperature (MAT), (**e**) slope inclination, (**f**) species richness, and (**g**) tephra deposition. Marginal effect predictions (lines) are shown with 95% confidence intervals (shades). Marginal effect predictions of insignificant predictors ($p < 0.05$; Wald Z-test, two-sided) have dashed lines. Exact p values for predictor effects are provided in Supplementary Data 1. Grey dots show the underlying data. Shade colors correspond to the mean AM tree dominance of the analyzed plots (color legend in Fig. 1c). Source data are provided as a Source Data file.

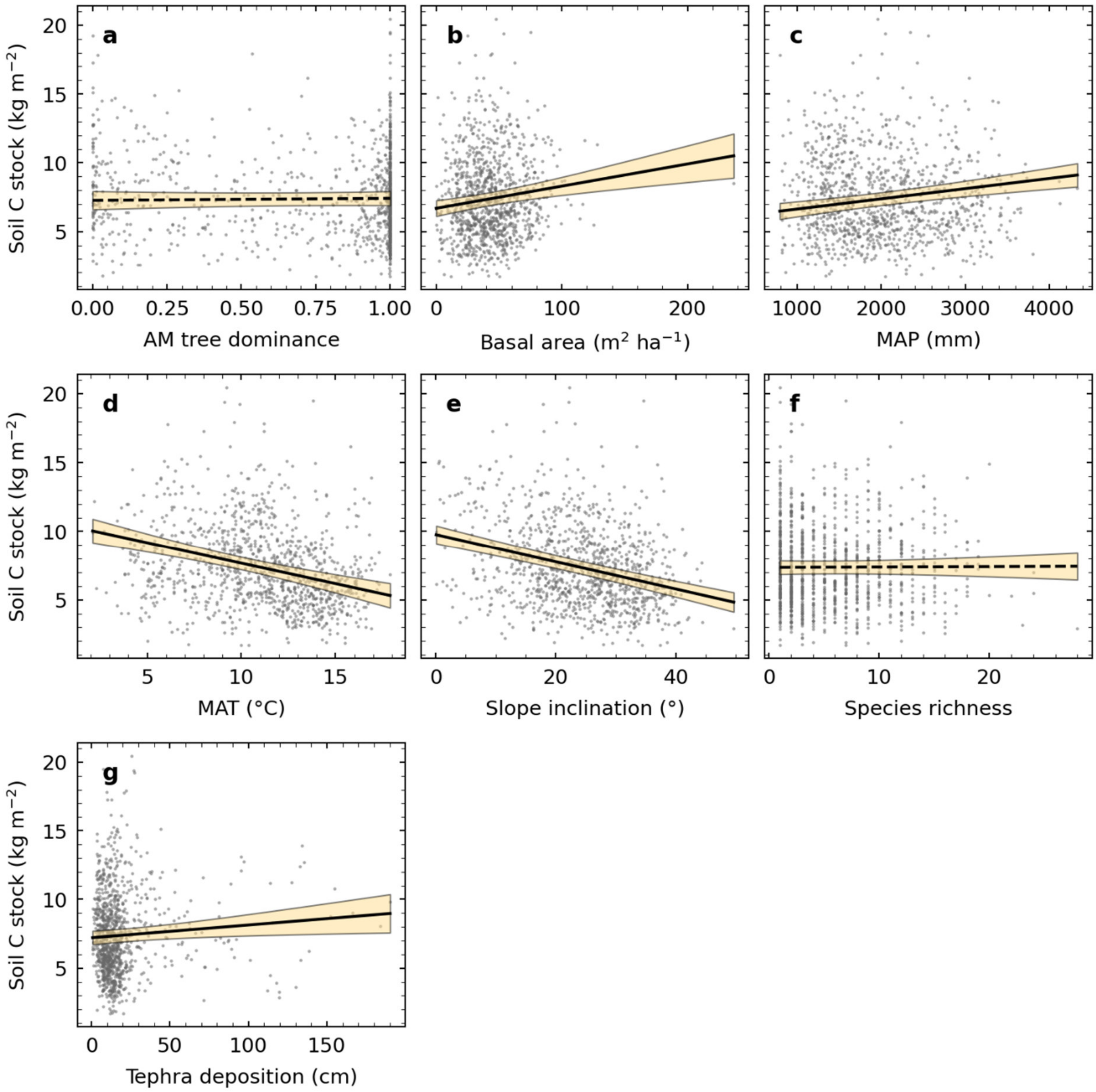

Supplementary Figure 14 | Marginal effect predictions for generalized linear model (GLM) 6 with sample size n = 1068 plots. Marginal effect predictions are shown for (**a**) arbuscular mycorrhizal (AM) tree dominance, (**b**) basal area, (**c**) mean annual precipitation (MAP), (**d**) mean annual temperature (MAT), (**e**) slope inclination, (**f**) species richness, and (**g**) tephra deposition. Marginal effect predictions (lines) are shown with 95% confidence intervals (shades). Marginal effect predictions of insignificant predictors ($p < 0.05$; Wald Z-test, two-sided) have dashed lines. Exact p values for predictor effects are provided in Supplementary Data 1. Grey dots show the underlying data. Shade colors correspond to the mean AM tree dominance of the analyzed plots (color legend in Fig. 1c). Source data are provided as a Source Data file.

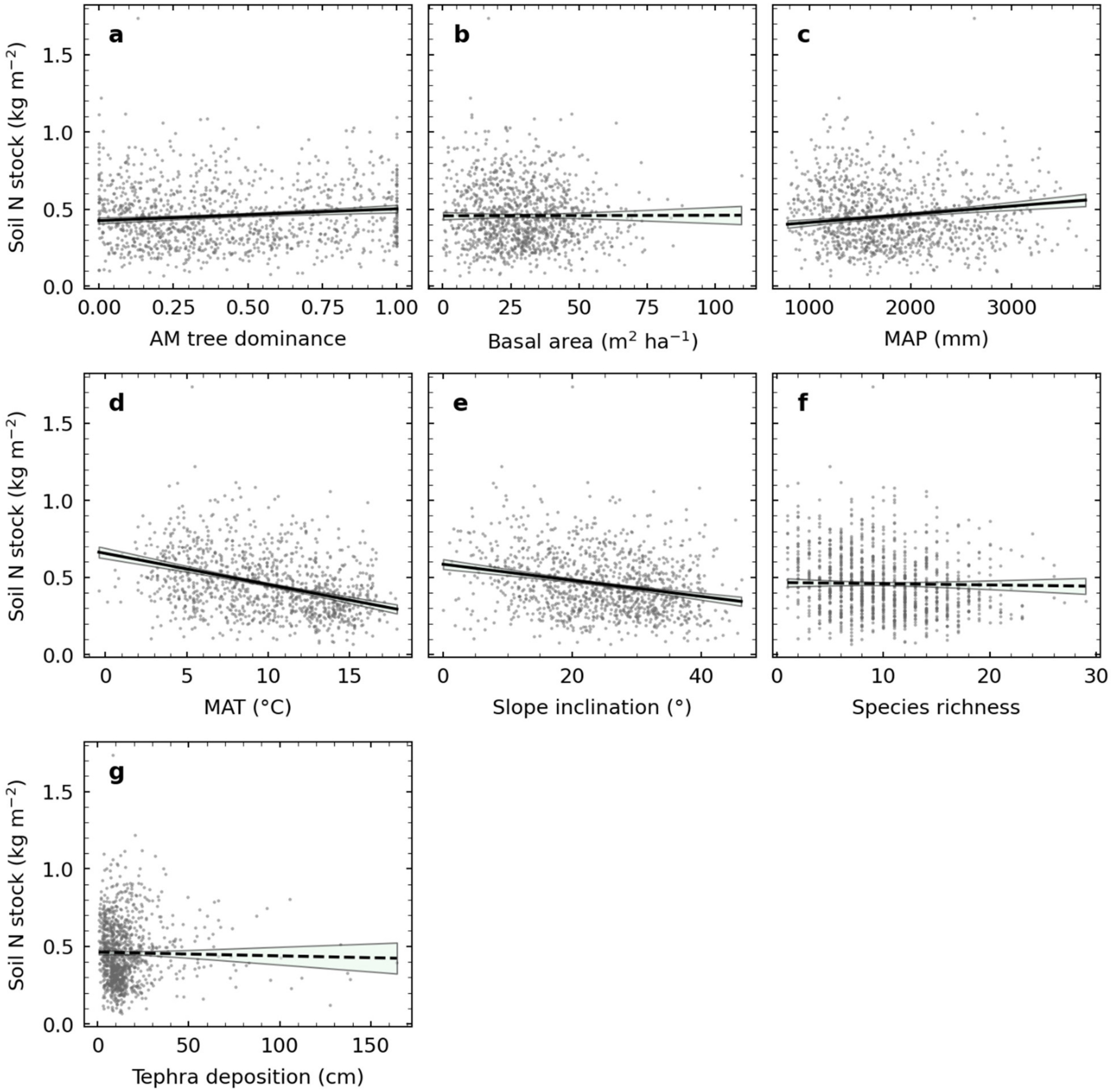

Supplementary Figure 15 | Marginal effect predictions for generalized linear model (GLM) 7 with sample size n = 1094 plots. Marginal effect predictions are shown for (**a**) arbuscular mycorrhizal (AM) tree dominance, (**b**) basal area, (**c**) mean annual precipitation (MAP), (**d**) mean annual temperature (MAT), (**e**) slope inclination, (**f**) species richness, and (**g**) tephra deposition. Marginal effect predictions (lines) are shown with 95% confidence intervals (shades). Marginal effect predictions of insignificant predictors ($p < 0.05$; Wald Z-test, two-sided) have dashed lines. Exact p values for predictor effects are provided in Supplementary Data 1. Grey dots show the underlying data. Shade colors correspond to the mean AM tree dominance of the analyzed plots (color legend in Fig. 1c). Source data are provided as a Source Data file.

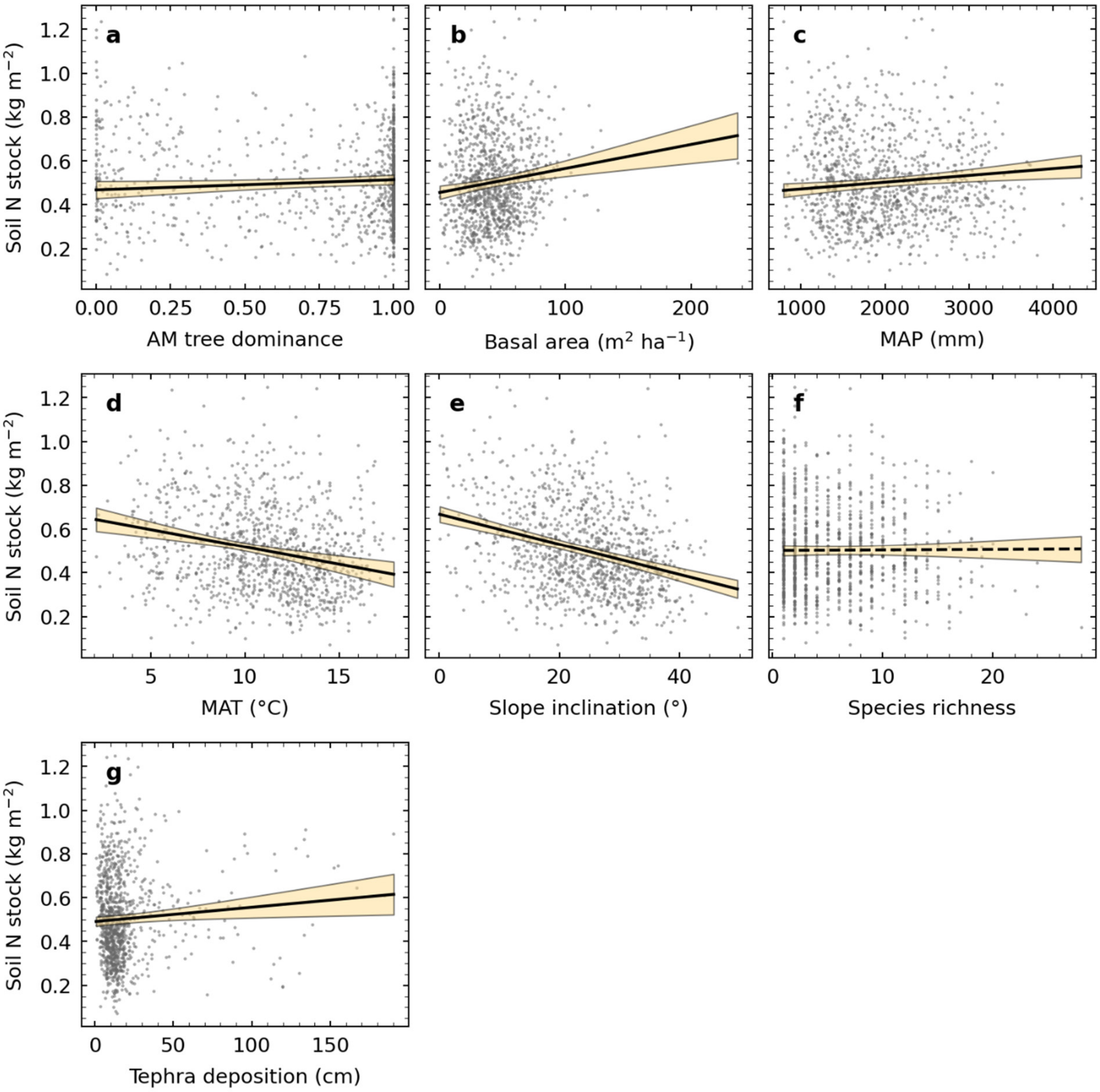

Supplementary Figure 16 | Marginal effect predictions for generalized linear model (GLM) 8 with sample size n = 1068 plots. Marginal effect predictions are shown for (**a**) arbuscular mycorrhizal (AM) tree dominance, (**b**) basal area, (**c**) mean annual precipitation (MAP), (**d**) mean annual temperature (MAT), (**e**) slope inclination, (**f**) species richness, and (**g**) tephra deposition. Marginal effect predictions (lines) are shown with 95% confidence intervals (shades). Marginal effect predictions of insignificant predictors ($p < 0.05$; Wald Z-test, two-sided) have dashed lines. Exact p values for predictor effects are provided in Supplementary Data 1. Grey dots show the underlying data. Shade colors correspond to the mean AM tree dominance of the analyzed plots (color legend in Fig. 1c). Source data are provided as a Source Data file.

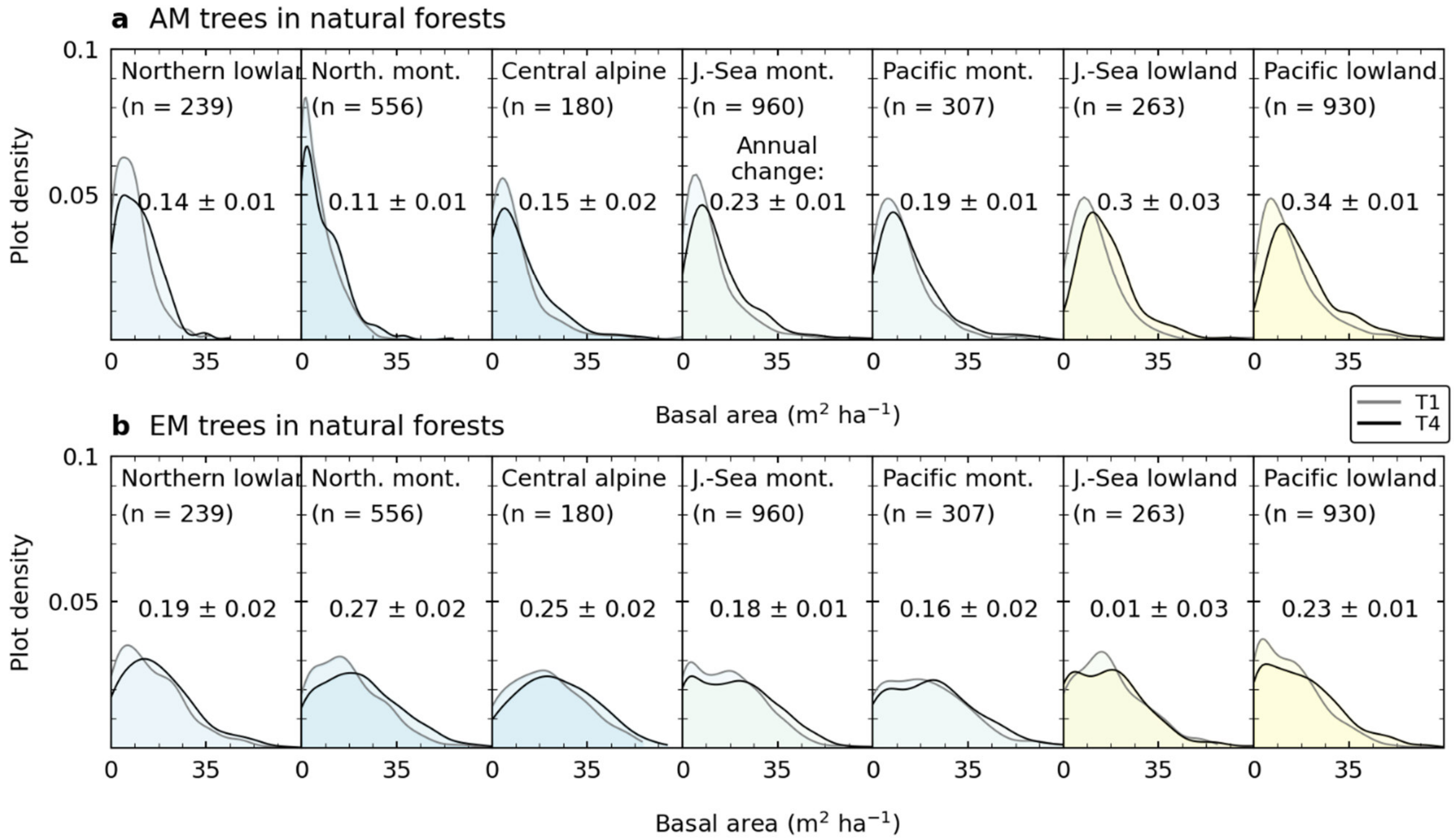

Supplementary Figure 17 | Change in the basal area distributions of AM and EM trees in natural forest plots between inventory campaigns T1 (1999–2004) and T4 (2014–2019). a Plot density by AM tree basal area in campaigns T1 and T4. **b** Plot density by EM tree basal area in campaigns T1 and T4. The number of plots that were compared between campaigns is indicated below each ecoregion name. For each ecoregion the mean annual change with standard errors is shown. The color under each curve corresponds to the mean AM tree dominance of the analyzed plot population (color legend in Fig. 1c). Source data are provided as a Source Data file.

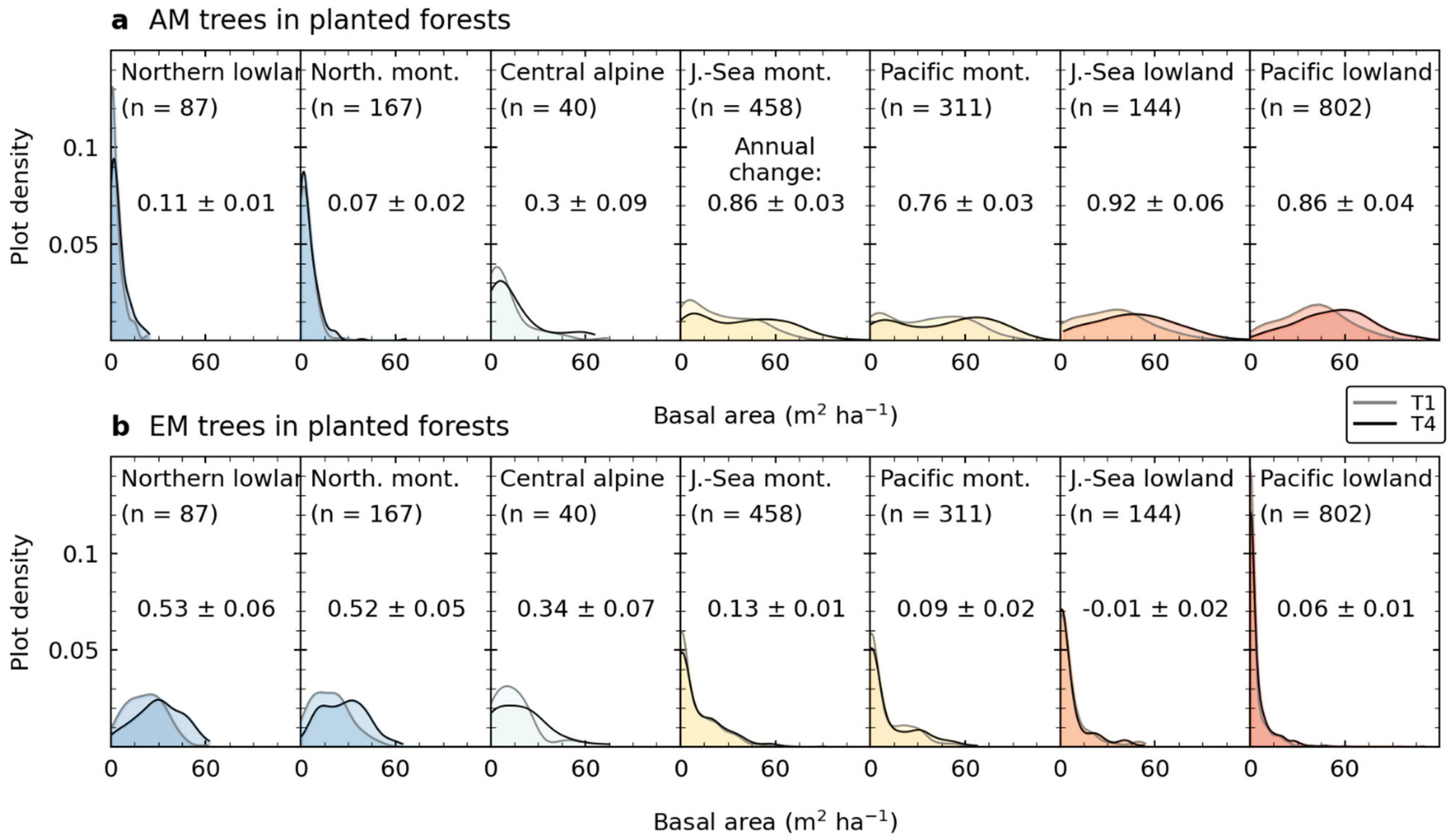

Supplementary Figure 18 | Change in the basal area distributions of AM and EM trees in planted forest plots between inventory campaigns T1 (1999–2004) and T4 (2014–2019). a Plot density by AM tree basal area in campaigns T1 and T4. **b** Plot density by EM tree basal area in campaigns T1 and T4. The number of plots that were compared between campaigns is indicated below each ecoregion name. For each ecoregion the mean annual change with standard errors is shown. The color under each curve corresponds to the mean AM tree dominance of the analyzed plot population (color legend in Fig. 1c). Source data are provided as a Source Data file.

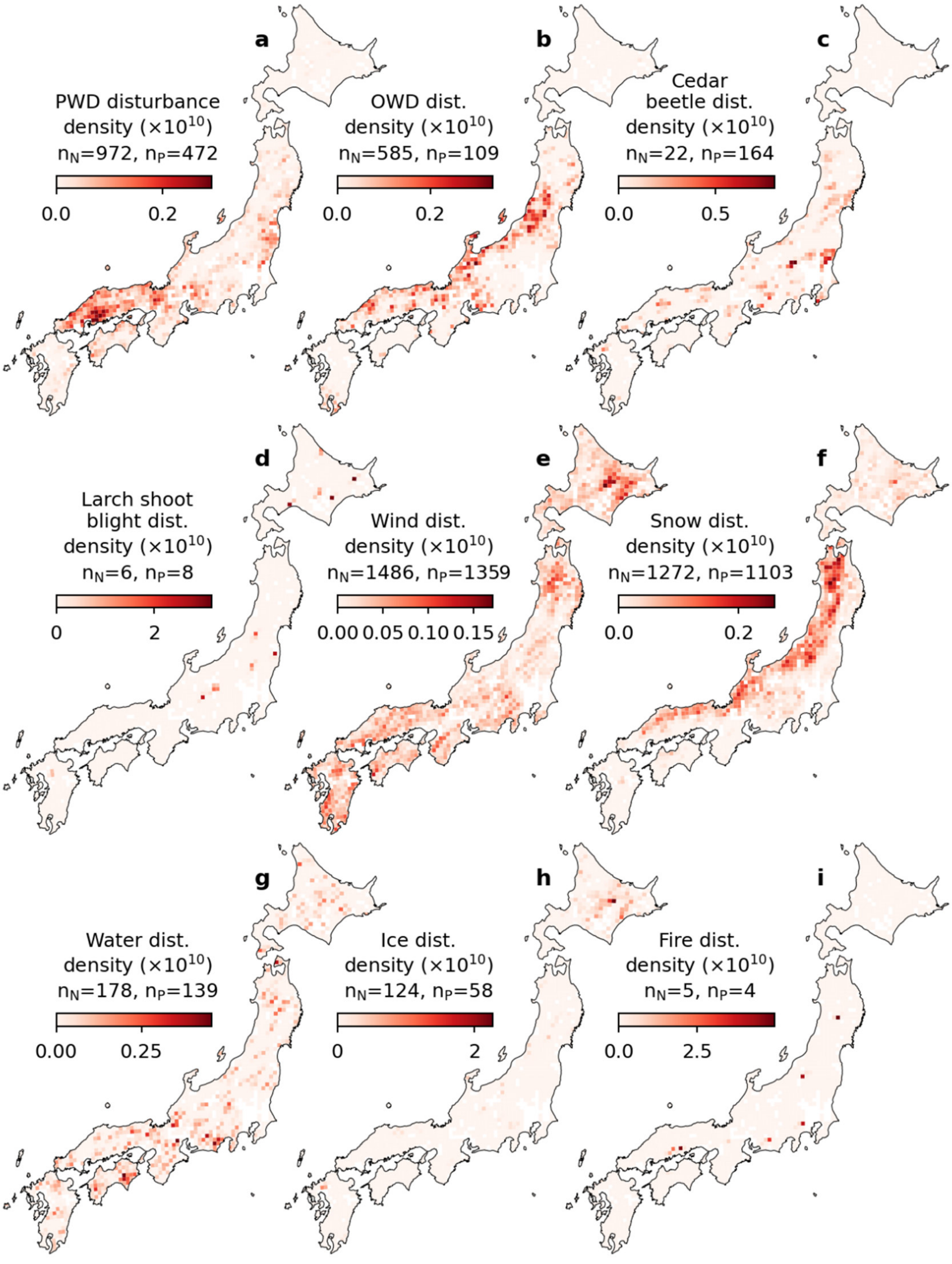

Supplementary Figure 19 | Density distributions of the most common forest disturbance types estimated using kernel density estimation. Density distributions are shown for disturbances from (**a**) pine wilt disease (PWD), (**b**) oak wilt disease (OWD), (**c**) the Japanese cedar long-horned beetle (*Callidiellum rufipenne*), (**d**) larch shoot blight caused by the fungus *Neofusicoccum laricinum*, (**e**) wind, (**f**) snow, (**g**) water, (**h**) ice, and (**i**) fire. n_N and n_P are the numbers of natural and planted forest plots, respectively, where the respective disturbance had been recorded in any of the four National Forest Inventory (NFI) campaigns. Coastlines were drawn based on the total extent of all temperate ecoregions of Japan after Olson et al. (2001)[66,67,69] using data from RESOLVE[68].

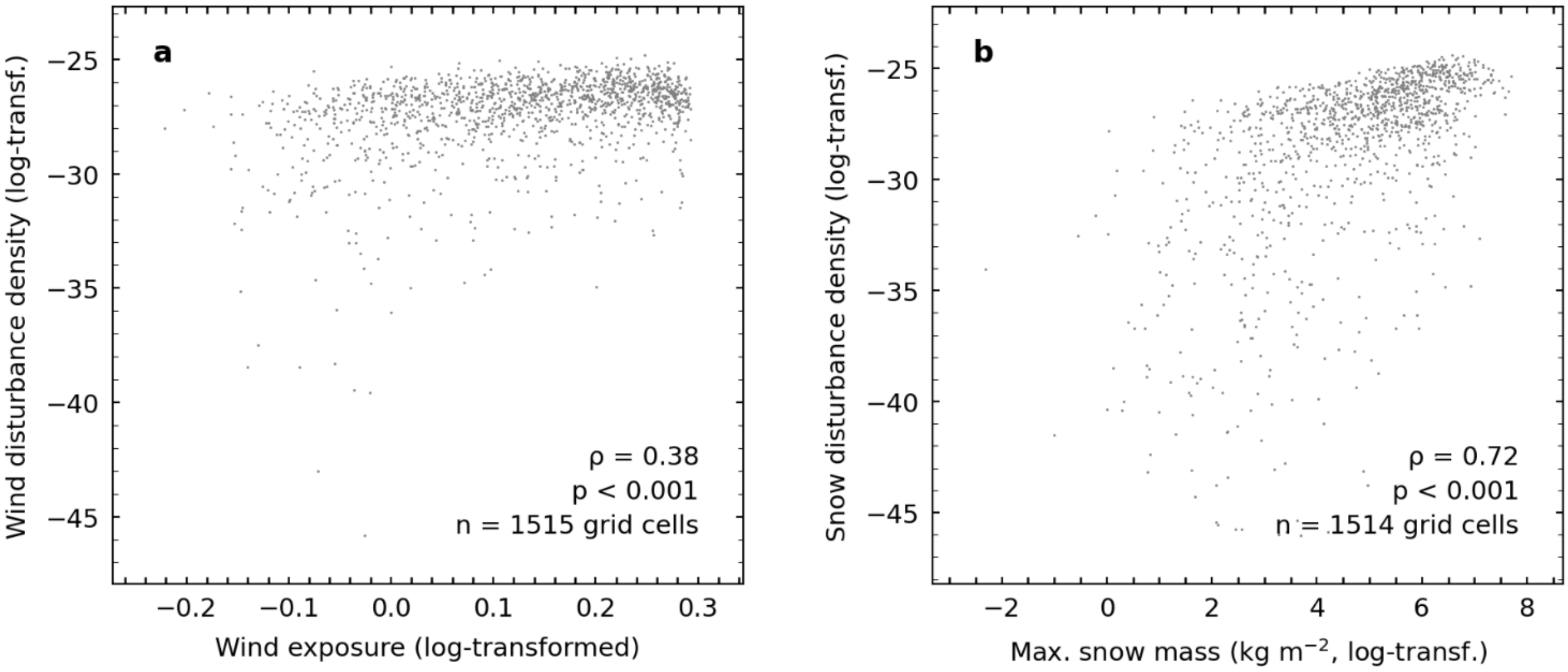

Supplementary Figure 20 | Correlations between abiotic disturbance variables and the respective disturbance density distribution across forest grid cells. a Correlation between wind exposure and wind disturbance density. **b** Correlation between maximum snow mass and snow disturbance density. Correlations were tested using Spearman's rank correlation (one-sided). Correlations were tested for grid cells containing at least one plot. Plots show log-transformed data. Exact p values are (**a**) 1.11×10^{-52} and (**b**) 1.03×10^{-239}. Logarithms of extremely small values ($< 10^{-20}$) beyond the plot limits are contained in the source data file. Source data are provided as a Source Data file.

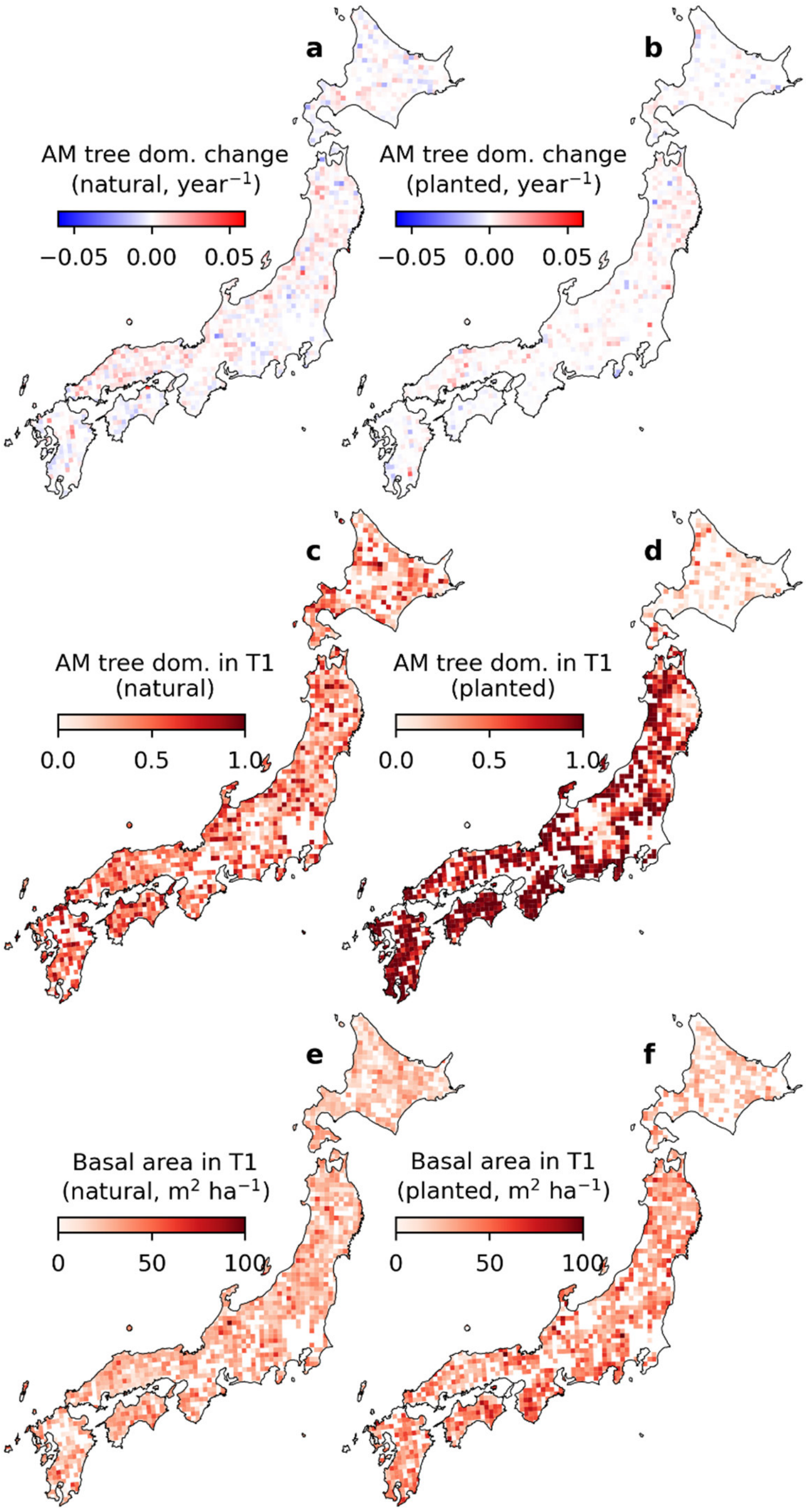

Supplementary Figure 21 | Distributions of predictors and dependent variables in generalized linear models (GLMs) 11 and 12 among forest grid cells. Distributions among natural (n = 1234) and planted forest grid cells (n = 978) are shown for (**a, b**) the arbuscular mycorrhizal (AM) tree dominance change between in inventory campaigns T1 and T4, (**c, d**) the AM tree dominance in T1, and (**e, f**) the basal area in T1. Basal area values ≥100 m^2 ha^{-1} have the same color. Coastlines were drawn based on the total extent of all temperate ecoregions of Japan after Olson et al. (2001)[66,67,69] using data from RESOLVE[68].

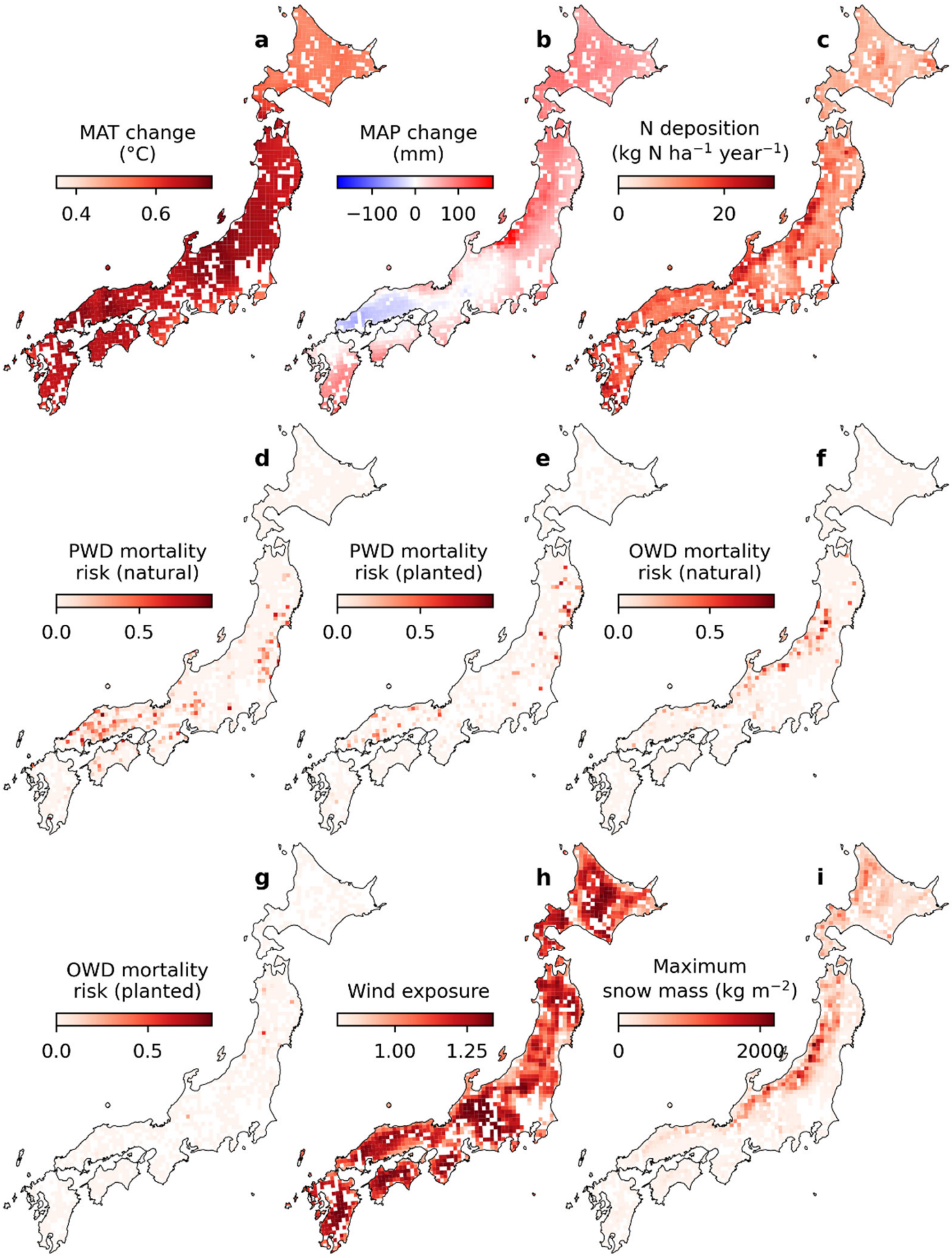

Supplementary Figure 22 | Distributions of predictors in generalized linear models (GLMs) 11 and 12 among forest grid cells. Distributions among natural (n = 1234) and planted forest grid cells (n = 978) are shown for (**a**) mean annual temperature (MAT) change, (**b**) mean annual precipitation (MAP) change, (**c**) N deposition, (**d, e**) pine wilt disease (PWD) mortality risk, (**f, g**) oak wilt disease (OWD) mortality risk, (**h**) wind exposure, and (**i**) maximum snow mass. Coastlines were drawn based on the total extent of all temperate ecoregions of Japan after Olson et al. (2001)[66,67,69] using data from RESOLVE[68].

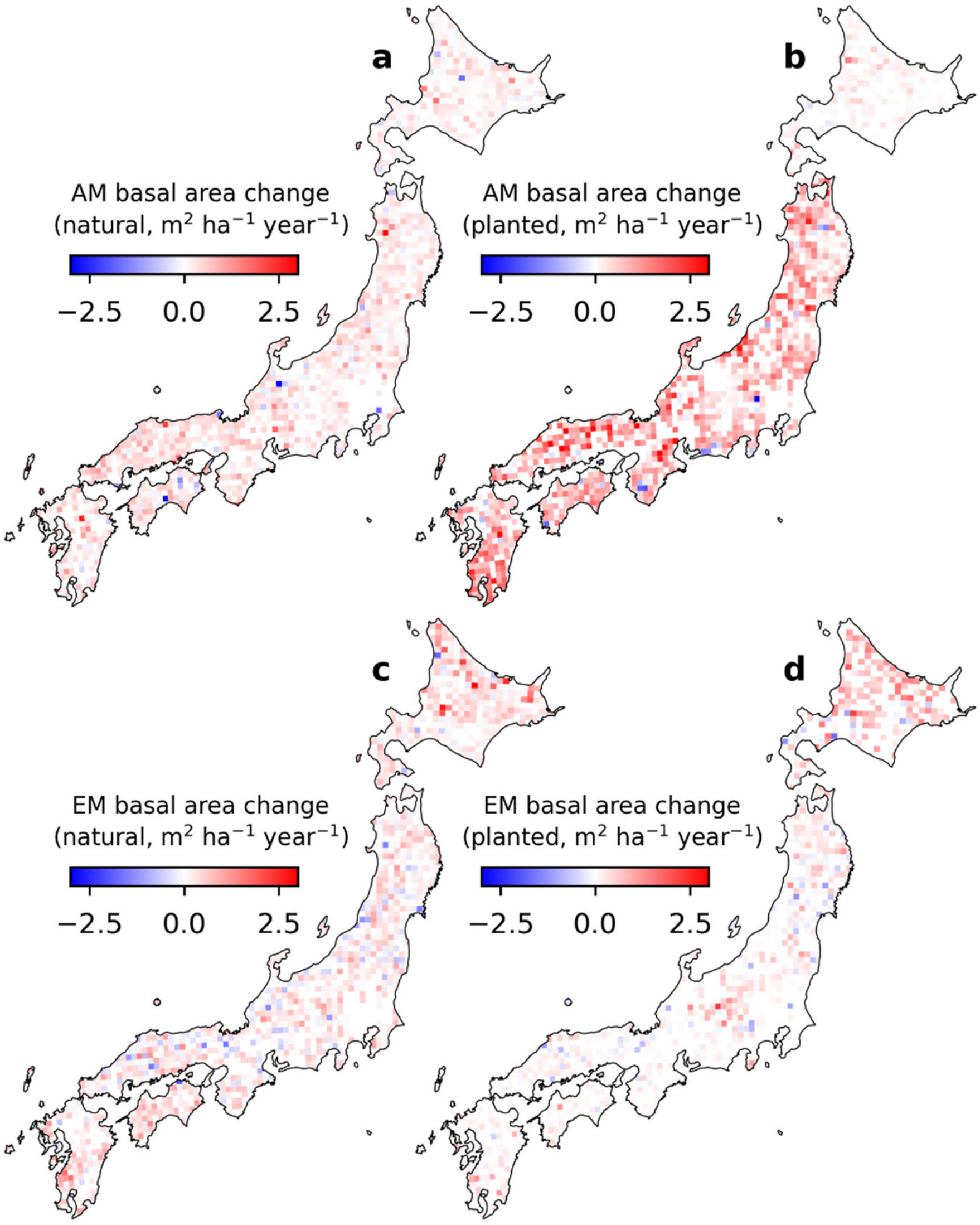

Supplementary Figure 23 | Distributions of the basal area changes of mycorrhizal trees in natural (n = 1234) and planted forest grid cells (n = 978). a Basal area changes of arbuscular mycorrhizal (AM) trees. **b** Basal area changes of ectomycorrhizal (EM) trees. Values ≤-3 m^2 ha^{-1} $year^{-1}$ and ≥3 m^2 ha^{-1} $year^{-1}$ have the same color, respectively. Coastlines were drawn based on the total extent of all temperate ecoregions of Japan after Olson et al. (2001)[66,67,69] using data from RESOLVE[68].

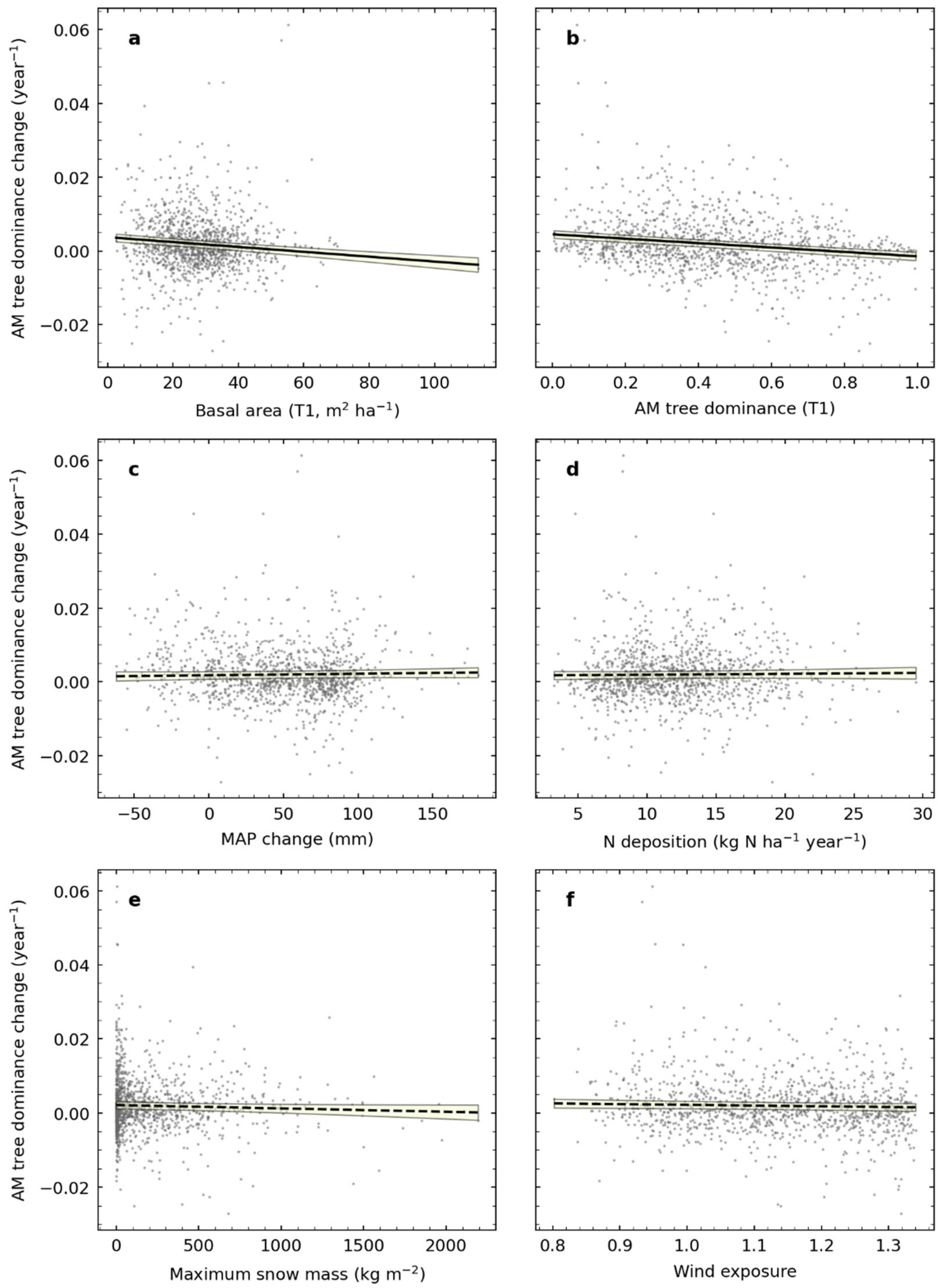

Supplementary Figure 24 | Marginal effect predictions for generalized linear model (GLM) 11 with sample size n = 1234 grid cells. Marginal effect predictions are shown for (**a**) basal area, (**b**) arbuscular mycorrhizal (AM) tree dominance, (**c**) mean annual precipitation (MAP) change, (**d**) N deposition, (**e**) maximum snow mass, and (**f**) wind exposure. Marginal effect predictions (lines) are shown with 95% confidence intervals (shades). Marginal effect predictions of insignificant predictors ($p < 0.05$; Wald Z-test, two-sided) have dashed lines. Exact p values for predictor effects are provided in Supplementary Data 1. Grey dots show the underlying data. Shade colors correspond to the mean AM tree dominance of the analyzed grid cells (color legend in Fig. 1c). Source data are provided as a Source Data file.

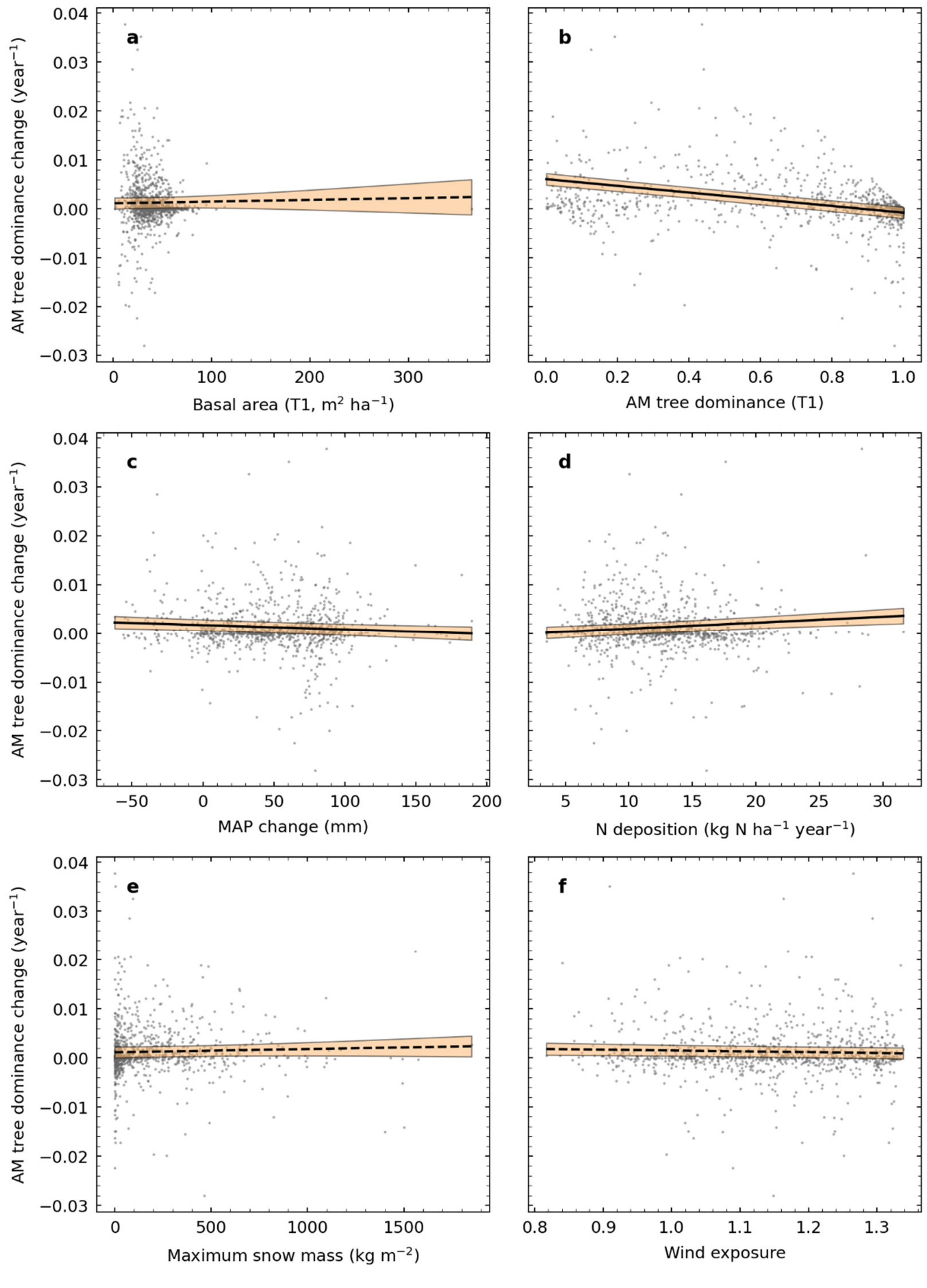

Supplementary Figure 25 | Marginal effect predictions for generalized linear model (GLM) 12 with sample size n = 978 grid cells. Marginal effect predictions are shown for (**a**) basal area, (**b**) arbuscular mycorrhizal (AM) tree dominance, (**c**) mean annual precipitation (MAP) change, (**d**) N deposition, (**e**) maximum snow mass, and (**f**) wind exposure. Marginal effect predictions (lines) are shown with 95% confidence intervals (shades). Marginal effect predictions of insignificant predictors ($p < 0.05$; Wald Z-test, two-sided) have dashed lines. Exact p values for predictor effects are provided in Supplementary Data 1. Grey dots show the underlying data. Shade colors correspond to the mean AM tree dominance of the analyzed grid cells (color legend in Fig. 1c). Source data are provided as a Source Data file.

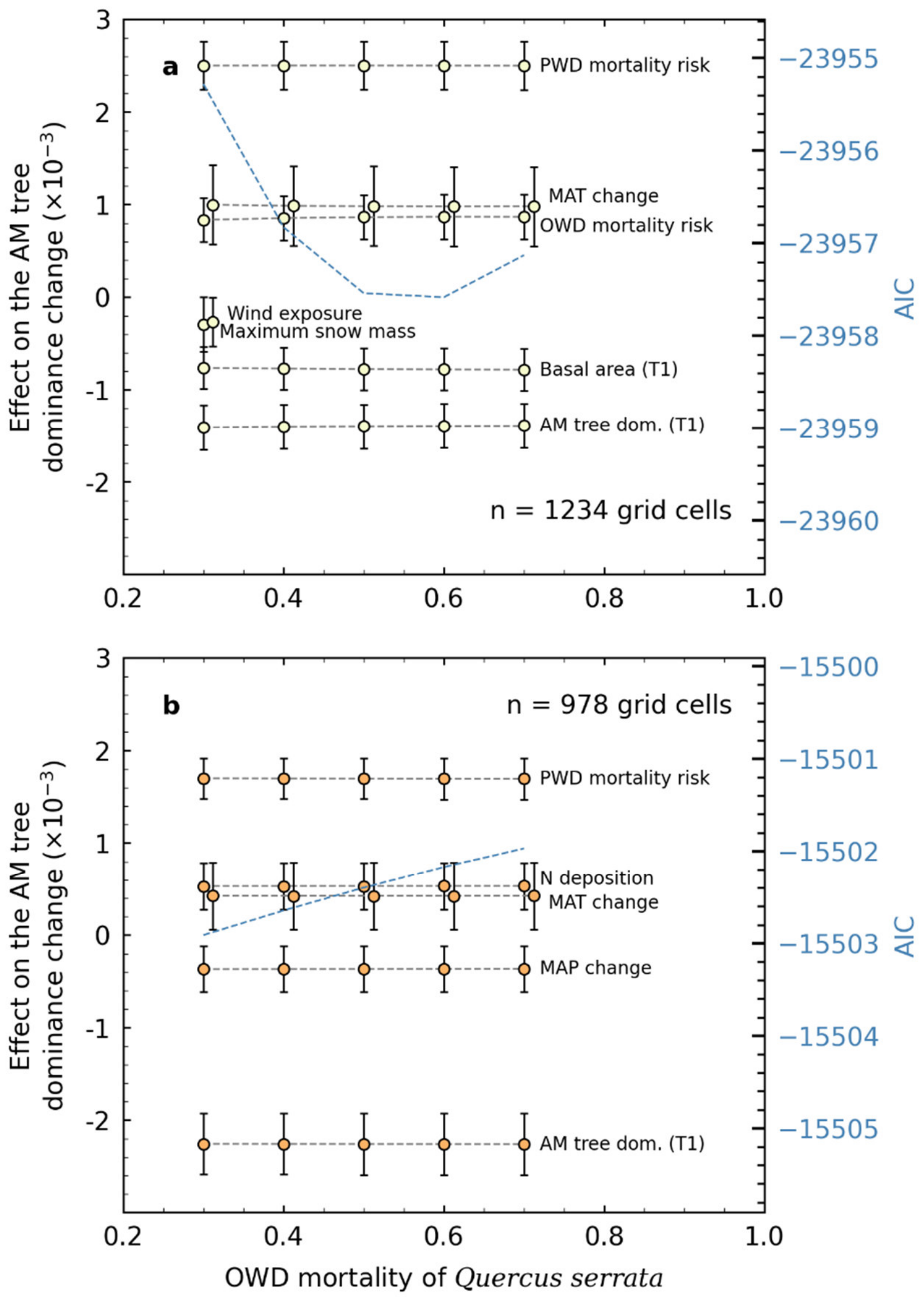

Supplementary Figure 26 | Predictors of the AM tree dominance change between inventory campaigns T1 and T4 for different OWD mortalities of *Quercus serrata*. a Effect sizes of significant predictors in natural forest grid cells in generalized linear model (GLM) 11. **b** Effect sizes of significant predictors in planted forest grid cells in GLM 12. Error bars indicate 95% confidence intervals. Exact p values for predictor effects are in the source data. Source data are provided as a Source Data file.

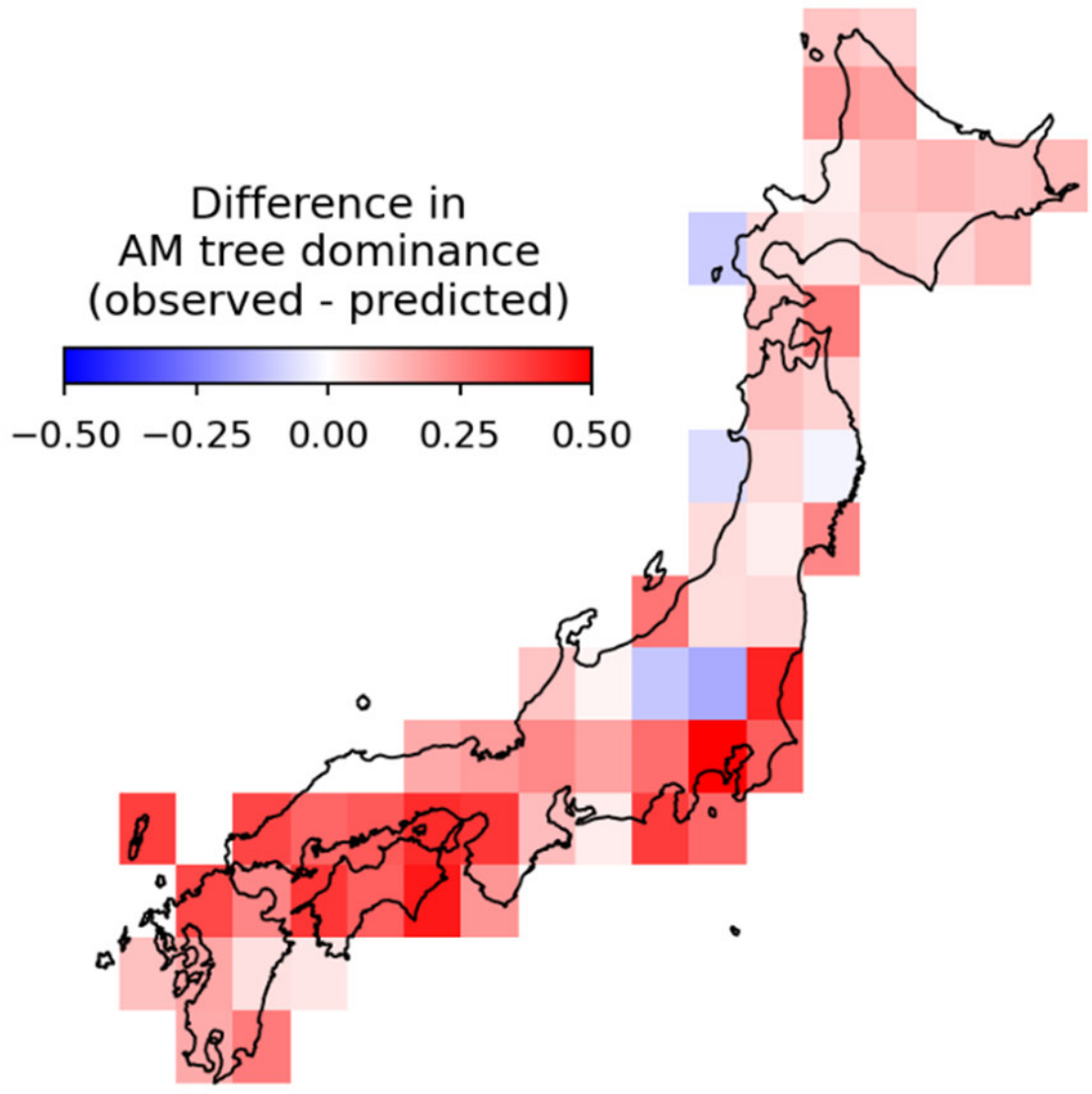

Supplementary Figure 27 | Difference between the AM tree dominance in T4 and the AM tree dominance predicted in a global-scale tree symbiosis map by Steidinger et al. (2019). Data from all National Forest Inventory (NFI) plots in temperate ecoregions in T4 were aggregated to a 1°-resolution grid of beta means (n = 62). The AM tree dominance predicted by Steidinger et al. (2019) was then subtracted in each grid cell. Coastlines were drawn based on the total extent of all temperate ecoregions of Japan after Olson et al. (2001)[66,67,69] using data from RESOLVE[68].

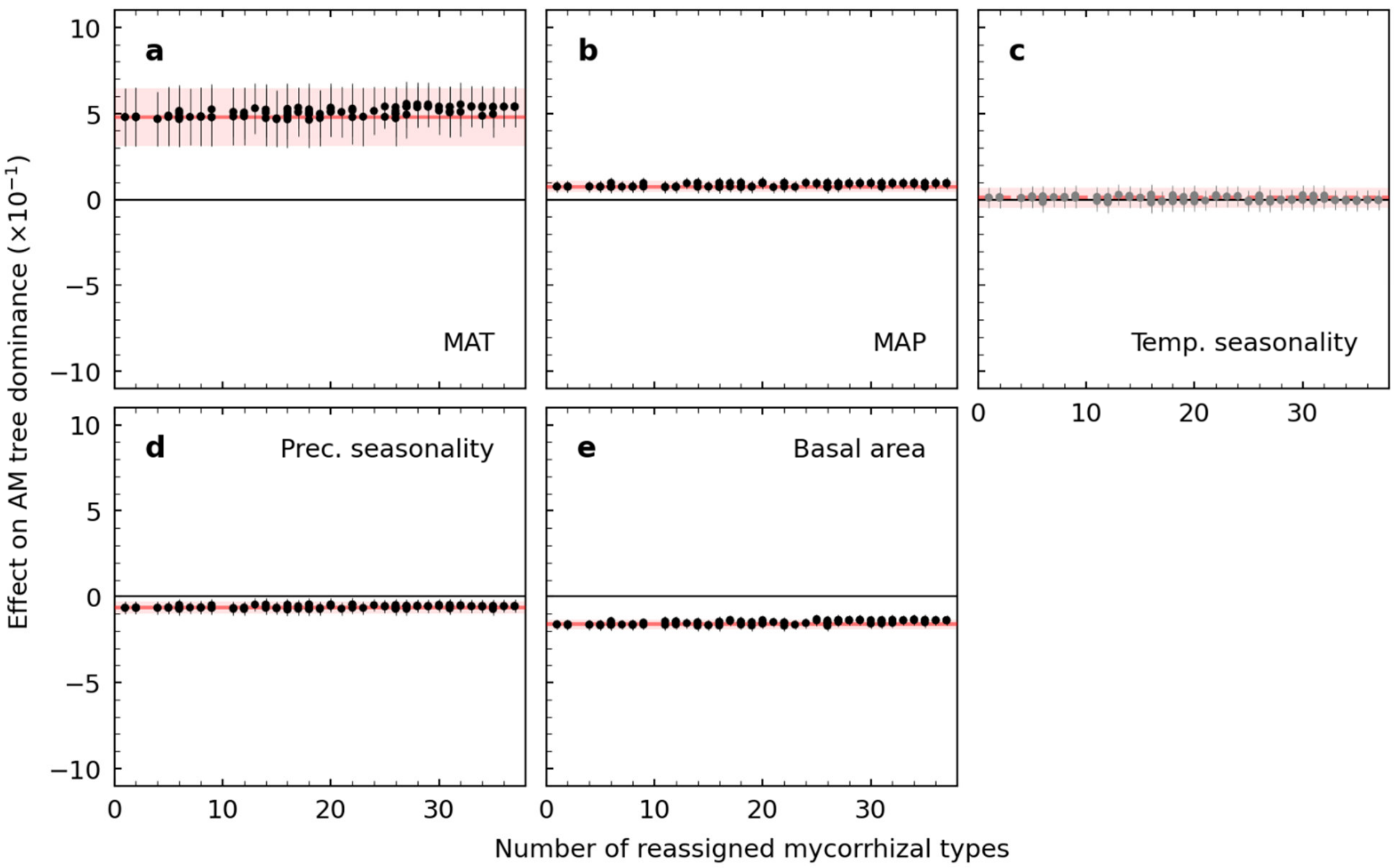

Supplementary Figure 28 | Predictor effect sizes in generalized linear model (GLM) 1 after reassigning mycorrhizal types (n = 100 iterations). Predictor effect sizes are shown for (**a**) mean annual temperature (MAT), (**b**) mean annual precipitation (MAP), (**c**) temperature seasonality, (**d**) precipitation seasonality, and (**e**) basal area. Significant ($p < 0.05$) and insignificant predictor effects are shown as black and grey dots, respectively (Wald Z-test, two-sided). Error bars indicate 95% confidence intervals. Predictor effect sizes in the main analysis (red line) with 95% confidence intervals (shadows) are shown for comparison, with solid and dashed lines representing significant ($p < 0.05$) and insignificant predictor effects, respectively (Wald Z-test, two-sided). Point coordinates and model information including exact p values are in the source data. Assigned mycorrhizal types are in Supplementary Data 2. Source data are provided as a Source Data file.

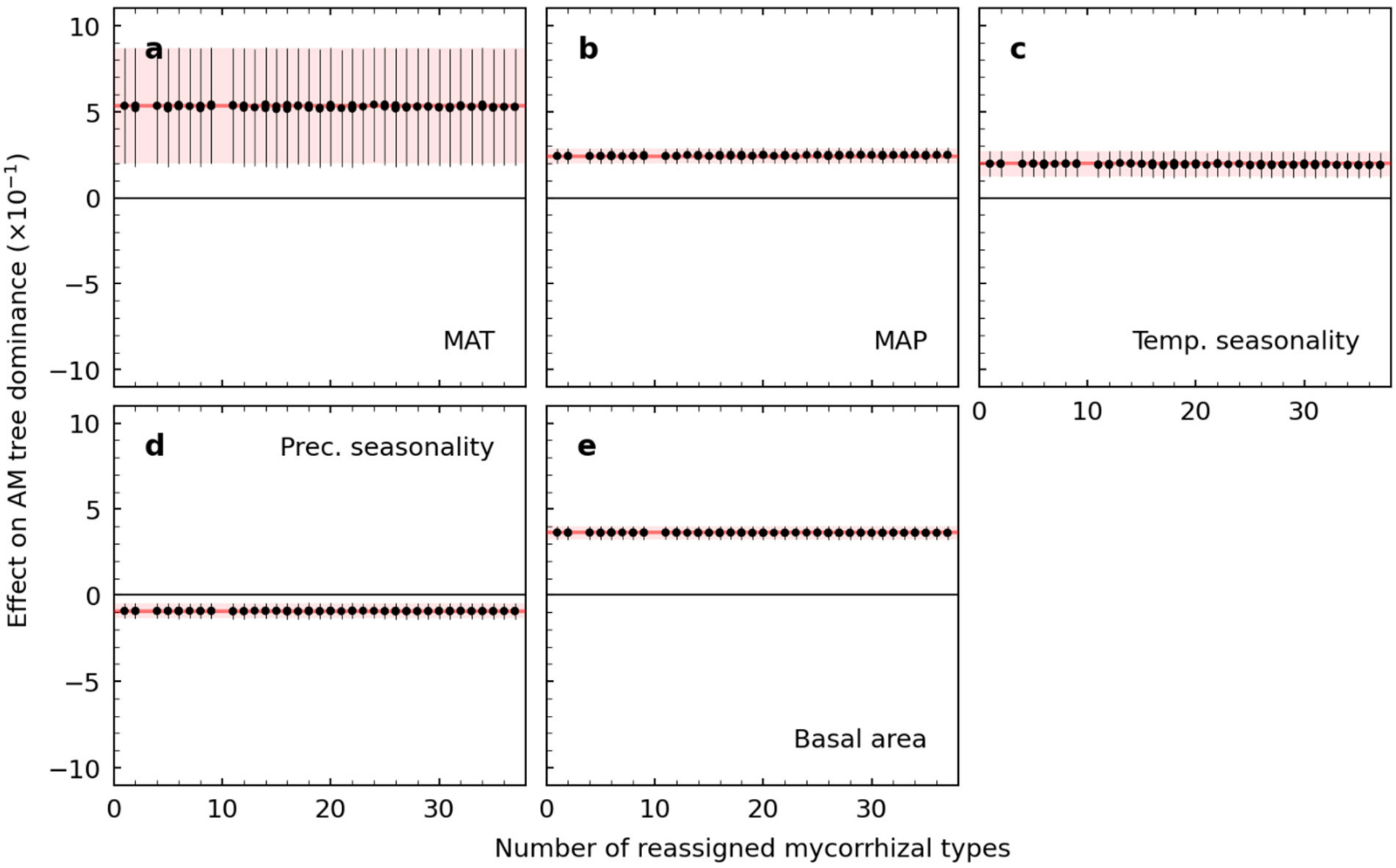

Supplementary Figure 29 | Predictor effect sizes in generalized linear model (GLM) 2 after reassigning mycorrhizal types (n = 100 iterations). Predictor effect sizes are shown for (**a**) mean annual temperature (MAT), (**b**) mean annual precipitation (MAP), (**c**) temperature seasonality, (**d**) precipitation seasonality, and (**e**) basal area. Significant ($p < 0.05$) and insignificant predictor effects are shown as black and grey dots, respectively (Wald Z-test, two-sided). Error bars indicate 95% confidence intervals. Predictor effect sizes in the main analysis (red line) with 95% confidence intervals (shadows) are shown for comparison, with solid and dashed lines representing significant ($p < 0.05$) and insignificant predictor effects, respectively (Wald Z-test, two-sided). Point coordinates and model information including exact p values are in the source data. Assigned mycorrhizal types are in Supplementary Data 2. Source data are provided as a Source Data file.

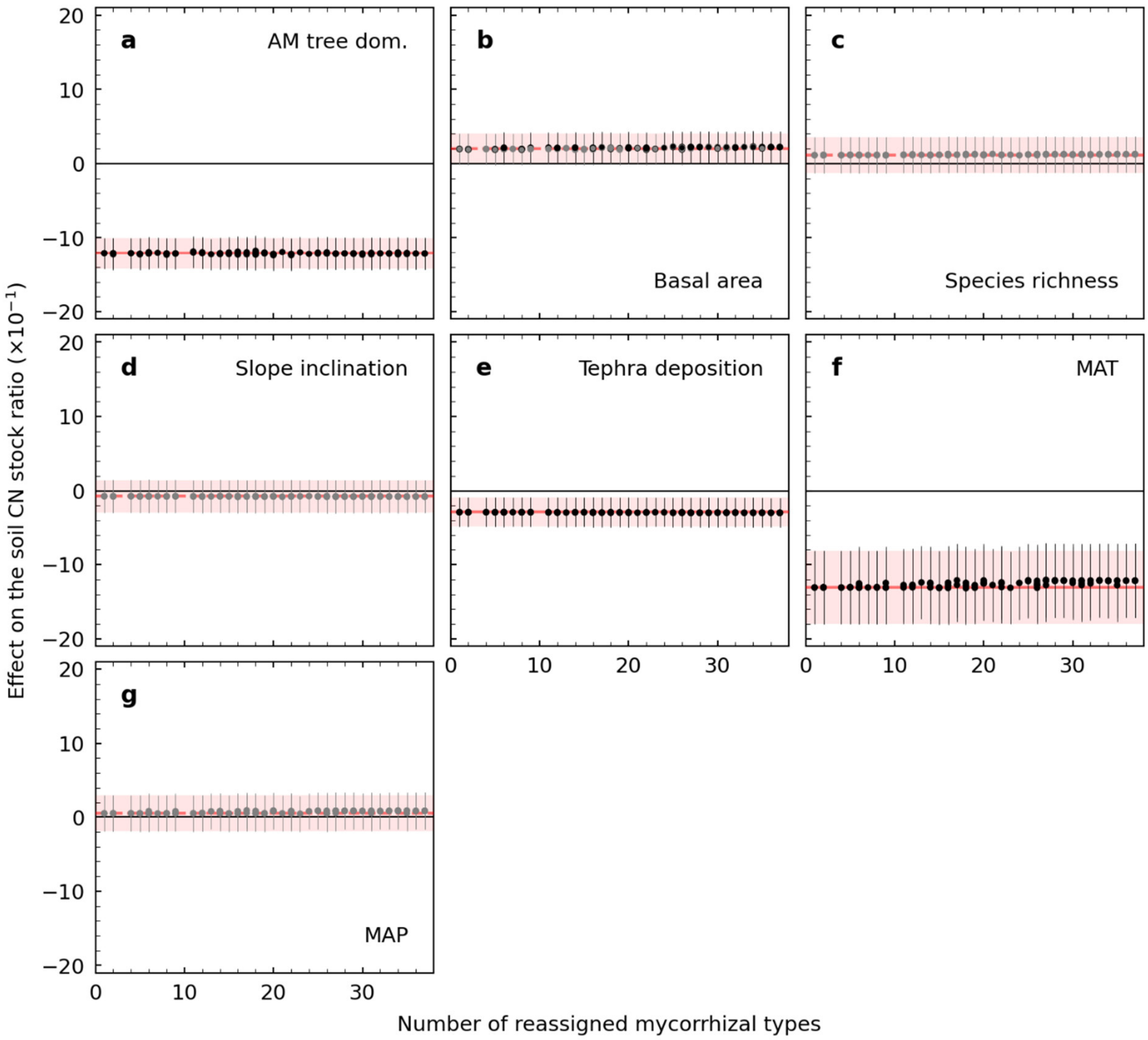

Supplementary Figure 30 | Predictor effect sizes in generalized linear model (GLM) 3 after reassigning mycorrhizal types (n = 100 iterations). Predictor effect sizes are shown for (**a**) arbuscular mycorrhizal (AM) tree dominance, (**b**) basal area, (**c**) species richness, (**d**) slope inclination, (**e**) tephra deposition, (**f**) mean annual temperature (MAT), and (**g**) mean annual precipitation (MAP). Significant ($p < 0.05$) and insignificant predictor effects are shown as black and grey dots, respectively (Wald Z-test, two-sided). Error bars indicate 95% confidence intervals. Predictor effect sizes in the main analysis (red line) with 95% confidence intervals (shadows) are shown for comparison, with solid and dashed lines representing significant ($p < 0.05$) and insignificant predictor effects, respectively (Wald Z-test, two-sided). Point coordinates and model information including exact p values are in the source data. Assigned mycorrhizal types are in Supplementary Data 2. Source data are provided as a Source Data file.

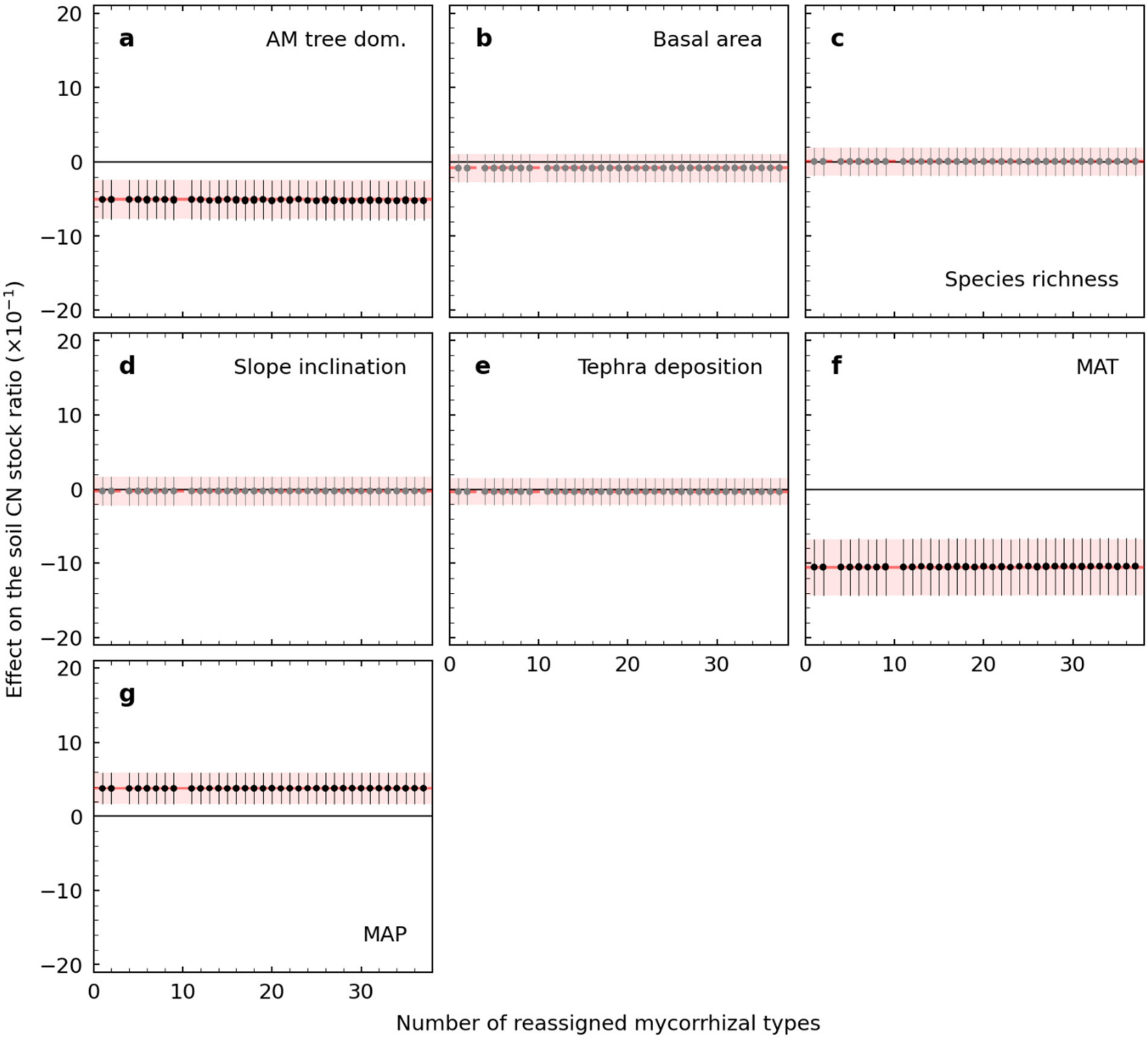

Supplementary Figure 31 | Predictor effect sizes in generalized linear model (GLM) 4 after reassigning mycorrhizal types (n = 100 iterations). Predictor effect sizes are shown for (**a**) arbuscular mycorrhizal (AM) tree dominance, (**b**) basal area, (**c**) species richness, (**d**) slope inclination, (**e**) tephra deposition, (**f**) mean annual temperature (MAT), and (**g**) mean annual precipitation (MAP). Significant ($p < 0.05$) and insignificant predictor effects are shown as black and grey dots, respectively (Wald Z-test, two-sided). Error bars indicate 95% confidence intervals. Predictor effect sizes in the main analysis (red line) with 95% confidence intervals (shadows) are shown for comparison, with solid and dashed lines representing significant ($p < 0.05$) and insignificant predictor effects, respectively (Wald Z-test, two-sided). Point coordinates and model information including exact p values are in the source data. Assigned mycorrhizal types are in Supplementary Data 2. Source data are provided as a Source Data file.

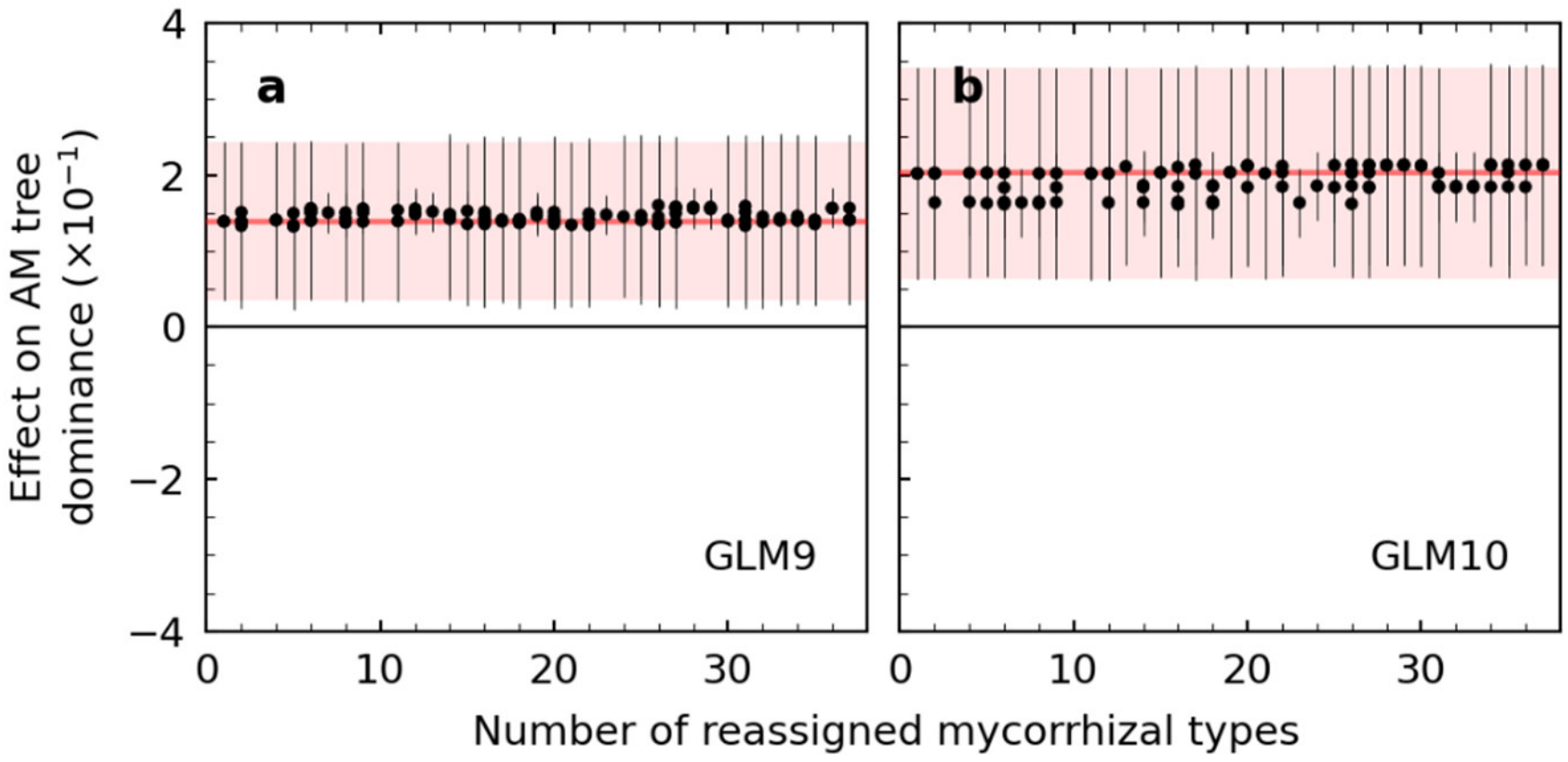

Supplementary Figure 32 | Campaign effect size on arbuscular mycorrhizal (AM) tree dominance after reassigning mycorrhizal types (n = 100 iterations). a Campaign effect size in generalized linear model (GLM) 9. **b** Campaign effect size in GLM 10. Significant ($p < 0.05$) and insignificant predictor effects are shown as black and grey dots, respectively (Wald Z-test, two-sided). Error bars indicate 95% confidence intervals. Predictor effect sizes in the main analysis (red line) with 95% confidence intervals (shadows) are shown for comparison, with solid and dashed lines representing significant ($p < 0.05$) and insignificant predictor effects, respectively (Wald Z-test, two-sided). Point coordinates and model information including exact p values are in the source data. Assigned mycorrhizal types are in Supplementary Data 2. Source data are provided as a Source Data file.

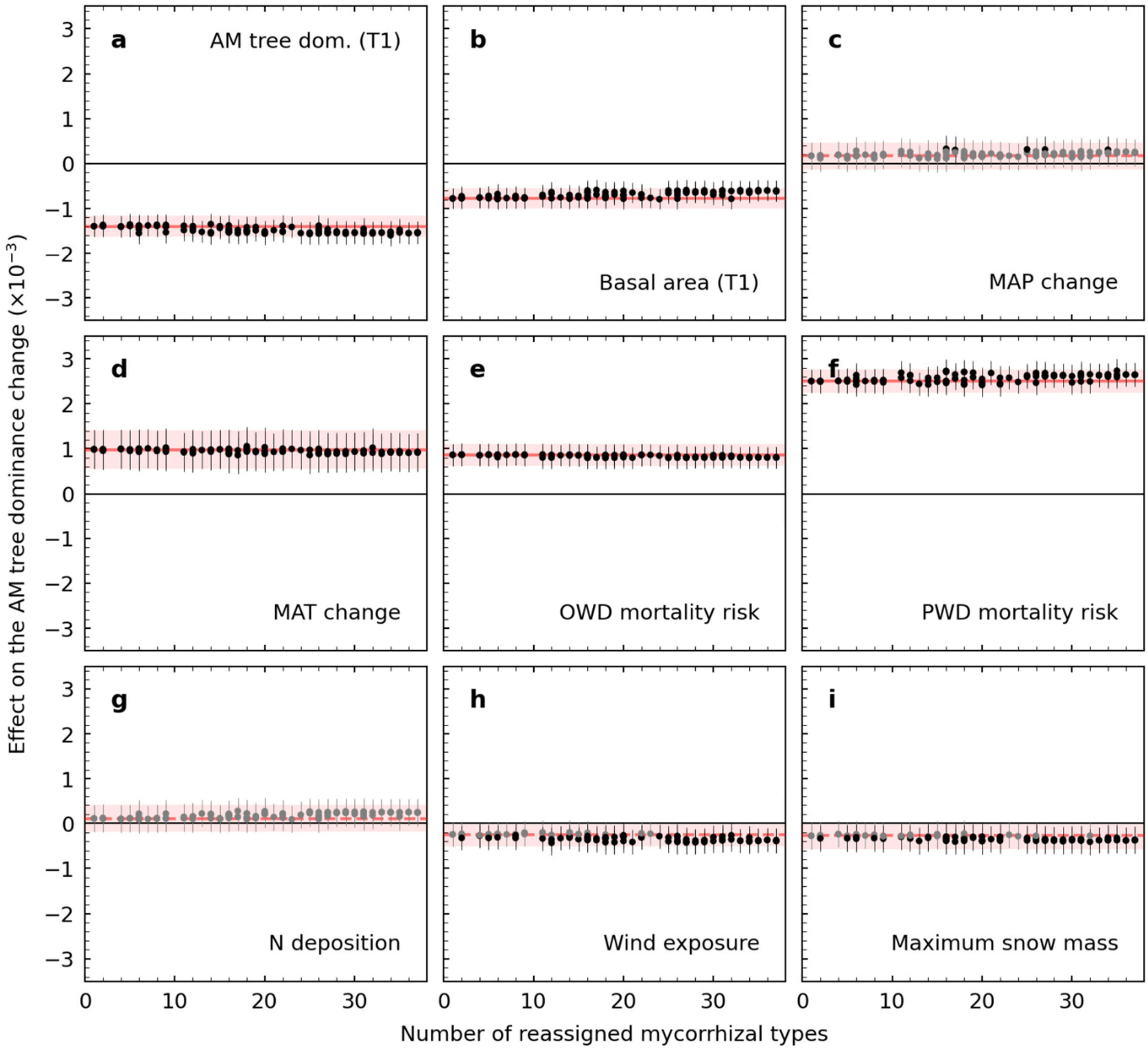

Supplementary Figure 33 | Predictor effect sizes in generalized linear model (GLM) 11 after reassigning mycorrhizal types (n = 100 iterations). Predictor effect sizes are shown for (**a**) arbuscular mycorrhizal (AM) tree dominance in T1, (**b**) basal area in T1, (**c**) mean annual precipitation (MAP) change, (**d**) mean annual temperature (MAT) change, (**e**) oak wilt disease (OWD) mortality risk, (**f**) pine wilt disease (PWD) mortality risk, (**g**) N deposition, (**h**) wind exposure, and (**i**) maximum snow mass. Significant ($p < 0.05$) and insignificant predictor effects are shown as black and grey dots, respectively (Wald Z-test, two-sided). Error bars indicate 95% confidence intervals. Predictor effect sizes in the main analysis (red line) with 95% confidence intervals (shadows) are shown for comparison, with solid and dashed lines representing significant ($p < 0.05$) and insignificant predictor effects, respectively (Wald Z-test, two-sided). Point coordinates and model information including exact p values are in the source data. Assigned mycorrhizal types are in Supplementary Data 2. Source data are provided as a Source Data file.

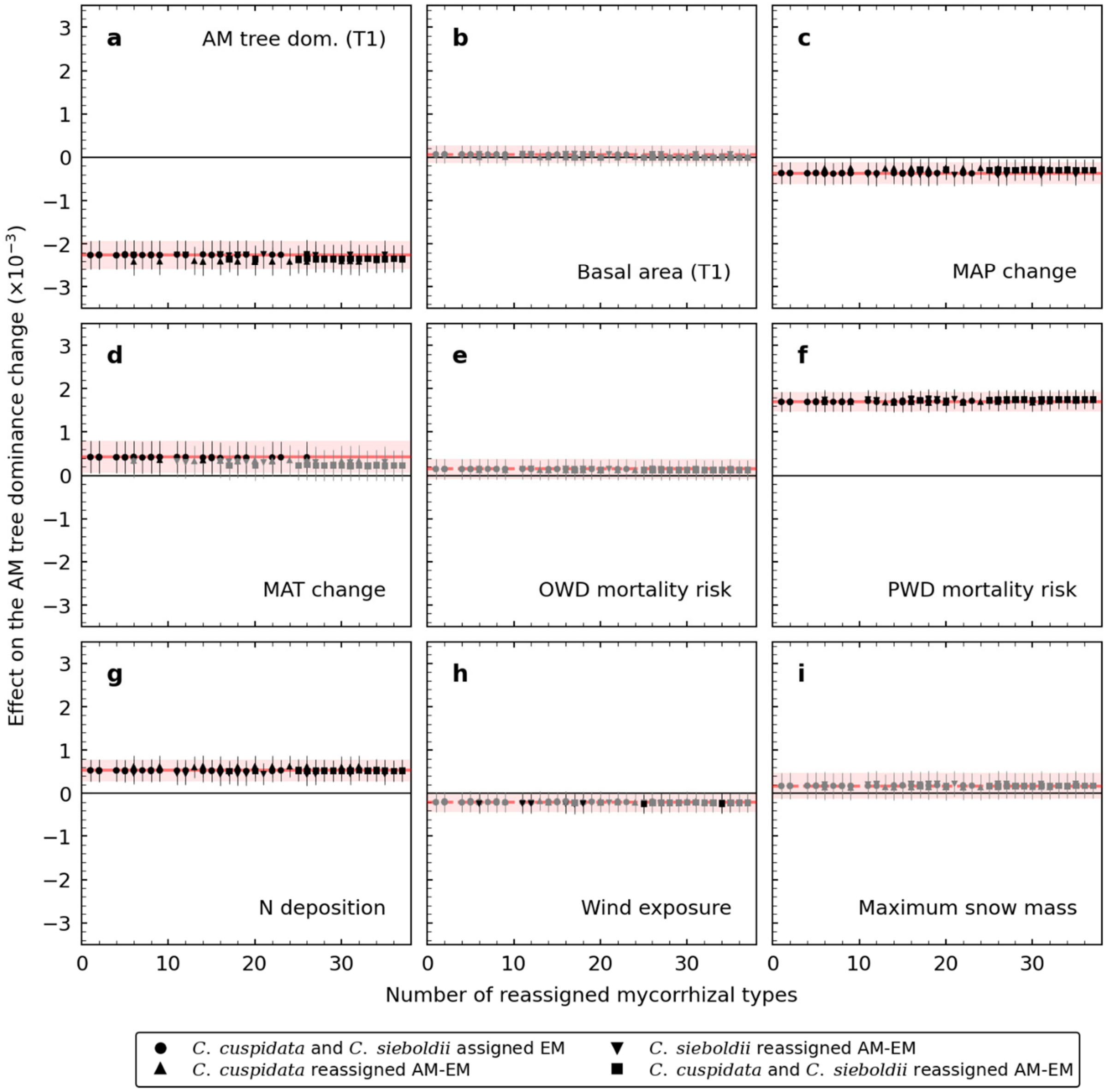

Supplementary Figure 34 | Predictor effect sizes in generalized linear model (GLM) 12 after reassigning mycorrhizal types (n = 100 iterations). Predictor effect sizes are shown for (**a**) arbuscular mycorrhizal (AM) tree dominance in T1, (**b**) basal area in T1, (**c**) mean annual precipitation (MAP) change, (**d**) mean annual temperature (MAT) change, (**e**) oak wilt disease (OWD) mortality risk, (**f**) pine wilt disease (PWD) mortality risk, (**g**) N deposition, (**h**) wind exposure, and (**i**) maximum snow mass. Significant ($p < 0.05$) and insignificant predictor effects are shown as black and grey dots, respectively (Wald Z-test, two-sided). Error bars indicate standard errors. Mycorrhizal type assignments of *Castanopsis* species are indicated with different markers. Predictor effect sizes in the main analysis (red line) with 95% confidence intervals (shadows) are shown for comparison, with solid and dashed lines representing significant ($p < 0.05$) and insignificant predictor effects, respectively (Wald Z-test, two-sided). Point coordinates and model information including exact p values are in the source data. Assigned mycorrhizal types are in Supplementary Data 2. Source data are provided as a Source Data file.

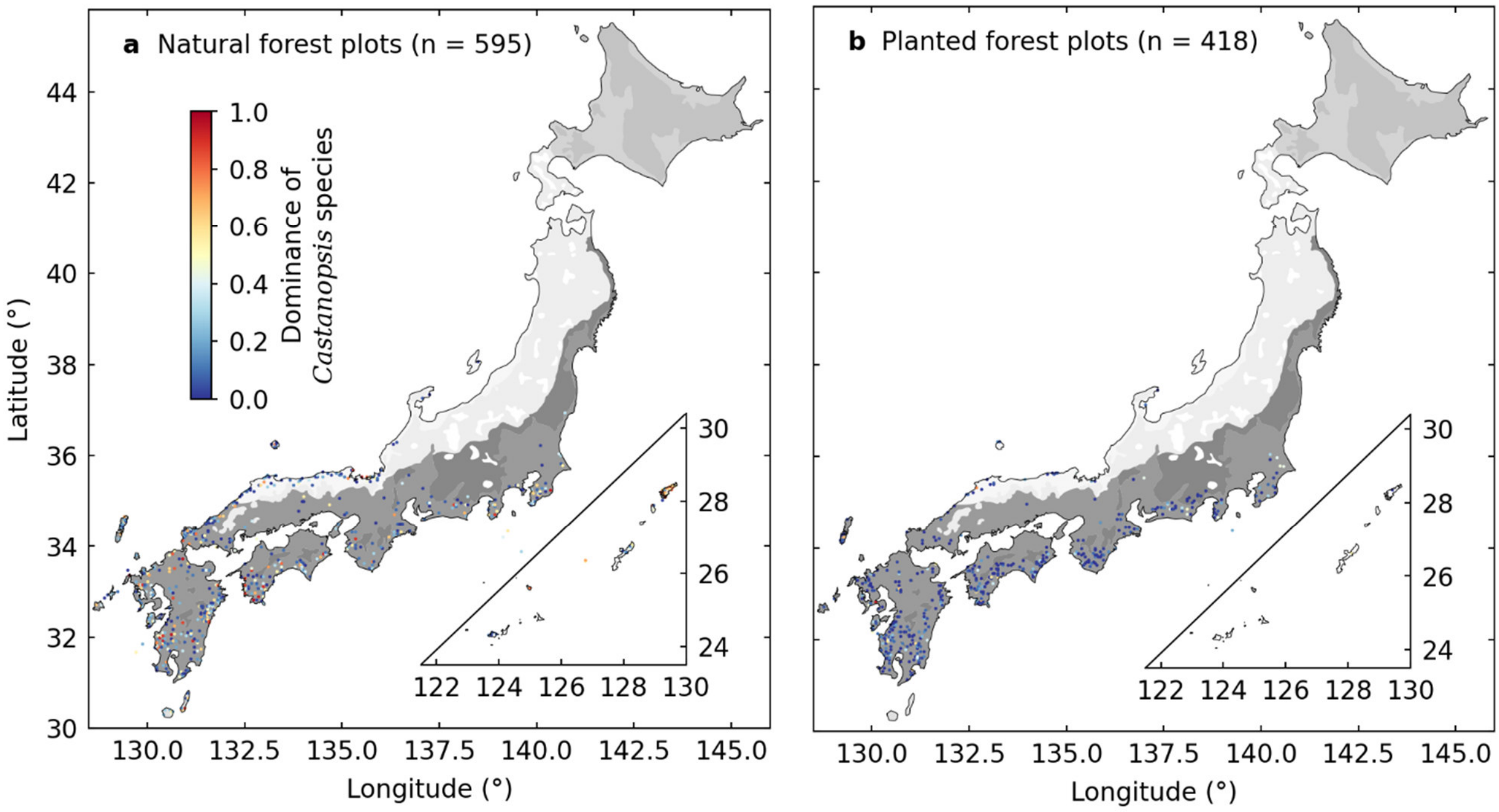

Supplementary Figure 35 | Dominance of *Castanopsis* tree species in natural and planted National Forest Inventory (NFI) plots. Species dominance is shown for (**a**) natural and (**b**) planted forest plots. Estimates are based on basal-area proportions in NFI campaign T4. Ecoregions of Japan after Olson et al. (2001)[66,67,69] are indicated in shades of grey. Ecoregions and coastlines were drawn based on data from RESOLVE[68]. See Figure 1 and Supplementary Tables 5–7 for more details on the ecoregions.

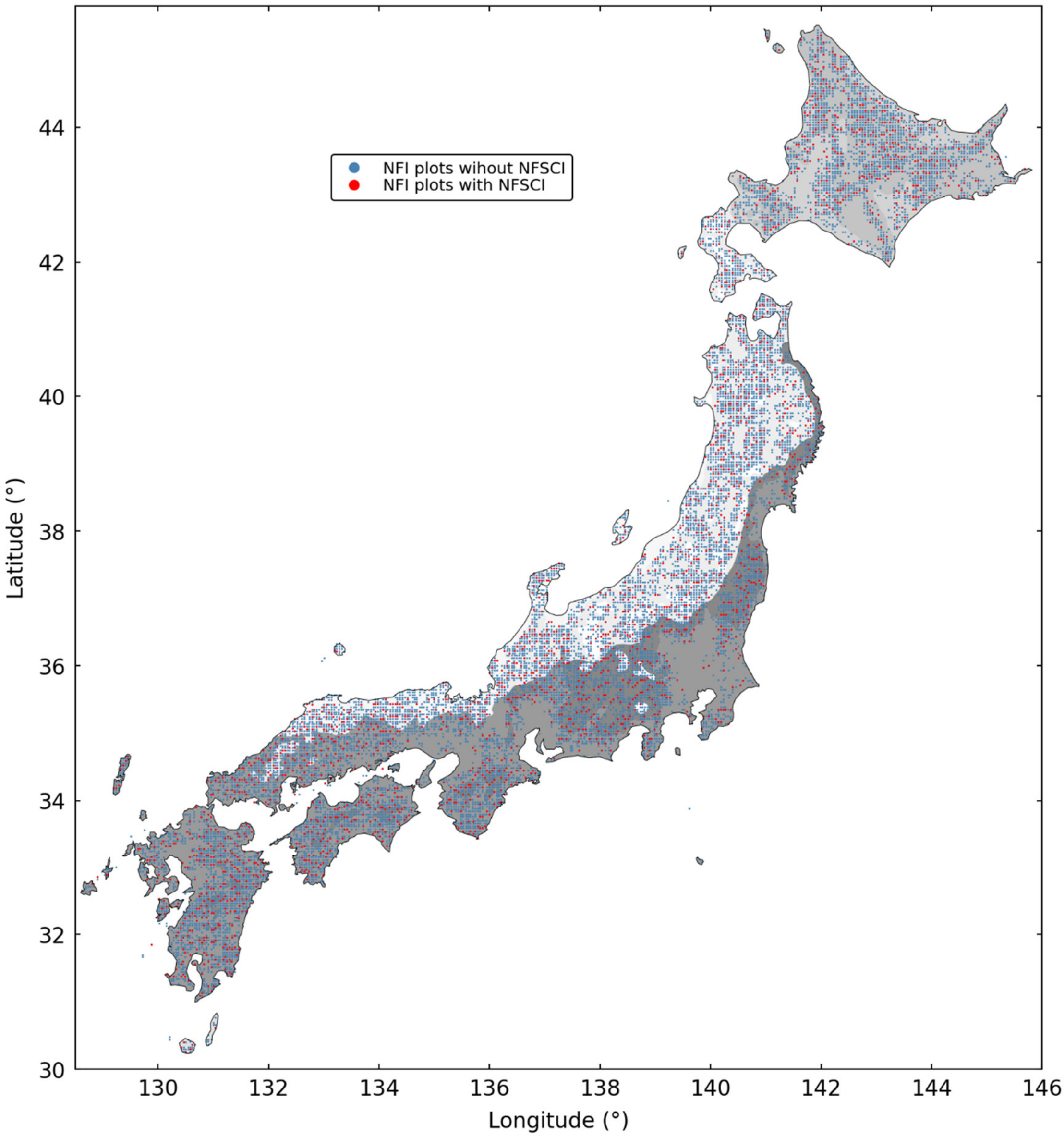

Supplementary Figure 36 | Distribution of the full set of National Forest Inventory (NFI) plots in campaign T2 (n = 12972) and NFI plots in T2 where the National Forest Soil Carbon Inventory (NFSCI) soil inventory was conducted (n = 2215). Ecoregions of Japan after Olson et al. (2001)[66,67,69] are indicated in shades of grey. Ecoregions and coastlines were drawn based on data from RESOLVE[68]. See Figure 1 and Supplementary Tables 5–7 for more details on the ecoregions.

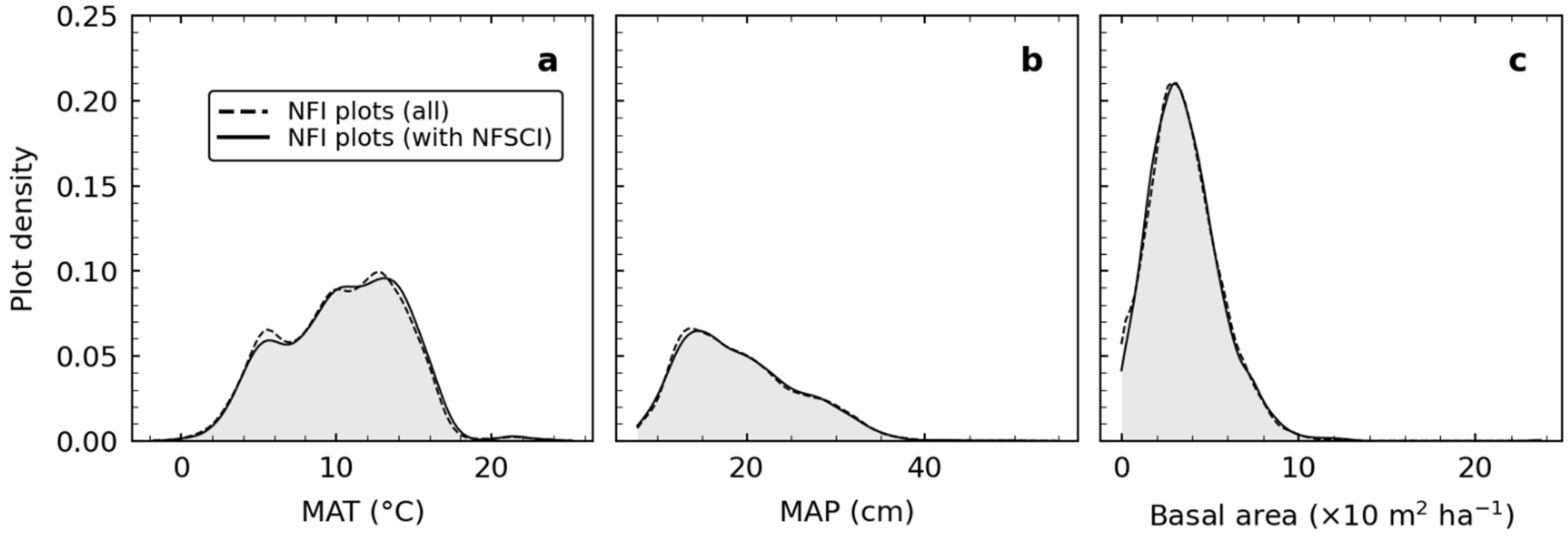

Supplementary Figure 37 | Comparison between the full set of National Forest Inventory (NFI) plots in campaign T2 (n = 12972) and NFI plots in T2 where the National Forest Soil Carbon Inventory (NFSCI) soil inventory was conducted (n = 2215). Distributions of (**a**) mean annual temperature (MAT), (**b**) mean annual precipitation (MAP), and (**c**) basal area are compared. Plot densities were calculated with kernel density estimation, excluding plots not considered in the statistical analysis (see Plot categorization in the Methods section). Source data are provided as a Source Data file.

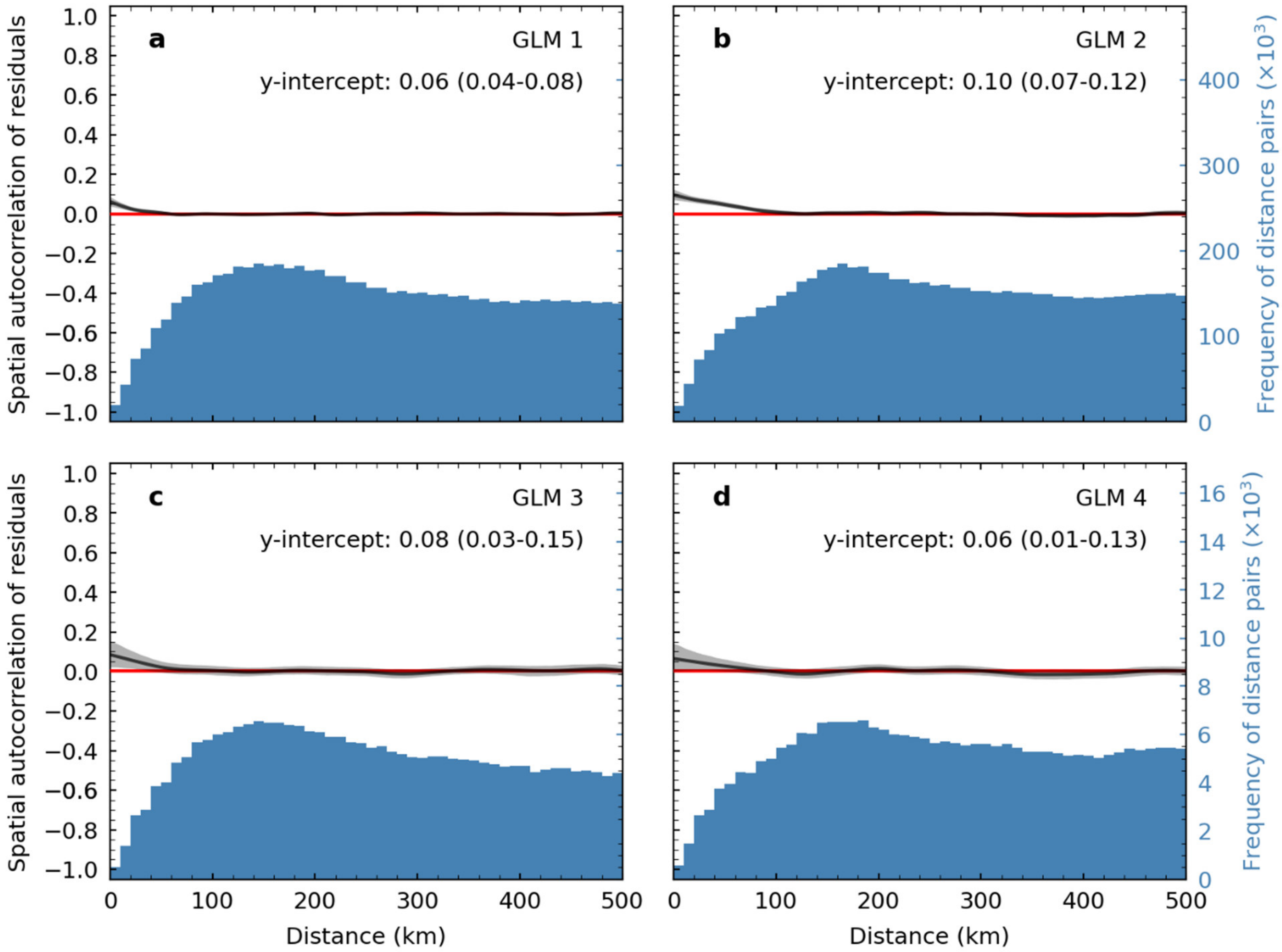

Supplementary Figure 38 | Spatial autocorrelation of model residuals in generalized linear models (GLMs) 1–4 by distance between National Forest Inventory (NFI) plots (≤ 500 km). Spline correlograms for (**a**) GLM 1 (n = 5878 plots), (**b**) GLM 2 (n = 5631 plots), (**c**) GLM 3 (n = 1094 plots), and (**d**) GLM 4 (n = 1068 plots) show the nonparametric covariance function (1000 bootstrap resamples, black line) with 95% confidence intervals (grey shadow) computed at 1000 points between zero and the maximum plot pair distance. Bars (blue) show the frequency of distance pairs in 10 km-wide distance classes. Source data are provided as a Source Data file.

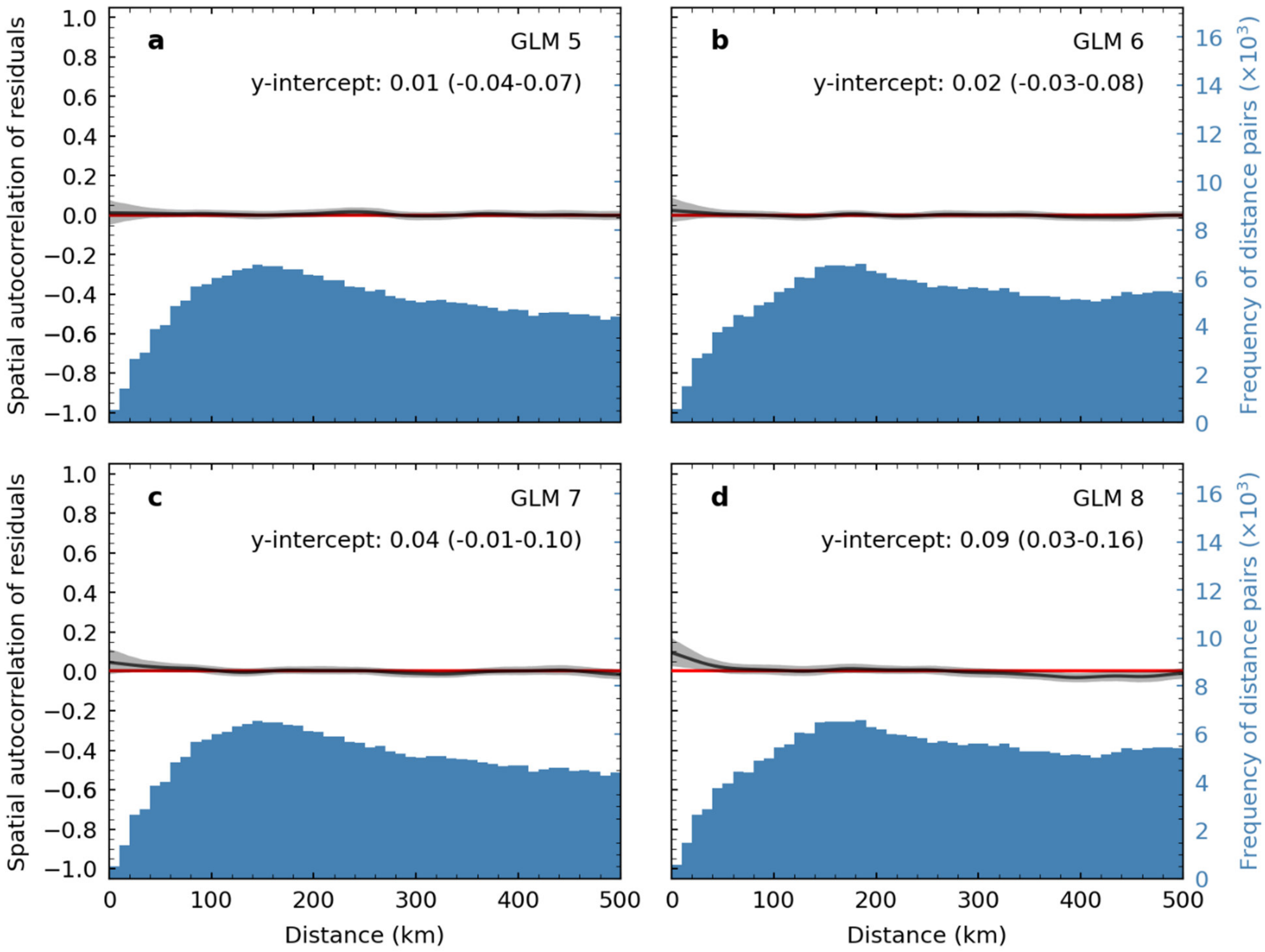

Supplementary Figure 39 | Spatial autocorrelation of model residuals in generalized linear models (GLMs) 5–8 by distance between National Forest Inventory (NFI) plots (≤ 500 km). Spline correlograms for (**a**) GLM 5 (n = 1094 plots), (**b**) GLM 6 (n = 1068 plots), (**c**) GLM 7 (n = 1094 plots), and (**d**) GLM 8 (n = 1068 plots) show the nonparametric covariance function (1000 bootstrap resamples, black line) with 95% confidence intervals (grey shadow) computed at 1000 points between zero and the maximum plot pair distance. Bars (blue) show the frequency of distance pairs in 10 km-wide distance classes. Source data are provided as a Source Data file.

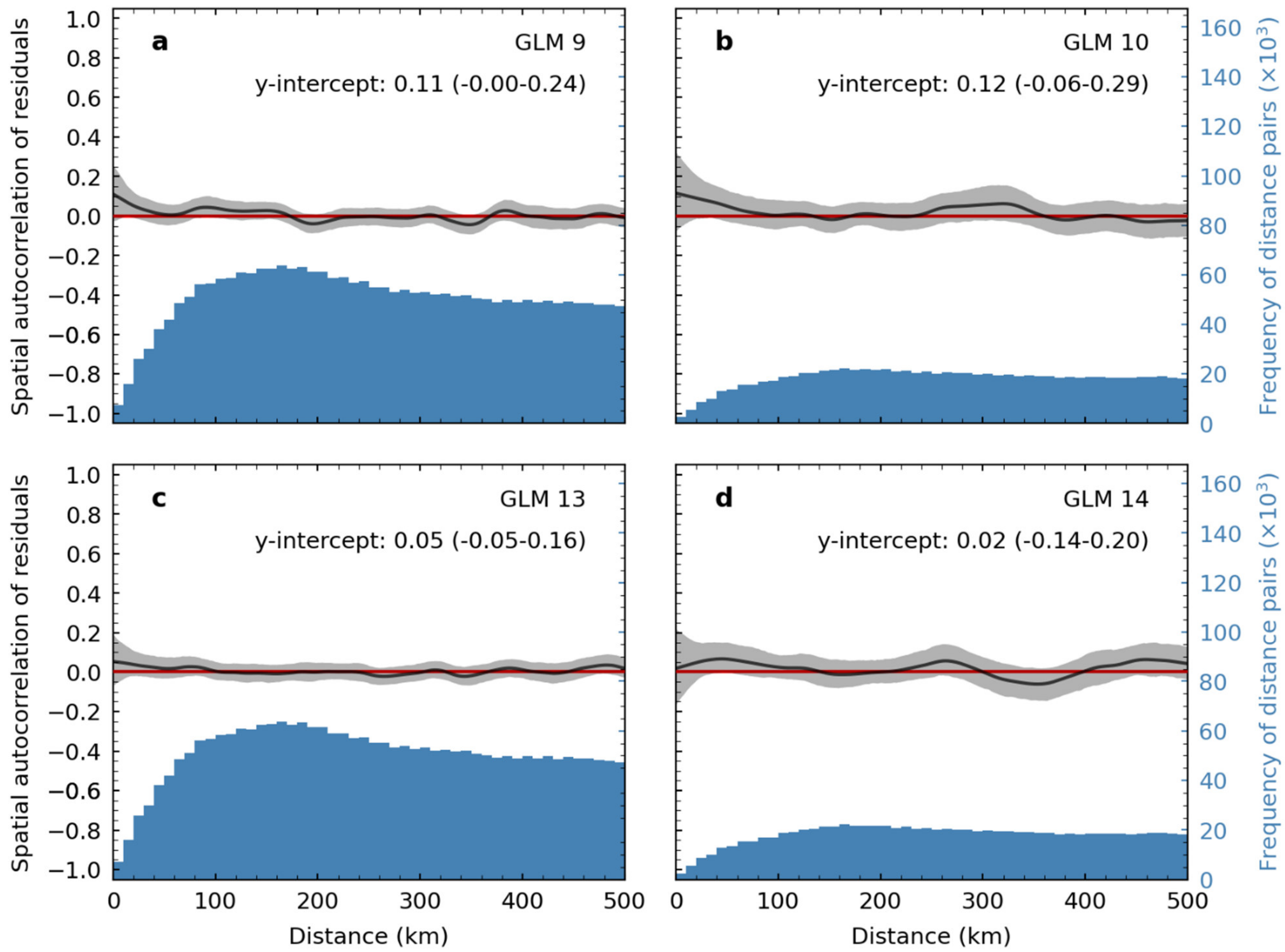

Supplementary Figure 40 | Spatial autocorrelation of model residuals in generalized linear models (GLMs) 9, 10, 13, and 14 by distance between National Forest Inventory (NFI) plots (≤ 500 km). Spline correlograms for (**a**) GLM 9 (n = 3435 plots), (**b**) GLM 10 (n = 2009 plots), (**c**) GLM 13 (n = 3435 plots), and (**d**) GLM 14 (n = 2009 plots) show the nonparametric covariance function (1000 bootstrap resamples, black line) with 95% confidence intervals (grey shadow) computed at 1000 points between zero and the maximum plot pair distance. Bars (blue) show the frequency of distance pairs in 10 km-wide distance classes. Source data are provided as a Source Data file.

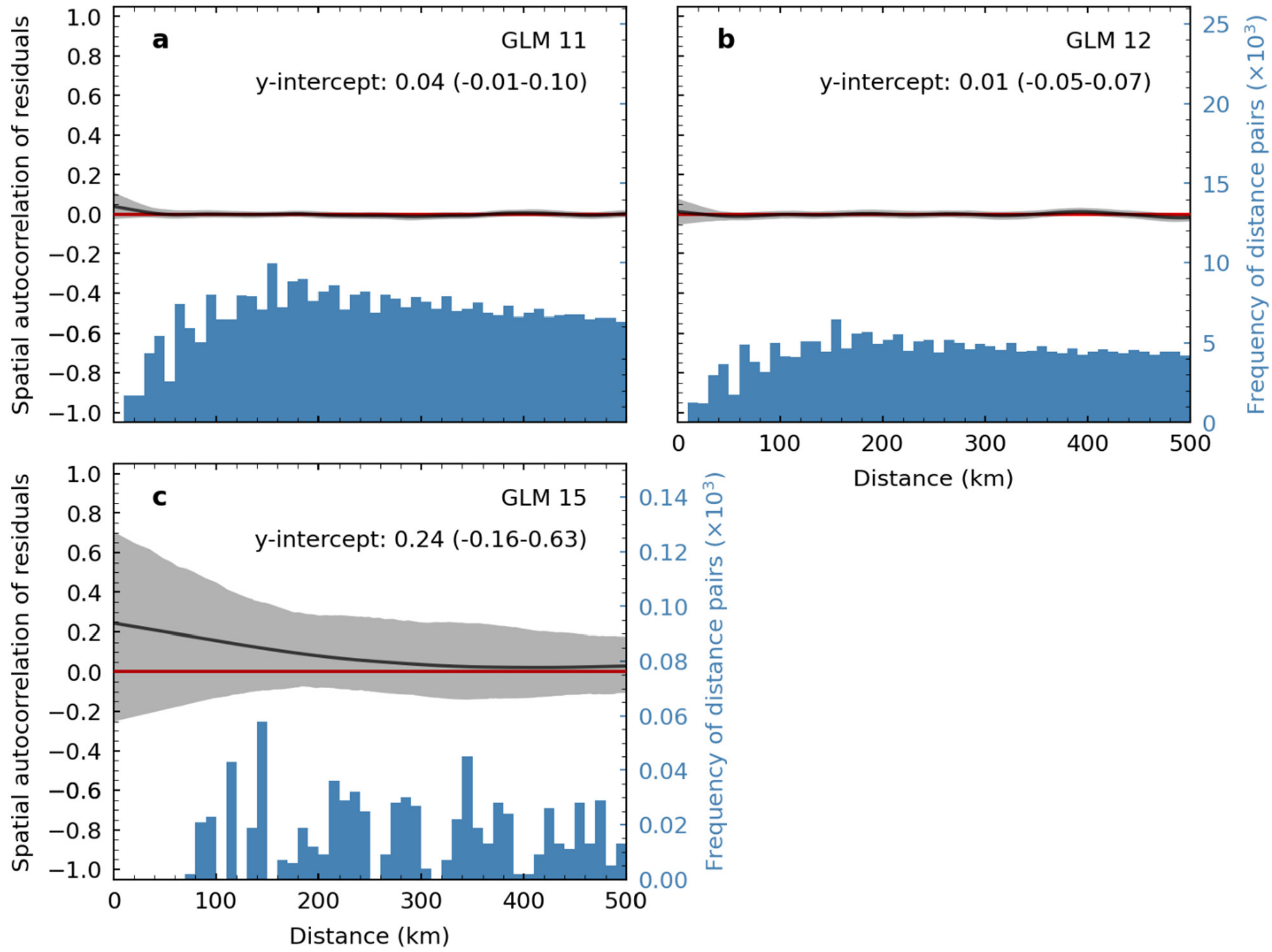

Supplementary Figure 41 | Spatial autocorrelation of model residuals in generalized linear models (GLMs) 11, 12 and 15 by distance between grid cell centers (≤ 500 km). Spline correlograms for (**a**) GLM 11 (n = 1234 grid cells), (**b**) GLM 12 (n = 978 grid cells), and (**c**) GLM 15 (n = 62 grid cells) show the nonparametric covariance function (1000 bootstrap resamples, black line) with 95% confidence intervals (grey shadow) computed at 1000 points between zero and the maximum grid cell pair distance. Bars (blue) show the frequency of distance pairs in 10 km-wide distance classes. Source data are provided as a Source Data file.
