# Supplementary material for: Broad-scale shift in dominance of arbuscular mycorrhizal trees along the Japanese archipelago associated with tree diseases and climate warming
Source: Nat Commun. 2026 Sep 11;17:10731. doi: 10.1038/s41467-026-77711-w (PMC13658775; doi:10.1038/s41467-026-77711-w)
Supplement: Supplementary file 2 [file 41467_2026_77711_MOESM2_ESM.pdf]

Description of Additional Supplementary Files:

Supplementary Data 1. Summary of predictor effects and average marginal effects computed from generalized linear models (GLMs) 1–15.

Supplementary Data 2. Mycorrhizal types assigned in the main analysis and the sensitivity analysis. Data columns are gen: genus; spec: species; myco: mycorrhizal type assigned in the main analysis; source_myco: source of the assigned mycorrhizal type; myco_all: list of all mycorrhizal types reported in Steidinger et al. (2019)[9], Soudzilovskaia et al. (2020)[60], and Soudzilovskaia et al. (2022)[47], contradictory: indication of the presence of contradictory reports; and myco_sX_Y: mycorrhizal type assigned in the sensitivity analysis (model sX with YY reassignments). AM, EM, ErM, NM stand for arbuscular mycorrhizal, ectomycorrhizal, ericoid mycorrhizal, and non-mycorrhizal, respectively. Species-level assignments were obtained from Steidinger et al. (2019)[9], genuslevel assignments from Soudzilovskaia et al. (2020)[63], and species-level diagnoses from Soudzilovskaia et al. (2022)[47].
