# Supplementary material for: Broad-scale shift in dominance of arbuscular mycorrhizal trees along the Japanese archipelago associated with tree diseases and climate warming
Source: Nat Commun. 2026 Sep 11;17:10731. doi: 10.1038/s41467-026-77711-w (PMC13658775; doi:10.1038/s41467-026-77711-w)
Supplement: Supplementary file 3 [file 41467_2026_77711_MOESM5_ESM.pdf]

nature portfolio

Corresponding author(s): Holger Schaefer

Last updated by author(s): Aug 7, 2026

Reporting Summary

Nature Portfolio wishes to improve the reproducibility of the work that we publish. This form provides structure for consistency and transparency in reporting. For further information on Nature Portfolio policies, see our Editorial Policies and the Editorial Policy Checklist.

Statistics

For all statistical analyses, confirm that the following items are present in the figure legend, table legend, main text, or Methods section.

n/a	Confirmed	
☐	☒	The exact sample size (*n*) for each experimental group/condition, given as a discrete number and unit of measurement
☐	☒	A statement on whether measurements were taken from distinct samples or whether the same sample was measured repeatedly
☐	☒	The statistical test(s) used AND whether they are one- or two-sided<br>*Only common tests should be described solely by name; describe more complex techniques in the Methods section.*
☐	☒	A description of all covariates tested
☐	☒	A description of any assumptions or corrections, such as tests of normality and adjustment for multiple comparisons
☐	☒	A full description of the statistical parameters including central tendency (e.g. means) or other basic estimates (e.g. regression coefficient) AND variation (e.g. standard deviation) or associated estimates of uncertainty (e.g. confidence intervals)
☐	☒	For null hypothesis testing, the test statistic (e.g. *F*, *t*, *r*) with confidence intervals, effect sizes, degrees of freedom and *P* value noted<br>*Give P values as exact values whenever suitable.*
☒	☐	For Bayesian analysis, information on the choice of priors and Markov chain Monte Carlo settings
☒	☐	For hierarchical and complex designs, identification of the appropriate level for tests and full reporting of outcomes
☐	☒	Estimates of effect sizes (e.g. Cohen's *d*, Pearson's *r*), indicating how they were calculated

Our web collection on statistics for biologists contains articles on many of the points above.

Software and code

Policy information about availability of computer code

Data collection	Python (version 3.13.5) and Visual Studio Code (version 1.103.2) were used to collect wind and snow water equivalent data. No software was used to collect other data.
Data analysis	Data were compiled using Python (version 3.13.5) in Visual Studio Code (version 1.103.2). Tabular data were processed with the Python package pandas (version 2.3.1) and numpy (version 2.3.1). Vector data (shapefile format) were processed with the Python packages geopandas (version 1.1.1) and shapely (version 2.1.1). Data in GeoTIFF, netCDF, and GRIB formats were accessed using the Python packages rasterio (version 1.4.3), xarray (version 2024.9.0), and pygrib (version 2.1.6), respectively, and converted to vector data. Statistical analyses were conducted using Python packages rpy2 (version 3.5.11) and SciPy (version 1.16.1), and R (version 4.5.1) packages glmmTMB (version 1.1.11), performance (version 0.14.0), DHARMa (version 0.4.7), ggeffects (version 2.2.1), marginaleffects (version 0.26.0), and emmeans (version 1.11.1), and ncf (version 1.3.3). Wind exposure was calculated using the software SAGA (version 9.5.1). Figures were created using Python package matplotlib (version 3.10.3). Maps shown in any figures were plotted using the Python package geopandas (version 1.1.1). The custom computer code used to generate the statistical results has been deposited in Figshare under accession code https://doi.org/10.6084/m9.figshare.29985265.

For manuscripts utilizing custom algorithms or software that are central to the research but not yet described in published literature, software must be made available to editors and reviewers. We strongly encourage code deposition in a community repository (e.g. GitHub). See the Nature Portfolio guidelines for submitting code & software for further information.

Data

Policy information about availability of data

All manuscripts must include a data availability statement. This statement should provide the following information, where applicable:

- Accession codes, unique identifiers, or web links for publicly available datasets
- A description of any restrictions on data availability
- For clinical datasets or third party data, please ensure that the statement adheres to our policy

The plot- and grid-level data generated in this study have been deposited in Figshare under accession code https://doi.org/10.6084/m9.figshare.29985265. Data files are contained in a compressed file archive (Data_for_analysis.zip). The NFI data (http://forestbio.jp), elevation data (https://service.gsi.go.jp/kiban), averaged climate data (https://nlftp.mlit.go.jp/ksj/gml/datalist/KsjTmplt-G02-2022.html), and snow water equivalent data (https://amu.rd.naro.go.jp/wiki_open) for Japan used in this study are available from the relevant government agencies via the links cited in this sentence. The tephra deposition data used in this study are available via Zenodo (https://doi.org/10.5281/zenodo.10846798). The ecoregion data used in this study are available via the Google Earth Engine (https://ecoregions.appspot.com). The plant name data used in this study are available in online indices (http://ylist.info, https://plants.usda.gov). The mycorrhizal type data used in this study are available in the FungalRoot database via PlutoF (https://doi.org/10.4137/EBO.S6271) and the supplementary materials of Steidinger et al. (2019, https://doi.org/10.1038/s41586-019-1128-0) and Soudzilovskaia et al. (2020, https://doi.org/10.1111/nph.16569). The elevation data outside of Japan used in this study are available from a global digital elevation/terrain model (https://doi.org/10.5067/ASTER/ASTGTM.003). The climate time-series data used in this study are available in the TerraClimate dataset (https://doi.org/10.1038/sdata.2017.191). The wind speed data used in this study are available from ERA5-Land (https://doi.org/10.24381/cds.e2161bac). The global tree symbiosis map data used in this study are available in the supplementary materials of Steidinger et al. (2019, https://doi.org/10.1038/s41586-019-1128-0). Details on associated licenses are provided in the Figshare dataset (README_data.txt). Online translation tools may be used to navigate resources in Japanese. The vector data used to draw East Asian country borders in Fig. 1a were obtained from https://www.geoboundaries.org/ under the CC BY 4.0 license (https://creativecommons.org/licenses/by/4.0/). The vector data used to draw ecoregions and Japanese coastlines in all remaining figures were obtained via the Google Earth Engine (https://ecoregions.appspot.com) under the CC BY 4.0 license (https://creativecommons.org/licenses/by/4.0/). Restrictions apply to the availability of N deposition and NFSCI soil data which were used under license for the current study and are not publicly available. N deposition data are available upon request to the corresponding author of Chatani et al. (2025; https://doi.org/10.1007/s44273-025-00052-5). NFSCI soil data are available upon request to the corresponding author of this study and with permission of the Japanese Forestry Agency which owns the data. The agency is currently preparing operations towards publication of the NFSCI data within an unspecified timeframe. Publication of the extensive government-owned data collected on private and public land requires careful consideration of data privacy and safety to prevent exploitation or misuse. Until formal publication by the Japanese Forestry Agency, the NFSCI data can only be made available to qualified individual researchers for the validation and verification of our study results, contingent on approval by the relevant department of the Japanese Forestry Agency. Responses to data access requests may take up to four weeks. Information on predictor effects in GLMs 1–15 is summarized in Supplementary Data 1. Mycorrhizal type assignments are provided in Supplementary Data 2. Source data are provided with this paper.

Research involving human participants, their data, or biological material

Policy information about studies with human participants or human data. See also policy information about sex, gender (identity/presentation), and sexual orientation and race, ethnicity and racism.

Reporting on sex and gender	n/a
Reporting on race, ethnicity, or other socially relevant groupings	n/a
Population characteristics	n/a
Recruitment	n/a
Ethics oversight	n/a

Note that full information on the approval of the study protocol must also be provided in the manuscript.

Field-specific reporting

Please select the one below that is the best fit for your research. If you are not sure, read the appropriate sections before making your selection.

☐ Life sciences ☐ Behavioural & social sciences ☒ Ecological, evolutionary & environmental sciences

For a reference copy of the document with all sections, see nature.com/documents/nr-reporting-summary-flat.pdf

Ecological, evolutionary & environmental sciences study design

All studies must disclose on these points even when the disclosure is negative.

Study description	The study assessed tree symbiosis distributions and their temporal change across the Japanese archipelago, relationships of tree symbiosis distributions to climate and soil nutrient status, and relationships of tree symbiosis distribution changes to the initial vegetation, temperature and precipitation changes, N deposition, and forest disturbances.
Research sample	Tree inventory data in approximately 13,900 forest plots were obtained from Japan's National Forest Inventory (NFI) dataset (version 1, terms 1–4) of Japanese Forestry Agency, Ministry of Agriculture, Forestry and Fisheries of Japan. The NFI data was collected in four

	consecutive 5-year campaigns from 1999 to 2019 (T1–T4) in forests along the Japanese archipelago between 24 and 45°N. NFI plots were set up on the intersections of a 4 × 4 km square grid. Each plot was comprised of an inner (0.01 ha), middle (0.03 ha), and outer section (0.06 ha), where stems with a minimum DBH of 1, 5, and 18 cm were recorded, respectively. For this study, tree inventory data of stems with a DBH ≥5 cm in the inner and middle plot sections were obtained as well as Japanese species names, living statuses (dead versus alive), plot location coordinates (rounded to 0.01°), elevations (rounded to 10 m), investigation dates, stand ages, cutting histories, location changes, occurrence of disturbances (type, observed/not observed), and forest regeneration types. Environmental data for each NFI plot were obtained according to the data availability statement.
Sampling strategy	Published data for the full set of plots in the NFI dataset were obtained without predetermining a sample size.
Data collection	H. Schaefer downloaded the NFI data from http://forestbio.jp, the ecoregion data from https://ecoregions.appspot.com, the mycorrhizal type data from https://doi.org/10.1038/s41586-019-1128-0, https://doi.org/10.1111/nph.16569, and https://doi.org/10.1111/nph.18207, the slope inclination data from https://service.gsi.go.jp/kiban/, the tephra depostion data from https://doi.org/10.1186/s13617-022-00126-x, the temperature and precipitation data from https://nlftp.mlit.go.jp/ and https://doi.org/10.1038/sdata.2017.191, the wind data from https://doi.org/10.24381/cds.e2161bac, the elevation data for wind exposure calculations from https://doi.org/10.5067/ASTER/ASTGTM.003, and the snow water equivalent data from https://amu.rd.naro.go.jp/wiki_open/. N. Yamashita, S. Chatani, and H. Shimadera obtained the nitrogen deposition data from the study "Numerical analysis of factors causing long-term trends and annual variations of sulfur and nitrogen deposition amount in Japan from 2000 to 2020" (https://doi.org/10.1007/s44273-025-00052-5). N. Yamashita and A. Imaya obtained the soil data from the Japanese Forestry Agency.
Timing and spatial scale	Obtained climate data were collected for 1980-2020, NFI data for 1999-2019, and soil data for 2006-2010. All obtained data were collected along the Japanese archipelago.
Data exclusions	Data from plots designated as harvested plantations or bare land, plots with undefined or missing regeneration types, plots younger than 5 years in T1, and data from investigations with erroneous dates were excluded from statistical analyses. Furthermore, a single DBH entry of >300 cm in T4 that was not recorded in T1–T3 was removed due to assumed error in reporting. Finally, the small number of plots (n = 99) assigned to subtropical ecoregions were excluded from statistical analyses. For comparisons of NFI data between campaigns, only plots that were measured repeatedly with valid investigation dates and recorded as neither cut nor relocated were considered.
Reproducibility	Analyses are reproducible with the provided data and code.
Randomization	The sensitivity analysis of the presented study results was done with 100 randomly selected tree species subsets. No other randomization was applied. Analyzed data was obtained from existing datasets.
Blinding	Not applicable.

Did the study involve field work? ☐ Yes ☒ No

Reporting for specific materials, systems and methods

We require information from authors about some types of materials, experimental systems and methods used in many studies. Here, indicate whether each material, system or method listed is relevant to your study. If you are not sure if a list item applies to your research, read the appropriate section before selecting a response.

Materials & experimental systems

n/a	Involved in the study
☒	☐ Antibodies
☒	☐ Eukaryotic cell lines
☒	☐ Palaeontology and archaeology
☒	☐ Animals and other organisms
☒	☐ Clinical data
☒	☐ Dual use research of concern
☒	☐ Plants

Methods

n/a	Involved in the study
☒	☐ ChIP-seq
☒	☐ Flow cytometry
☒	☐ MRI-based neuroimaging

Plants

Seed stocks	*Report on the source of all seed stocks or other plant material used. If applicable, state the seed stock centre and catalogue number. If plant specimens were collected from the field, describe the collection location, date and sampling procedures.*
Novel plant genotypes	*Describe the methods by which all novel plant genotypes were produced. This includes those generated by transgenic approaches, gene editing, chemical/radiation-based mutagenesis and hybridization. For transgenic lines, describe the transformation method, the number of independent lines analyzed and the generation upon which experiments were performed. For gene-edited lines, describe the editor used, the endogenous sequence targeted for editing, the targeting guide RNA sequence (if applicable) and how the editor was applied.*
Authentication	*Describe any authentication procedures for each seed stock used or novel genotype generated. Describe any experiments used to assess the effect of a mutation and, where applicable, how potential secondary effects (e.g. second site T-DNA insertions, mosiacism, off-target gene editing) were examined.*
