# Supplementary material for: Broad-scale shift in dominance of arbuscular mycorrhizal trees along the Japanese archipelago associated with tree diseases and climate warming
Source: Nat Commun. 2026 Sep 11;17:10731. doi: 10.1038/s41467-026-77711-w (PMC13658775; doi:10.1038/s41467-026-77711-w)
Supplement: Supplementary file 4 [file 41467_2026_77711_MOESM6_ESM.pdf]

nature portfolio

Peer Review File

Broad-scale shift in dominance of arbuscular mycorrhizal trees along the Japanese archipelago associated with tree diseases and climate warming

Corresponding Author: Dr Holger Schaefer

This file contains all reviewer reports in order by version, followed by all author rebuttals in order by version.

Version 1:

Reviewer comments:

Reviewer #1

(Remarks to the Author)
This manuscript utilizes data from Japan's National Forest Inventory to assess changes the abundance of AM fungal trees versus EM fungal trees over time. The results are striking—they suggest a shift in abundance of AM trees over EM trees in just 20 years. The authors further conclude that these effects are driven primarily by the introduction of pathogens that primarily attack EM fungal trees. They raise concerns about the potential for changes in nitrogen retention associated with the shift from EM to AM trees. The paper is well written, but I identify some concerns with the manuscript.

First, I found the conclusions regarding changes in N retention to be overstated. To strongly support that a shift in N retention is occurring with the shift to AM trees I would expect data from T1 and T4, however data on N retention was only collected at T2. The data at T2 clearly show changes in N retention between AM and EM tree dominated plots, and therefore suggest that a shift in N retention is likely to be occurring. But it is not evidence that a shift in N retention is occurring as it is a single sampling. I recognize this limitation is driven by the available data, however this limitation is not acknowledged in the text. This is particularly concerning for the paragraph beginning on line 182, and particularly the statement "Our results, hence, link AM tree dominance to N retention in both natural and planted forests across Japan." This statement is not supported by an analysis showing a change in N retention over time.

Second, I was concerned about the comparison to the Steidinger et al paper which dominated the discussion. Without a doubt the difference between the predictions by Steidinger et al and the results observed by the authors merit discussion, but, as the authors highlight throughout the manuscript, there is data lacking from Asian forests generally (and within the Steidinger et al paper). Thus, the results of Steidinger et al for Japanese forests are suspect and therefore the difference between this manuscript and Steidinger at all should not be surprising (and should not merit prime attention within the discussion). Instead, the significant shift in a relatively short period of time (for forests) from AM to EM trees is a significant result that should be reported as the dominant result. (The paragraph beginning on line 198 is more appropriate for beginning the discussion.)

The remainder of my concerns are more minor:

1) Line 67: should "decisive" be replaced by "important"? It seems an odd word choice
2) Lines 72: What do the numbers in parentheses (3 and 4) refer to?
3) Lines 195-7: this seems very speculative
4) Lines 233-4: the initial argument presented in the introduction is that Japanese systems are fundamentally different than European systems, however, this sentence suggests they are the same.
5) Line 238: the sentence structure here is odd. Do the authors mean "Our results should promote follow-up studies…"?
6) Line 240-241: again the sentence structure here is odd. Do the authors mean "the effects of N deposition and tree diseases may interact with each other."?
7) Lines 270-271: Why is a tree that is "both AM and non-mycorrhizal" not simply an AM tree? Please clarify.
8) Line 314: What is tephra deposition? Please clarify for readers who do not come from volcanic islands.
9) Lines 319-327: From my understanding the data on MAT and MAP were obtained from different sources for the absolute values versus the change in MAT and MAP over time. (According to the text absolute values came from the Japan

Meteorological Agency while the change over time came from TerraClimate dataset.) This seems odd—values could differ between the data sets, and differences should be easily calculable from the absolute values. Please explain the choice of data sources.

(Remarks on code availability)

Reviewer #2

(Remarks to the Author)
This study investigated the patterns and drivers of the broad-scale mycorrhizal symbiosis shift along the Japanese archipelago, and found (may at the first time) that the tree diseases was one important factors increasing the AM tree dominance. Overall, I really like the topic and the novel finding of this study, and the manuscript is really well-written and well-organized. Here I have some concerns on the mycorrhizal type assignment and spatial autocorrelation, and I hope these suggestions can help authors to further improve this good manuscript.

Mycorrhizal type assignment: In lines 258-271, the authors said they assigned the mycorrhizal type for tree species based on the Nature paper (species-level) and FungalRoot database (genus-level). Because the Nature paper was published in 2019 and FungalRoot database has updated to 2.0 (see https://doi.org/10.1111/nph.18207), and authors did not provide the detailed species information in their supplemental materials, I guess the mycorrhizal type of some species maybe incorrect. Therefore, given the importance of mycorrhizal type in this study, it is strongly recommended to conduct some further work following one Ecological Monographs paper (https://doi.org/10.1002/ecm.1568) to assess the sensitivity to the misdiagnosis of mycorrhizal types: 1) rechecking all the mycorrhizal types in the FungalRoot v. 2.0, and providing the detailed information for each tree species in your supplemental materials; 2) for the tree species with contradictory reports, you can reassign their mycorrhizal types opposite to the recommended types in your primary (e.g., AM to EM) and reconduct your main analysis to observe their influence to your results.

Spatial autocorrelation: I did not read the information about the distance between your adjacent plots, please give this information in the Method section and guarantee that the autocorrelation issue has been considered in your data analysis.

Other comments:
Title: Because in the Abstract section, you found that “a significant increase in AM tree dominance within 15 years caused by climate warming and the spread of tree diseases”, will the title be better if it includes the “climate warming”?

Lines 45-47: Please rewrite these sentences to explicitly highlight that previous studies and datasets are mainly distributed in North America and Europe.

Lines 74-80: I think these information should be moved to the Method or Result section, and it will be better if you can simply describe your main findings here.

Lines 93-94 and Supplementary Fig. 1 and 2: Your said that “distributions of the AM and EM tree dominance largely mirrored each other” based on the supplementary Fig. 1 and 2, which I think is misleading. First, because AM tree dominance ≈ 1 – EM tree dominance, their distributions should be opposite with each (not mirror each other). Second, I found that in your supplementary Fig. 1 and 2, the color representing the AM or EM tree dominance gradient is opposite (i.e. blue (0) to red (1) for AM tree dominance but red (0) to blue (1) for EM tree dominance), which lead to a similar color distribution in the supplementary Fig. 1 and 2. This will confuse the readers if they did not note the color gradient, so please unify your color gradient.

Using the https://figshare.com/s/67df6acefae0e9d7dd40, I cannot find the available code for this manuscript, please recheck it.

(Remarks on code availability)
I cannot find the available code for this manuscript at https://figshare.com/s/67df6acefae0e9d7dd40

Reviewer #3

(Remarks to the Author)
Please state clearly in the Abstract or first paragraph of the Introduction that you assign mycorrhizal association at the tree species or genus level, then calculate AM tree dominance as the basal-area proportion of AM tree species in each plot. Also state how you treat taxa recorded as both AM and EM (split 50:50). Readers meet “AM tree dominance” before a clear definition.

I do not specialise in advanced statistics. I focused on study design and ecological interpretation,. I assume a reviewer with statistical expertise assessed model specification, diagnostics, and inference.

A large national inventory analysis was conducted, over 13,000 plots across Japan, with repeated measurement across inventory periods. This is a fantastic datset and there were several key findings. AM tree dominance increased over about 15

years by 2.5 ± 1% in natural forests and 1.3 ± 0.6% in planted forests. Disease exposure shows the strongest signal. Infected plots increased by about 9% in both natural and planted forests, versus ~0.4 to 0.6% in non-infected plots. Mean annual temperature associates positively with AM dominance, with stronger temperature sensitivity in cooler northern ecoregions (reported marginal slopes around 3.1–4.4% per 1°C). The observed AM dominance exceeds a published global symbiosis map prediction by ~16%.

The work adds strong evidence on temporal change in mycorrhizal dominance for temperate East Asia using national inventory data and separates natural from planted forests. The climate and soil associations align with published patterns linking warmer climates with higher AM dominance and linking AM dominance with lower soil C:N. The standout contribution is the magnitude of the disease-exposure association relative to N deposition and abiotic disturbance within this dataset.

The results support the main claims about increased AM dominance over time and its associations with warming, disease exposure, and soil C:N. I suggest softer wording for disease, for example "strongly associated with" rather than "drives", because the study remains observational.

Key issues need revision, but they do not prohibit publication. Mycorrhizal assignment uses genus-level classifications for many taxa. Add a short limitation statement and, if available, a brief sensitivity check. Infection status assumes zero where disturbance was not recorded. Under-reporting would bias effects toward the null in "non-infected" plots. State this clearly. Soil C and N derive from a subset of plots. Clarify representativeness and timing alignment. There are some awkward sentences - I have highlighted a few

(Remarks on code availability)

Version 2:

Reviewer comments:

Reviewer #1

(Remarks to the Author)
The authors have done an excellent job of responding to my and other reviewers comments. I have no additional comments.

(Remarks on code availability)

Reviewer #2

(Remarks to the Author)
I am satisfied with the author's revision and thanks for their hard work, this is a great research. Here I only have two minor suggestions about language. 1) In the Abstract section, I think it will be better to reorganize the two sentences "we assessed the dominance of arbuscular mycorrhizal (AM) trees based on basal area proportions in >13,000 forest plots along the Japanese archipelago in temperate East Asia. Mycorrhizal types were assigned at the species or genus level", to first introduce mycorrhizal type identify and then assess AM tree dominance. 2) in line 277, "in particular" to "particularly".

(Remarks on code availability)

Reviewer #3

(Remarks to the Author)
The authors addressed all my comments, and those of the other reviewers carefully and made appropriate changes in the manuscript. I believe the manuscript is acceptable for publication in its current form

(Remarks on code availability)

Open Access This Peer Review File is licensed under a Creative Commons Attribution 4.0 International License, which permits use, sharing, adaptation, distribution and reproduction in any medium or format, as long as you give appropriate credit to the original author(s) and the source, provide a link to the Creative Commons license, and indicate if changes were made.
In cases where reviewers are anonymous, credit should be given to 'Anonymous Referee' and the source.
The images or other third party material in this Peer Review File are included in the article's Creative Commons license, unless indicated otherwise in a credit line to the material. If material is not included in the article's Creative Commons license and your intended use is not permitted by statutory regulation or exceeds the permitted use, you will need to obtain permission directly from the copyright holder.
To view a copy of this license, visit https://creativecommons.org/licenses/by/4.0/

Response to the reviewers' comments

Reviewer #1 (Remarks to the Author):

This manuscript utilizes data from Japan's National Forest Inventory to assess changes the abundance of AM fungal trees versus EM fungal trees over time. The results are striking—they suggest a shift in abundance of AM trees over EM trees in just 20 years. The authors further conclude that these effects are driven primarily by the introduction of pathogens that primarily attack EM fungal trees. They raise concerns about the potential for changes in nitrogen retention associated with the shift from EM to AM trees. The paper is well written, but I identify some concerns with the manuscript.

Response 1.1: We thank the reviewer for the positive assessment. Based on the helpful comments, the manuscript was revised to better communicate data limitations and the need for further study, improve language and text structure, and better explain technical terms and the choice of data sources.

First, I found the conclusions regarding changes in N retention to be overstated. To strongly support that a shift in N retention is occurring with the shift to AM trees I would expect data from T1 and T4, however data on N retention was only collected at T2. The data at T2 clearly show changes in N retention between AM and EM tree dominated plots, and therefore suggest that a shift in N retention is likely to be occurring. But it is not evidence that a shift in N retention is occurring as it is a single sampling. I recognize this limitation is driven by the available data, however this limitation is not acknowledged in the text. This is particularly concerning for the paragraph beginning on line 182, and particularly the statement "Our results, hence, link AM tree dominance to N retention in both natural and planted forests across Japan." This statement is not supported by an analysis showing a change in N retention over time.

Response 1.2: We agree that time series data is necessary to support that a shift in N retention is occurring with a shift in AM tree dominance. To avoid giving the impression that a change in N retention over time was observed, the sentence "Our results, hence, link AM tree dominance to N retention in both natural and planted forests across Japan." was removed (L232). To more clearly communicate that a shift in N retention was only indicated by data from T2, the sentence "Long-term shifts in AM tree dominance along the Japanese archipelago may, hence, entail changes in forest nutrient cycling, in particular soil N retention." was rephrased as "The relationship of AM tree dominance to the ratio of soil C and N stocks, hence, indicates that the AM tree dominance shift along

the Japanese archipelago will decrease N retention." (L236-238). In the following sentences, the data limitation and the need for further study were clarified (L238-240): "Nevertheless, data on soil C and N stocks in our study were limited to NFI campaign T2. To strongly support a link between AM tree dominance and N retention, follow-up studies are needed that analyze time series data."
Furthermore, to avoid any misunderstanding, "link between AM tree dominance and soil nutrient status" was replaced with "relationship between AM tree dominance and soil nutrient status in NFI campaign T2" in the concluding paragraph of the Discussion (L267-268), ", linking N availability to N retention" was added in L232, and other smaller adjustments were made (L25-26, L268).

Second, I was concerned about the comparison to the Steidinger et al paper which dominated the discussion. Without a doubt the difference between the predictions by Steidinger et al and the results observed by the authors merit discussion, but, as the authors highlight throughout the manuscript, there is data lacking from Asian forests generally (and within the Steidinger et al paper). Thus, the results of Steidinger et al for Japanese forests are suspect and therefore the difference between this manuscript and Steidinger at all should not be surprising (and should not merit prime attention within the discussion). Instead, the significant shift in a relatively short period of time (for forests) from AM to EM trees is a significant result that should be reported as the dominant result. (The paragraph beginning on line 198 is more appropriate for beginning the discussion.)

Response 1.3: We agree that the observed shift from EM to AM trees should be better highlighted in the Discussion section. In line with this comment and the last comment by Reviewer #3, the two related paragraphs were moved to the beginning of the Discussion section (L167-196). When restructuring the Discussion section, two sentences in the subsequent paragraphs were revised to reference the shift from EM to AM trees (L197-198, L217-218). The discussion of the difference to Steidinger et al. (2019) was moved to a later part of the Discussion section (L241-252).

The remainder of my concerns are more minor:

1) Line 67: should "decisive" be replaced by "important"? It seems an odd word choice

Response 1.4: "Decisive" was replaced with "important" (L74).

2) Lines 72: What do the numbers in parentheses (3 and 4) refer to?

Response 1.5: The numbers are counting hypotheses 3 and 4. To make this clearer, counters were

changed to H1-H4 and moved to the end of each hypothesis (L73, L77, L79, and L81). In line with this change, sentences in the Results section were also revised slightly (L107, L117, L130, and L141).

3) Lines 195-7: this seems very speculative

Response 1.6: Please see the second response.

4) Lines 233-4: the initial argument presented in the introduction is that Japanese systems are fundamentally different than European systems, however, this sentence suggests they are the same.

Response 1.7: The assumption that PWD will cause severe disturbance to European forests is not made based on the similarity between Japanese and European forests. Rather, the assumption is made in the cited studies by Back et al. (2024) and Soliman et al. (2012) based on models of the local climate, disease spread, and host distribution in Europe. The sentence was revised to clarify this (L272).

5) Line 238: the sentence structure here is odd. Do the authors mean "Our results should promote follow-up studies…"?

Response 1.8: Yes, that is what we meant. The sentence was revised accordingly (L276).

6) Line 240-241: again the sentence structure here is odd. Do the authors mean "the effects of N deposition and tree diseases may interact with each other."?

Response 1.9: We meant that N deposition and tree diseases may both be important factors of tree symbiosis distribution changes. The sentence was revised accordingly (L277-278).

7) Lines 270-271: Why is a tree that is "both AM and non-mycorrhizal" not simply an AM tree? Please clarify.

Response 1.10: Distinction between AM and AM-NM trees is common in related research, including the databases that were used for mycorrhizal type assignment (Brundrett and Tedersoo 2019, Steidinger et al. 2019, Soudzilovskaia et al. 2020). AM-NM trees belong to genera with inconsistent or low records of AM fungal colonization, whose capacity to form mycorrhiza may depend on, e.g., habitat conditions. A short explanation was added (L311-313). The phrase "both AM and non-mycorrhizal" was indeed confusing and replaced with "weakly or facultatively AM" (L311).

AM-NM species were rare in our dataset (Supplementary Data 2), and reclassifying AM-NM species

as AM or vice versa had little influence on our study results in the newly added sensitivity analysis (Supplementary Figs. 28-34). For details, see our second response to Reviewer #2.

8) Line 314: What is tephra deposition? Please clarify for readers who do not come from volcanic islands.

Response 1.11: Tephra is fragmental material ejected during volcanic eruptions. A short explanation was added (L372-373).

9) Lines 319-327: From my understanding the data on MAT and MAP were obtained from different sources for the absolute values versus the change in MAT and MAP over time. (According to the text absolute values came from the Japan Meteorological Agency while the change over time came from TerraClimate dataset.) This seems odd—values could differ between the data sets, and differences should be easily calculable from the absolute values. Please explain the choice of data sources.

Response 1.12: For changes in climate variables over time, it is common practice to use climate reanalysis data such as TerraClimate. Climate reanalysis uses modelling approaches to generate consistent time series data of climate variables (see e.g. https://climate.copernicus.eu/climate-reanalysis). This reduces uncertainty in the data from e.g. changes in data availability (relocation of weather stations, replacement of equipment, etc.) or data processing. TerraClimate was chosen for its high spatial resolution and accessibility among reanalysis datasets. TerraClimate makes use of multiple data sources, including the Japan Meteorological Agency. Please see the cited article by Abatzoglou et al. (2018) for details. A short explanation was added to the manuscript (L384-385).

Reviewer #2 (Remarks to the Author):

This study investigated the patterns and drivers of the broad-scale mycorrhizal symbiosis shift along the Japanese archipelago, and found (may at the first time) that the tree diseases was one important factors increasing the AM tree dominance. Overall, I really like the topic and the novel finding of this study, and the manuscript is really well-written and well-organized. Here I have some concerns on the mycorrhizal type assignment and spatial autocorrelation, and I hope these suggestions can help authors to further improve this good manuscript.

Response 2.1: We thank the reviewer for the positive feedback and the helpful suggestions. The suggested analyses and revisions helped us to verify and better communicate our study results.

Mycorrhizal type assignment: In lines 258-271, the authors said they assigned the mycorrhizal type for tree species based on the Nature paper (species-level) and FungalRoot database (genus-level). Because the Nature paper was published in 2019 and FungalRoot database has updated to 2.0 (see https://doi.org/10.1111/nph.18207), and authors did not provide the detailed species information in their supplemental materials, I guess the mycorrhizal type of some species maybe incorrect. Therefore, given the importance of mycorrhizal type in this study, it is strongly recommended to conduct some further work following one Ecological Monographs paper (https://doi.org/10.1002/ecm.1568) to assess the sensitivity to the misdiagnosis of mycorrhizal types: 1) rechecking all the mycorrhizal types in the FungalRoot v. 2.0, and providing the detailed information for each tree species in your supplemental materials; 2) for the tree species with contradictory reports, you can reassign their mycorrhizal types opposite to the recommended types in your primary (e.g., AM to EM) and reconduct your main analysis to observe their influence to your results.

Response 2.2: We agree that testing the sensitivity to potentially incorrect mycorrhizal type assignments would help to verify our study results. In line with the reviewer's suggestion, mycorrhizal diagnoses in the updated version of FungalRoot were downloaded and checked for all tree species in the NFI dataset. We made sure to download the FungalRoot data again later in the revision process, to provide the most recent information. The mycorrhizal type assigned in our study and all mycorrhizal types reported in Steidinger et al. (2019), Soudzilovskaia et al. (2020), and FungalRoot 2.0 were provided in Supplementary Data 2. For clarity, the relationship between the genus-level assignments in Soudzilovskaia et al. (2020) and FungalRoot was explained (L308-309). Overall, 37 species had contradictory reports as described in the Methods section (L314-325).

Based on the collected information, the sensitivity of our study results to potentially incorrect mycorrhizal type assignments was tested in line with the suggested *Ecological Monographs* article. However, instead of only looking at the unlikely scenario in which all tree species with contradictory reports have different mycorrhizal types, reassignments were made for randomly selected subsets of tree species in 100 iterations. While the reassignment of mycorrhizal types had overall little influence on our study results, some effects were observed for the reassignment of *Castanopsis* species in GLM 12. The sensitivity analysis is described in L479-483. Reassigned mycorrhizal types are listed in Supplementary Data 2. Analysis results are shown in Supplementary Figs. 28-34 (and their Source Data), described in L154-165, and discussed in L253-264.

Spatial autocorrelation: I did not read the information about the distance between your adjacent plots, please give this information in the Method section and guarantee that the autocorrelation issue has been considered in your data analysis.

Response 2.3: NFI plots were set up on the intersections of a 4 × 4 km square grid. The information was added (L288-289). Spatial autocorrelation was considered in GLMs by including *ecoregion* as a random effect, which accounted for the spatial interdependency of plots in each ecoregion. To show that spatial autocorrelation in GLMs was low, the spatial autocorrelation by distance between plots and grid cells was estimated (Supplementary Figs. 38-41) and briefly described (L476-479).

When estimating the spatial autocorrelation in GLM 15, which compared the AM tree dominance between this study and Steidinger et al. (2019), slightly elevated levels of spatial autocorrelation were found, likely because GLM 15 did not contain the *ecoregion* random effect. GLM 15 was revised by including *ecoregion* as a random effect and removing two grid cells in subtropical ecoregions, in line with the study area of all other GLMs. These revisions had little effect on model outcomes. The difference in AM tree dominance between studies was still significant and estimated at 16.1%. Information on GLM 15 was updated accordingly (L241, L244-245, Supplementary Data 1, Supplementary Fig. 27).

Other comments:

Title: Because in the Abstract section, you found that "a significant increase in AM tree dominance within 15 years caused by climate warming and the spread of tree diseases", will the title be better if it includes the "climate warming"?

Response 2.4: "and climate warming" was added to the title as suggested. To make the title fit within the word limit (15 words), "a" was removed and the word order was changed.

Lines 45-47: Please rewrite these sentences to explicitly highlight that previous studies and datasets are mainly distributed in North America and Europe.

Response 2.5: Revisions were made to clarify that the reported findings were made in studies on North American forests (L45-47). That the finding in L44-45 is mainly based on data from North America and Europe is already mentioned in L48-50.

Lines 74-80: I think these information should be moved to the Method or Result section, and it will

be better if you can simply describe your main findings here.

Response 2.6: Information on additional model predictors was moved to the Methods section (L458-462). Only the information that variation by ecoregion was accounted for was kept in its location (L81-82), since the Results section frequently references ecoregions. Furthermore, a brief description of the main findings was added to L83-88.

Lines 93-94 and Supplementary Fig. 1 and 2: Your said that "distributions of the AM and EM tree dominance largely mirrored each other" based on the supplementary Fig. 1 and 2, which I think is misleading. First, because AM tree dominance ≈ 1 − EM tree dominance, their distributions should be opposite with each (not mirror each other). Second, I found that in your supplementary Fig. 1 and 2, the color representing the AM or EM tree dominance gradient is opposite (i.e. blue (0) to red (1) for AM tree dominance but red (0) to blue (1) for EM tree dominance), which lead to a similar color distribution in the supplementary Fig. 1 and 2. This will confuse the readers if they did not note the color gradient, so please unify your color gradient.

Response 2.7: We agree that the phrase "mirror each other" may be confusing to readers. The sentence was revised as suggested (L101). In the original Supplementary Fig. 2, the color gradient was reversed to make it easier for readers to compare the color distributions in Supplementary Figs. 1 and 2. We agree that this may also be confusing. The color gradient was unified as suggested (Supplementary Figs. 1-4).

Using the https://figshare.com/s/67df6acefae0e9d7dd40, I cannot find the available code for this manuscript, please recheck it.

Reviewer #2 (Remarks on code availability):

I cannot find the available code for this manuscript at https://figshare.com/s/67df6acefae0e9d7dd40

Response 2.8: Provided code files may have been difficult to identify. To improve code accessibility, files were renamed and collected in a compressed file archive called "Code_for_analysis.zip". The code consists of the main analysis script (data_analysis_script.py) and two collections of functions (python_functions.py, R_functions.R).

The code is mainly written in Python, which is the main programming language used in this project due to its advanced features for processing spatial data. R functions were used for statistical testing, as R offers more powerful statistical tools. Users only need to interact with the main analysis script

(data_analysis_script.py) to run all code. Python scripts may be inspected with Visual Studio Code, R scripts with Visual Studio Code or RStudio. Detailed instructions on how the code is executed are provided with the code files (README_code.txt). Furthermore, the Code Availability section was revised to better describe the code (L509-514).

Reviewer #3 (Remarks to the Author):

Please state clearly in the Abstract or first paragraph of the Introduction that you assign mycorrhizal association at the tree species or genus level, then calculate AM tree dominance as the basal-area proportion of AM tree species in each plot. Also state how you treat taxa recorded as both AM and EM (split 50:50). Readers meet "AM tree dominance" before a clear definition.

Response 3.1: We agree that, for clarity, AM tree dominance should be explained earlier in the manuscript. Information was added to the abstract (L20-22) and the end of the Introduction section right after the study goal (L68-71).

I do not specialise in advanced statistics. I focused on study design and ecological interpretation,. I assume a reviewer with statistical expertise assessed model specification, diagnostics, and inference.

A large national inventory analysis was conducted, over 13,000 plots across Japan, with repeated measurement across inventory periods. This is a fantastic datset and there were several key findings. AM tree dominance increased over about 15 years by 2.5 ± 1% in natural forests and 1.3 ± 0.6% in planted forests. Disease exposure shows the strongest signal. Infected plots increased by about 9% in both natural and planted forests, versus ~0.4 to 0.6% in non-infected plots. Mean annual temperature associates positively with AM dominance, with stronger temperature sensitivity in cooler northern ecoregions (reported marginal slopes around 3.1−4.4% per 1° C). The observed AM dominance exceeds a published global symbiosis map prediction by ~16%.

The work adds strong evidence on temporal change in mycorrhizal dominance for temperate East Asia using national inventory data and separates natural from planted forests. The climate and soil associations align with published patterns linking warmer climates with higher AM dominance and linking AM dominance with lower soil C:N. The standout contribution is the magnitude of the disease-exposure association relative to N deposition and abiotic disturbance within this dataset.

Response 3.2: We thank the reviewer for the positive evaluation. Based on the helpful comments, we improved the structure and language of the manuscript and clarified the representativeness and

limitations of the analyzed data.

The results support the main claims about increased AM dominance over time and its associations with warming, disease exposure, and soil C:N. I suggest softer wording for disease, for example "strongly associated with" rather than "drives", because the study remains observational.

Response 3.3: In line with the observational nature of our study, more careful language was used. "drive" in the title and "cause" in the abstract were replaced with "associated with" (L4, L23). In the Discussion section, "drivers" was replaced with "apparent drivers" (L171) and "findings revealed" was replaced with "findings indicate" (L265).

Key issues need revision, but they do not prohibit publication. Mycorrhizal assignment uses genus-level classifications for many taxa. Add a short limitation statement and, if available, a brief sensitivity check.

Response 3.4: A short limitation statement for genus-level assignments was added (L314-317), followed by information on contradictory reports for the mycorrhizal types of tree species in the NFI dataset (L317-325). Furthermore, the sensitivity of our study results to potentially incorrect mycorrhizal type assignments was tested (L479-483). Analysis results are shown in Supplementary Figs. 28-34, described in L154-165, and discussed in L253-264. For details, please see our second response to Reviewer #2.

Infection status assumes zero where disturbance was not recorded. Under-reporting would bias effects toward the null in "non-infected" plots. State this clearly.

Response 3.5: A remark in line with the comment was added (L403-404).

Soil C and N derive from a subset of plots. Clarify representativeness and timing alignment.

Response 3.6: Representativeness and timing were clarified in the Methods section (L358-362) and the newly added Supplementary Figs. 36 and 37.

There are some awkward sentences - I have highlighted a few

(copied from attachment) L42-43: but how - tree species tend to be one or the other, so the EM/AM wont change unless the tree species change - it is a chicken or egg, do the tees respond to the climate

and die out thus allowing other colonisers with different fungi or do the fungi die out and thus the trees die.

Response 3.7: The cited study predicts a shift in EM and AM trees due to climate warming. For clarification, “symbiosis” was replaced with “trees” (L44).

(copied from attachment) L60-61: again surly they effect tree species distributions before they effect the symbiosis

Response 3.8: The sentence was revised to clearly state the effect on tree species distributions (L63-64).

(copied from attachment) L154-156: but is that due to changing climate or infact lack of basline understanding of where the tree species were distributed?

Response 3.9: There is likely a contribution of both. The paragraph was revised to address this (L245-250).

(copied from attachment) L160-162: this is an awkward sentence. starting with That the is unusual maybe: The larger effect size of MAT than MAP was expected because, in precipitation-rich climates, soil moisture likely does not limit SOM mineralisation.

Response 3.10: The sentence was revised accordingly (L201-203).

(copied from attachment) L162-164: another awkward sentence: Similarly, mycorrhizal buffering of water stress, which could increase the apparent influence of MAP, likely contributed little in this study area because drought has been limited.

Response 3.11: The sentence was revised accordingly (L203-205).

(copied from attachment) L198-199: This is basically you most important finding finding and it is buried in the discussion

Response 3.12: We agree that the observed shift in AM tree dominance should be better highlighted in the Discussion section. The two related paragraphs were moved to the beginning of the Discussion section (L167-196). The discussion of the difference to Steidinger et al. (2019) was moved to a later part of the Discussion section (L241-252). Please also see our third response to Reviewer #1.

Response to the reviewers' comments

Reviewer #1:

The authors have done an excellent job of responding to my and other reviewers comments. I have no additional comments.

Response: Thank you for the positive feedback.

Reviewer #2:

I am satisfied with the author's revision and thanks for their hard work, this is a great research. Here I only have two minor suggestions about language. 1) In the Abstract section, I think it will be better to reorganize the two sentences "we assessed the dominance of arbuscular mycorrhizal (AM) trees based on basal area proportions in >13,000 forest plots along the Japanese archipelago in temperate East Asia. Mycorrhizal types were assigned at the species or genus level", to first introduce mycorrhizal type identify and then assess AM tree dominance. 2) in line 277, "in particular" to "particularly".

Response: Thank you for the positive evaluation. The two sentences in the abstract were reorganized to first introduce the mycorrhizal type assignment and then the dominance calculation (L20-22). Furthermore, "in particular" was replaced with "particularly" (L288).

Reviewer #3:

The authors addressed all my comments, and those of the other reviewers carefully and made appropriate changes in the manuscript. I believe the manuscript is acceptable for publication in its current form

Response: Thank you for the positive feedback.
